# Development of Novel Alaninamide Derivatives with Anticonvulsant Activity and Favorable Safety Profiles in Animal Models

**DOI:** 10.3390/ijms25189861

**Published:** 2024-09-12

**Authors:** Michał Abram, Marcin Jakubiec, Paulina Koczurkiewicz-Adamczyk, Agata Doroz-Płonka, Anna Rapacz, Krzysztof Kamiński

**Affiliations:** 1 Department of Medicinal Chemistry, Faculty of Pharmacy, Jagiellonian University Medical College, Medyczna 9, 30-688 Kraków, Poland; marcin.jakubiec@uj.edu.pl (M.J.); k.kaminski@uj.edu.pl (K.K.); 2 Department of Pharmaceutical Biochemistry, Faculty of Pharmacy, Jagiellonian University Medical College, Medyczna 9, 30-688 Kraków, Poland; paulina.koczurkiewicz@uj.edu.pl; 3 Department of Technology and Biotechnology of Drugs, Faculty of Pharmacy, Jagiellonian University Medical College, Medyczna 9, 30-688 Kraków, Poland; a.doroz-plonka@uj.edu.pl; 4 Department of Pharmacodynamics, Faculty of Pharmacy, Jagiellonian University Medical College, Medyczna 9, 30-688 Kraków, Poland

**Keywords:** hybrid compounds, anticonvulsant activity, multimodal/multitarget compounds, amino acids derivatives, binding/functional assays, tox studies

## Abstract

In our current study, we developed a focused series of original ((benzyloxy)benzyl)propanamide derivatives that demonstrated potent activity across in vivo mouse seizure models, specifically, maximal electroshock (MES) and 6 Hz (32 mA) seizures. Among these derivatives, compound **5** emerged as a lead molecule, exhibiting robust protection following intraperitoneal (i.p.) injection, as follows: ED_50_ = 48.0 mg/kg in the MES test, ED_50_ = 45.2 mg/kg in the 6 Hz (32 mA) test, and ED_50_ = 201.3 mg/kg in the 6 Hz (44 mA) model. Additionally, compound **5** displayed low potential for inducing motor impairment in the rotarod test (TD_50_ > 300 mg/kg), indicating a potentially favorable therapeutic window. In vitro toxicity assays further supported its promising safety profile. We also attempted to identify a plausible mechanism of action of compound **5** by applying both binding and functional in vitro studies. Overall, the data obtained for this lead molecule justifies the more comprehensive preclinical development of compound **5** as a candidate for a potentially broad-spectrum and safe anticonvulsant.

## 1. Introduction

Epilepsy is a chronic neurological disorder characterized by a persistent predisposition to experience unprovoked seizures, affecting individuals across all ages, ethnicities, social strata, and regions [1]. The complex nature of epilepsy pathogenesis complicates its treatment, and the effectiveness of existing antiseizure medications (ASMs) is often limited. Notably, as many as 30% of epilepsy patients suffer from drug-resistant epilepsy (DRE), showing no improvement with currently available ASMs [2]. This resistance poses a significant health threat, as DRE is associated with sudden unexpected death in epilepsy (SUDEP) and various psychiatric, psychosocial, and medical issues, significantly impacting patients’ overall quality of life [3].

An emerging therapeutic approach for DRE involves the development of multimodal (multitarget/multifunctional) compounds capable of interacting with multiple molecular targets simultaneously. This strategy aims to address the limitations of polytherapy, which is commonly used for DRE and is associated with increased risks of drug–drug interactions and various side effects, leading to poor adherence [4,5]. These multitarget compounds can be designed as hybrid or chimeric molecules, integrating several pharmacophores into one molecule, often resulting in a broader and more synergistic mechanism of action. This multimechanistic approach is gaining traction not only in epilepsy treatment but also in managing neurodegenerative diseases (such as Alzheimer’s and Parkinson’s), as well as in depression, diabetes, metabolic and inflammatory disorders, cancer, and neurological conditions like neuropathic pain [6,7,8,9,10].

**Figure 1:**
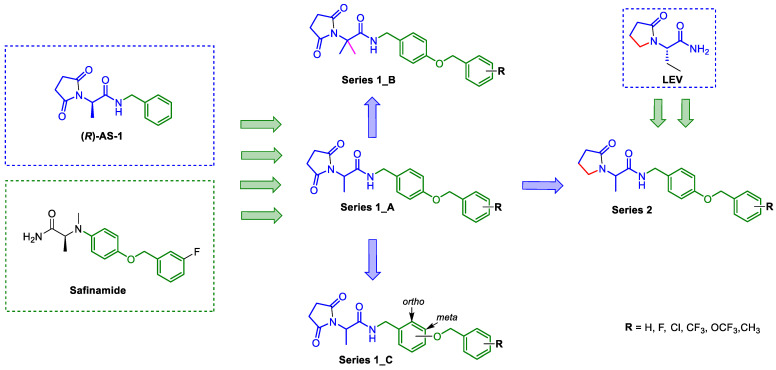
Lead compound (***R***)**-AS-1**, [11] and design approach of hybrid compounds obtained in the current studies. The common structural elements forming the new hybrids are highlighted in color.

Epilepsy is a prevalent neurological disorder necessitating the development of novel ASMs with unique mechanisms of action and minimal adverse effects. One promising candidate, (***R***)**-AS-1** (Figure 1), was obtained previously in our research. This compound has demonstrated broad-spectrum antiseizure efficacy across various acute mouse seizure models, including maximal electroshock (MES), 6 Hz (32/44 mA), subcutaneous pentylenetetrazole (*sc*PTZ), and chronic PTZ-kindling [11]. Remarkably, (***R***)**-AS-1** has shown a significant separation between antiseizure activity and CNS-related adverse effects, highlighting its therapeutic potential. In vitro studies have revealed that (***R***)**-AS-1** acts as a positive allosteric modulator (PAM) of the glutamate transporter EAAT2, enhancing glutamate uptake in primary glia cultures and COS-7 cells expressing EAAT2. Additionally, both in vivo pharmacokinetic and in vitro ADME-Tox profiles confirm the favorable drug-like properties of (***R***)**-AS-1**, positioning it as the first-in-class small-molecule PAM of EAAT2, with a high prospect for further preclinical and clinical development in epilepsy and other CNS disorders [11].

Building on the promising profile of (***R***)**-AS-1**, we aimed to enhance its antiseizure efficacy by applying several structural modifications. Specifically, we proposed herein the introduction of a 4-benzyloxy-benzyl moiety in place of the benzyl fragment in (***R***)**-AS-1**. The 4-benzyloxy-benzyl fragment was proposed based on data for functionalized amino acids described by Park et al. [12]; furthermore, this moiety is also present in safinamide (SAF) chemically classified as an α-aminoamide. Notably, SAF as an inhibitor of monoamine oxidase B (MAO-B) and sodium channel blocker, exhibits both antiparkinsonian and antiseizure activity [13]. Specifically, SAF was active in the MES model in mice (ED_50_ = 4.1 mg/kg) and furthermore underwent Phase II clinical trials in epilepsy in 2008; however, these results have not been published yet [14,15]. Other 4-benzyloxy-benzyl derivatives demonstrated similar or lower antiseizure protection, as well as increased toxicity compared to SAF [12,14,16,17]. Therefore, to investigate the structure–antiseizure activity relationships (SAR) in a series of functionalized amino acid derivatives developed by our team [11,18,19], in the present study, we proposed the following structural modification of (***R***)**-AS-1** (Figure 1). In **Series 1_A**, we designed hybrid molecules by combining the parent compound (***R***)**-AS-1** with a fragment derived from SAF, specifically, (4-(benzyloxy)phenyl)methanamine. It should be emphasized that the presence of a methyl group at the alkylamide linker characteristic of parent (***R***)**-AS-1** has demonstrated significant advantages across multiple series of previously developed succinimides [11,18,19,20,21,22,23]. Moving forward to **Series 1_B**, we synthesized a dimethyl analog of the most promising compound from **Series 1_A** to address chirality-related issues by introducing a second methyl substituent at the alkylamide linker. Moreover, we hypothesized that the increased lipophilicity of the dimethyl analogs (**Series 1_B**) could potentially enhance their ability to penetrate CNS, and as a result, such modification may improve protection in seizure models. Finally, in **Series 1_C**, we aimed to synthesize analogs for one compound from **Series 1_A,** with the benzyloxy group positioned at the *ortho* and *meta* positions of the benzylamine fragment to assess the influence of constitutional isomerism on antiseizure activity. Additionally, for selected, active compounds identified in **Series 1_A-C**, we replaced the pyrrolidino-2,5-dione ring with pyrrolidin-2-one (**Series 2**) to evaluate the role of pyrrolidino-2,5-dione as a pharmacophore. This modification enabled us to approximate the structure of new compounds to levetiracetam (LEV), which is one of the newest ASMs with high therapeutic utility.

The studies described herein show a comprehensive approach to the discovery of new ASM candidates and involve design, synthesis, and extensive in vivo evaluations of antiseizure properties of the new and focused library of compounds. Additionally, the most potent anticonvulsant was subjected to in vitro mechanism of action studies, as well as safety assessment, using rotarod test in mice and standard in vitro assays, such as the evaluation of hepatotoxicity and neurotoxicity.

## 2. Results and Discussion

### 2.1. Chemistry

In the chemical studies, we synthesized a library of 23 original compounds categorized into two distinct series. It is important to note that, due to ethical issues regarding the use of animal models as the primary screening method (a common practice for the identification of new ASMs), we limited modifications of the core structure of the lead compound to groups previously identified in this study as favorable for antiseizure activity. This approach ensures that the substitution mode proposed in the current study is guided by prior research findings, optimizing the likelihood of discovering effective antiseizure agents while adhering to ethical standards [24].

The final compounds from all series were synthesized using a multistep procedure, which also included the preparation of amines **A19**–**A36** (for details, see the Supporting Information Scheme S1). Thus, all noncommercial (benzyloxy)phenyl)methanamine derivatives (**A19**–**A36**) were synthesized according to Scheme S1 in a two-step reaction. First, (benzyloxy)benzonitrile derivatives (**A1**–**A18**) were obtained using the alkylation reaction of 4-cyanophenol, 3-cyanophenol, or 2-cyanophenol with the appropriate substituted benzyl bromide in the presence of K_2_CO_3_. The nitrile group was then reduced by LiAlH_4_ (2.4 M TFH solution), yielding the desired starting amines (**A19**–**A36**), which were used for subsequent reactions without purification.

**Scheme 1:**
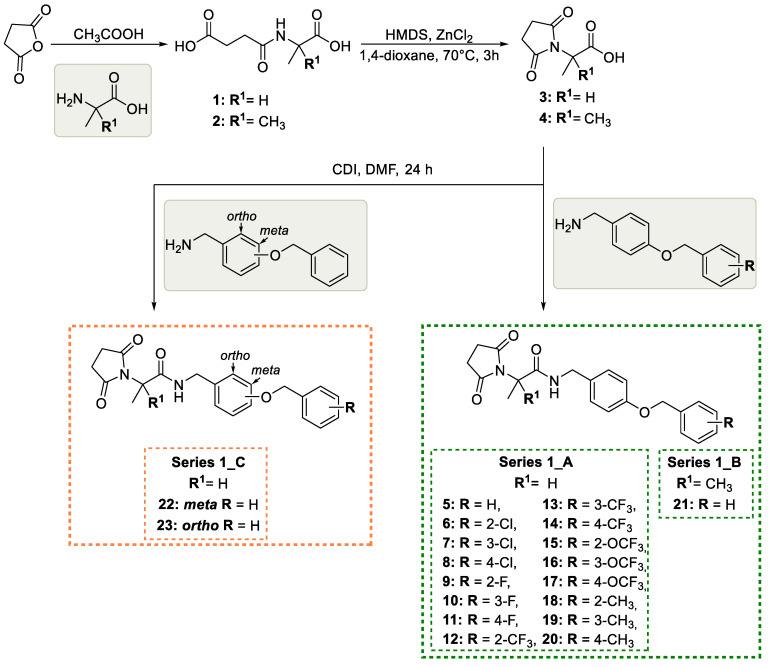
Synthesis of intermediates **1**–**4** and target compounds **5**–**23**.

The final compounds from **Series 1_A** (**5**–**20**), **Series 1_B** (**21**), and **Series 1_C** (**22**–**23**) were synthesized according to Scheme 1. Initially, succinamic acids (**1**, **2**) were synthesized by reacting equimolar amounts of commercially available succinic anhydride with DL-alanine (**Series 1_A** and **Series 1_C**) or 2-amino-2-methylpropanoic acid (**Series 1_B**). Subsequently, the hexamethyldisilazane (HMDS)-promoted cyclization of **1**, **2** was carried out according to a previously reported method, yielding intermediate monocarboxylic acids **3**, **4**. The final compounds (**5**–**23**) were obtained by coupling **3**, **4** with (4-(benzyloxy)phenyl)methanamine (**A19**–**A34**) derivatives (3-(benzyloxy)phenyl)methanamine (**A36**) or (2-(benzyloxy)phenyl)methanamine (**A35**) in the presence of carbonyldiimidazole (CDI) as the coupling reagent. This reaction was performed at room temperature in dry dimethylformamide (DMF), and its progress was monitored using high-performance liquid chromatography (HPLC), typically reaching completion within 24 h.

**Scheme 2:**
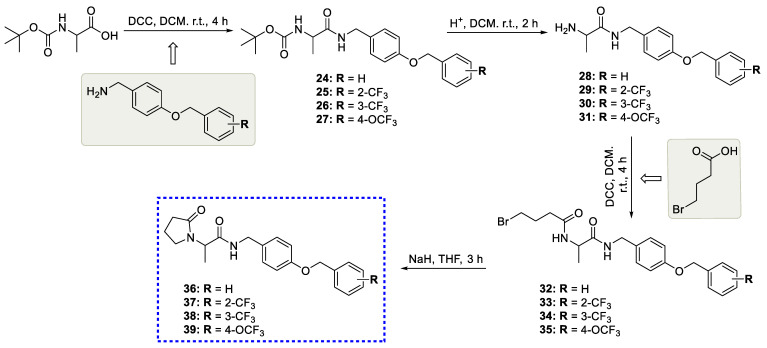
Synthesis of intermediates **24**–**35** and target pyrrolidin-2-on analogs **36**–**39**.

In the subsequent step, the pyrrolidin-2-on analogs (**Series 2**) for the selected and active pyrrolidine-2,5-diones (from **Series 1**) were synthesized following the procedure outlined in Scheme 2. First, commercially available *N*-(*tert*-butoxycarbonyl)-DL-alanine was coupled in the presence of *N*,*N*′-dicykloheksylokarbodiimid (DCC) with the appropriate (4-(benzyloxy)phenyl)methanamine (**A26, A27, A31**) to form respective amide derivatives **24**–**27**. Next, the removal of the *tert*-butyloxycarbonyl protecting group in **24**–**27** with trifluoroacetic acid (TFA), followed by neutralization with ammonium hydroxide yielded amine derivative **28**–**31**. When these amines reacted with an equimolar amount of 4-bromobutyric acid, they produced the corresponding bromobutyric amides **32**–**35**. Next, compounds **32**–**35** were cyclized in the presence of sodium hydride to give the desiderate products **36**–**39**.

The target compounds were obtained in good yield (>70%). The structures of selected intermediates and all final molecules were confirmed using ^1^H NMR, ^13^C NMR, and LC-MS spectra analyses. The purity of the final compounds, determined using the UPLC method, was >97%. Moreover, for all final compounds, high-resolution mass spectrometry (HRMS) analyses were carried out. An elemental analysis (C, H, and N) was performed for all final compounds synthesized (for details, see the Section 3).

### 2.2. In Silico Studies

The physicochemical properties of all final compounds were evaluated using Lipinski’s and Veber’s rules via the SwissAdme online tool (Table 1). These rules are fundamental for assessing the drug-like properties of compounds, particularly their suitability for oral absorption in humans. Lipinski’s criteria include a molecular weight (MW) of ≤500 Da, a log P of ≤5, hydrogen bond donors (HBD) ≤ 5, and ≤10 hydrogen bond acceptors (HBA). Veber’s criteria focus on ≤10 rotatable bonds (NBR) and a topological polar surface area (TPSA) of ≤140 Å^2^. According to the data in Table 1, all synthesized compounds conform to these criteria.

Furthermore, the compounds were assessed for their central nervous system (CNS) multiparameter optimization (MPO) scores using Instant JChem by ChemAxon software version 21.4.0. The CNS MPO score is an established algorithm that considers the following six key physicochemical properties: calculated logP (ClogP), calculated distribution coefficient at pH 7.4 (ClogD), TPSA, HBD, MW, and the most basic center (pKa). Each property is scored between zero and one, leading to a maximum cumulative score of six. Higher CNS MPO scores are desirable, with a score ≥4.0 commonly used as a threshold in CNS drug discovery. Notably, all final compounds achieved scores greater than 4.5, indicating their strong potential as CNS-active agents. This thorough analysis of physicochemical properties and CNS MPO scores suggests that the synthesized compounds are not only drug-like according to traditional rules but also optimized for CNS activity, making them promising candidates for further pharmacological investigation.

### 2.3. Anticonvulsant Activity

Epilepsy is a multifaceted disorder with often unclear etiology, making it challenging to pinpoint a specific receptor or transporter as the definitive target [25]. Consequently, the discovery of ASMs deviates from the traditional drug development pathway, which typically involves designing a new molecule, docking it with a specific biological target, and performing chemical synthesis, followed by in vitro assays. Instead, numerous animal seizure models, which mirror various types of human epileptic seizures, are initially employed in the process of new ASM discovery [26]. This approach has been instrumental in the identification and introduction into clinical practice of all currently used ASMs and continues to be a cornerstone in the preclinical development of new anticonvulsants [26]. The primary justification for prioritizing in vivo screening over in vitro binding and functional studies is the complex and often not fully elucidated mechanisms of action of known ASMs. This approach also facilitates the discovery of compounds with novel, previously undefined pharmacodynamic properties. In light of this, we evaluated all compounds obtained in the current studies using the MES test, which remains one of the most valuable preclinical screening models for tonic-clonic seizures, as well as partial convulsions with or without secondary generalization in humans. This test allows for a comprehensive assessment of the anticonvulsant efficacy of new ASM candidates and is considered highly relevant for predicting their therapeutic efficacy in human epilepsy [27]. The MES test was carried out in mice following the intraperitoneal (i.p.) administration of a screening dose of 100 mg/kg in a group consisting of four animals. The efficacy of the compounds synthesized was evaluated at 0.5 h pretreatment time. The screening data are summarized in Table S1.

In the MES screening, compounds **5**, **6**, **9**, **10**, **12**, **13**, **16**, **17**, and **36**–**39** exhibited the highest level of antiseizure protection, achieving 100% efficacy (protection observed in all four mice tested). Compound **18** also demonstrated robust protection, with a 75% efficacy rate (protecting three out of four mice tested), whereas **8**, **14**, and **15** showed 50% protection (two out of four mice). Weaker activity was observed for **7**, **11**, **19**, **20**, and **21**–**23**, which displayed limited 25% protection or were ineffective in the MES test. 

These findings suggest that the most potent antiseizure activity was observed in **Series 1_A** and **Series 2** for unsubstituent derivatives **5** and **36**, as well as compounds that feature an electron-withdrawing atom or group at the 2-position of the benzyloxy moiety, specifically, **6** (2-F), **9** (2-Cl), **12** (2-CF_3_), and **37** (2-CF_3_), and at the 3-position, specifically, **10** (3-Cl), **13** (3-CF_3_), **16** (3-OCF_3_), and **38** (3-OCF_3_). Interestingly, in the case of substituents at the 4-position, the most active compounds were those with a methoxy group, particularly compounds **17** and **39**. Additionally, replacing the benzyloxy group at the *para* position to the *meta* **22** and *ortho* **23** positions (**Series 1_C**) resulted in a decrease in antiseizure activity. Furthermore, the introduction of a methyl group in the alanine fragment **21** (**Series 1_B**) also caused a loss of antiseizure protection.

In the subsequent phase of pharmacological studies, all compounds, **5**–**23** and **36**–**39,** were subjected to testing using the 6 Hz (32 mA) seizure model. This model is a well-established animal model of human focal seizures [28]. The screening results are shown in Table S1. In this model, maximal (100% efficacy) was demonstrated by compounds **6**, **7**, **13**, and **39**, and weaker, but satisfying, activity was provided by compounds **9**, **10, 12**, **17**, **18**, and **38** (75% protection), whereas compounds **5**, **8**, **14**, **16**, and **37** showed 50% protection. Limited activity (25% protection) was observed for **11**, **15**, **19**, and **36**. Importantly, only four compounds demonstrated a lack of protective effects in the 6 Hz (32 mA) seizure model. Based on the screening results, it can be concluded that the compounds obtained herein demonstrated similar efficacy in the MES, as well as in the 6 Hz (32 mA) seizure model.

In the next step of pharmacological characterization, the median effective doses (ED_50_) were determined for selected compounds showing a minimum of 75% protection at doses of 100 mg/kg in both MES and 6 Hz (32 mA) tests. Furthermore, the CNS safety profile (influence on motor coordination) of these compounds was evaluated by establishing the median toxic doses (TD_50_) using the rotarod test 0.5 h post i.p. administration. The protective indexes (PIs) were then calculated as the ratio of TD_50_ to ED_50_ (PI = TD_50_/ED_50_), providing an assessment of the therapeutic window for each compound. The obtained results, as well as previously published data for standard ASMs with established clinical utility, such as levetiracetam (LEV, effective in the 6 Hz test [32 mA]), lacosamide (LCS, active in the MES and 6 Hz [32/44 mA] tests), and valproic acid (VPA), which is widely recognized as a broad-spectrum ASM (effective in the MES, 6 Hz [32/44 mA]), and chemical prototype (***R***)**-AS-1**, are summarized in Table 2. 

The results obtained revealed that all compounds provided significant protection against seizures in the MES and 6 Hz (32 mA) models. The pyrrolidin-2,5-dione analogs, particularly compound **5**, demonstrated a promising pharmacological profile, balancing efficacy with ED_50_ values of 48.00 mg/kg in the MES model, 45.19 mg/kg in the 6 Hz model, and safety TD_50_ > 300 mg/kg in the rotarod test. Compounds **12** and **17** also exhibited significant anticonvulsant activity and safety margins, with **17** showing the lowest ED_50_ in the MES model with slightly weaker activity in the 6 Hz (32 mA). Interestingly, the pyrrolidin-2-one analogs, **38** and **39**, also displayed broad anticonvulsant activity. Nevertheless, compound **39**, in particular, showed weaker performance in the MES test compared to its pyrrolidin-2,5-dione counterpart **17**. Unfortunately, compound **39** exhibited a lower safety margin (TD_50_ < 300 mg/kg). This could suggest that the exchange of pyrrolidin-2,5-dione to the pyrrolidin-2-one ring significantly impacts their safety profiles.

When comparing these new compounds to standard ASMs (LCS, LEV, VPA), several pyrrolidin-2,5-dione analogs, such as **5**, **12**, **16**, and **17**, demonstrated similar or superior efficacy and safety profiles. For instance, all compounds obtained exhibited better activity in MES and 6 Hz (32 mA) models and higher PIs than VPA, which is still recognized as the most frequently prescribed first-line ASD in different types of epilepsies [31]. Unfortunately, none of the compounds exhibited potency superior to that of LCS in any models, particularly LEV, which is widely recognized as a benchmark ASM due to its efficacy in psychomotor 6 Hz (32 mA) seizures and its outstanding safety profile. However, it is noteworthy that LEV was not active in the MES model. When evaluating the activity of the newly synthesized compounds against the chemical prototype (***R***)**-AS-1**, several notable differences in pharmacological properties are observed. (***R***)**-AS-1** demonstrates a higher therapeutic index in both the MES and 6 Hz models, indicating a superior safety profile (nevertheless this may result from a lower dosing range tested for compounds described here in the rotarod). Particularly interesting is compound **17**, which exhibits higher potency in the MES model vs. (***R***)**-AS-1**. Similarly, compounds **5**, **16**, and **39** show greater activity in the MES model compared to (***R***)**-AS-1**, but simultaneously, their protection in the 6 Hz model was weaker, suggesting a less favorable safety profile in this model. In summary, while some of the new compounds, particularly compound **17**, demonstrate potent antiseizure activity in the MES model, their protective indexes are generally lower compared to the prototype (***R***)**-AS-1**, especially in the 6 Hz model. Additionally, compound **5** deserves special attention, as it demonstrates balanced effective dose values, with similar efficacy in both the MES and 6 Hz models. This indicates a consistent pharmacological profile across different seizure models.

Given the satisfying protection of compound **5** observed in the 6 Hz (32 mA) seizures, it was further evaluated using a higher current intensity of 44 mA in the same model (Table 3). 

It is important to note that the 6 Hz (44 mA) seizure model is extensively used to identify substances with potential efficacy in DRE [33,34]. Despite the reduced protective efficacy of compound **5**, it was similarly active compared to VPA, a broad-spectrum ASM with a multitarget mechanism of action. It is significant to highlight that in this seizure model, LEV, which targets the SV2A protein in synaptic vesicle membranes, was ineffective, even at a high dose of 500 mg/kg. However, it should be noted that compound **5** was not more effective than LCS and was also less active compared to the chemical prototype (***R***)**-AS-1** in this model. This underscores the need for further structural optimization to enhance antiseizure efficacy in the 6 Hz (44 mA) test relative to the benchmark compounds. 

In summary, the obtained in vivo results enabled us to identify compound **5** as the lead compound that is characterized by potent and broad-spectrum anticonvulsant activity, no induction of motor impairment in the rotarod test, and a very favorable therapeutic window, as described by PI values. This hybrid molecule provides a greater potency and/or an improved safety profile compared to reference VPA when tested under the same conditions. However, it is important to highlight that compound **5** was less active than the chemical prototype (***R***)**-AS-1** in both the 6 Hz (32 mA) and 6 Hz (44 mA) seizure models. In the MES model, both compounds revealed relatively similar protection (ED_50_s of 48.0 and 66.3 mg/kg, respectively); nevertheless, it should be taken into account that these values were derived from separate experiments, which may affect direct comparison. Collectively, data obtained herein justify further and more comprehensive chemical and pharmacological studies among structurally related derivatives in the future.

### 2.4. In Vitro Tox Assays

**Figure 2:**
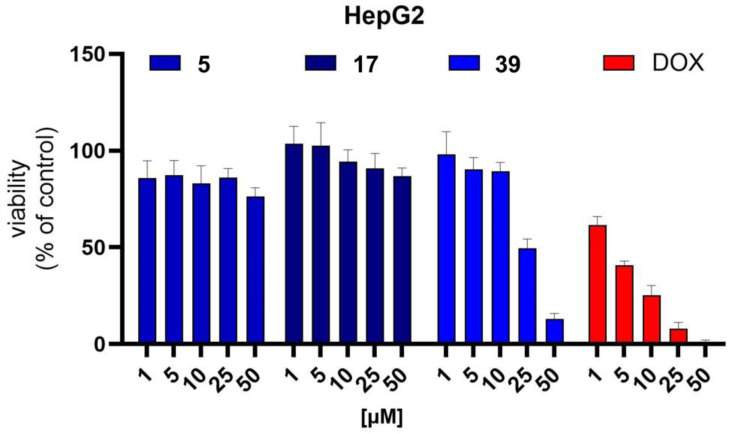
The viability of HepG2 cells incubated in the presence of **5**, **17**, and **39**. HepG2 cells were exposed to growing concentrations (1–50 µM) of tested compounds for 24 h. Cell viability was measured using an MTT assay. Bars represent mean percent of cell viability normalized to non-treated cells (100%) ± SEM. The doxorubicin was tested as a positive control.

**Figure 3:**
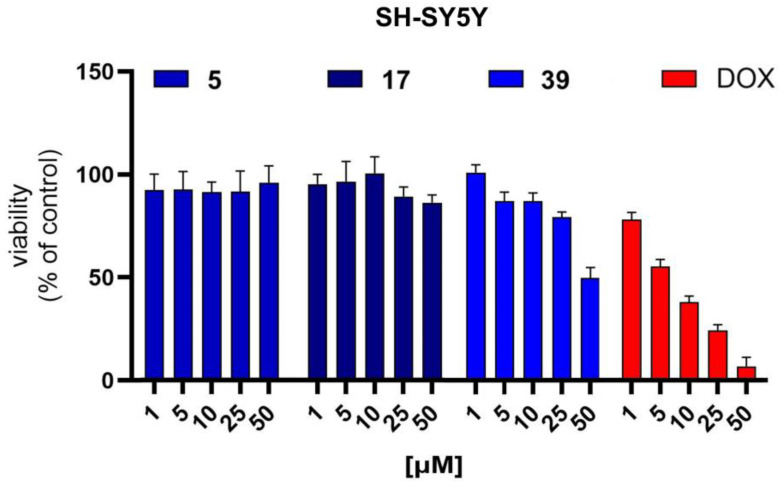
The viability of SH-SY5Y cells incubated in the presence of compounds **5**, **17**, and **39**. SH-SY5Y cells were exposed to growing concentrations (1–50 µM) of tested compounds for 24 h. Cell viability was measured using an MTT assay. Bars represent mean percent of cells viability normalized to non-treated cells (100%) ± SEM. The doxorubicin was tested as a positive control.

Cytotoxicity is a significant factor leading to the failure of compounds during various stages of drug development. Additionally, hepatotoxicity data on certain ASMs, such as VPA, phenytoin, and felbamate [35,36], provided a basis for evaluating the potential hepatotoxicity of new ASM candidates. The objective of developing new antiepileptic therapies is to maximize efficacy while minimizing toxicity. Ideally, new ASMs should exhibit high efficacy with minimal or no hepatic metabolism. Consequently, preliminary in vitro hepatocytotoxicity and neurocytotoxicity assessments were conducted using commercially available cell lines, human hepatocellular carcinoma cell line (HepG2) and human neuroblastoma cell line (SH-SY5Y), respectively. The results indicate that the tested pyrrolidine-2,5-dione derivatives **5** and **17** do not exhibit toxic effects on human hepatocellular carcinoma cells or neuroblastoma cells (Figure 2 and Figure 3). Specifically, cell viability in the presence of compounds **5** and **17** at a concentration of 10 µM was significantly higher compared to cells exposed to the positive control, the known chemotherapeutic agent doxorubicin. However, it was observed that the pyrrolidone-2-one derivative **39** caused a statistically significant decrease in cell viability at a higher concentration of 50 µM, indicating potential cytotoxic effects at elevated doses. It should be emphasized that the lead compound **5** also confirmed a high safety profile in the in vitro hepatotoxicity and neurotoxicity assays.

### 2.5. In Vitro Radioligand Binding Studies and Functional Assays

Despite the availability of numerous sophisticated in vitro assays (binding, functional, biochemical, etc.) that facilitate the elucidation of mechanisms of action for drug candidates, the discovery and development of new ASMs still predominantly rely on established animal seizure models as the primary step in the discovery process. It is worth mentioning that the precise mechanisms of action for many ASMs currently employed in clinical treatment (e.g., LEV or LCS) were fully elucidated only after their widespread therapeutic application [37].

Sodium and calcium channels are universally recognized as crucial and indispensable molecular targets for a variety of structurally diverse ASMs, including LCS, lamotrigine, carbamazepine, and oxcarbazepine, among others [38]. In light of this, the binding profile of the most attractive compound identified in in vivo studies, specifically lead molecule **5**, was evaluated in vitro and involved, among others, its interaction with the sodium channel (site 2) and calcium Cav_1_._2_ channel (L-type) at a concentration of 100 μM (Table 4). It is critical to highlight that numerous neurobiological investigations in recent years have shown that the dysfunction of Cav_1_._2_ calcium channels may contribute to the pathogenesis of epilepsy and neuropathic pain [39,40].

The results obtained proved that compound **5**, at a concentration of 100 µM, exhibits significant inhibition of the Cav_1_._2_ (L-type) calcium channel, with an inhibition rate exceeding 50%. This suggests that antagonism of the Cav_1_._2_ (L-type) calcium channels could be integral to the anticonvulsant properties of the compound. Notably, voltage-gated Cav_1_._2_ (L-type) channels are ubiquitously expressed in the central nervous system (CNS) and play a critical role in regulating neuronal firing [39]. Therefore, antagonists of these channels are promising candidates for anticonvulsant and antinociceptive therapies [39,40,41]. However, it remains to be confirmed in the pharmacokinetic studies whether a concentration of 100 µM can be achieved in the brain, taking into consideration the free fraction of the compound in the brain tissue, as well. Further investigations are certainly necessary for reliable PK/PD analysis. Compound **5** was also evaluated for interaction with other ion channels, receptors, and transporters commonly associated with anticonvulsant activity, including the Cav_2_._2_ (N-type) calcium channel, GABA transporters, and glycine transporters (GlyT1) at a concentration of 100 μM. Results indicated no significant interaction with these targets. Notably, at a concentration of 100 μM, compound **5** did not bind to the potassium (hERG) channel, a key off-target known for its potential to induce pro-arrhythmic effects through QT interval prolongation. Therefore, it can be concluded that compound **5** possesses a low risk of inducing harmful pro-arrhythmic activity. 

Additionally, due to the structural similarity of the obtained hybrid compounds to SAF, which includes a benzyloxy moiety and is known to inhibit monoamine oxidase B (MAO-B), inhibition assays of this enzyme were conducted for all synthesized compounds. However, none of the compounds demonstrated inhibitory activity against MAO-B at concentration, unlike SAF (IC_50_) and rasagiline (IC_50_), which were used as positive controls. The detailed data are provided in the Supplementary Table S2.

## 3. Materials and Methods

### 3.1. Chemistry

All chemicals and solvents were purchased from commercial suppliers and were used without further purification. Melting points (mp.) were determined in open capillaries on a Büchi B-540 melting point apparatus (Büchi Labortechnik, Flawil, Switzerland). TLC and gradient UPLC chromatography were used to assess the purity and homogeneity of the compounds. TLC was carried out on silica gel 60 F_254_ pre-coated aluminum sheets (Macherey-Nagel, Düren, Germany), using the following developing systems: S_1_–DCM:MeOH (9:0.3; *v*/*v*), S_2_–DCM:MeOH (9:0.5; *v*/*v*). Spots detection: UV light (λ = 254 nm). The UPLC and mass spectra (LC-MS) were obtained using the Waters ACQUITY™ TQD system (Waters, Milford, CT, USA) with the MS-TQ and UV–Vis-DAD eλ detectors. The ACQUITY UPLC BEH C18, 1.7 μm (2.1 × 100 mm) column was used with the VanGuard Acquity UPLC BEH C18, 1.7 μm (2.1 × 5 mm) (Waters, Milford, CT, USA). Standard solutions (1 mg/mL) of each compound were prepared in analytical grade MeCN/water mixture (1:1; *v*/*v*). Conditions applied were as follows: eluent A (water/0.1% HCOOH), eluent B (MeCN/0.1% HCOOH), a flow rate of 0.3 mL/min, a gradient of 5–100% B over 10 min, and an injection volume of 10 μL. The UPLC analyses and high-resolution mass spectra (LC-HRMS) were obtained on a Waters ACQUITY I-Class PLUS SYNAPT XS High-Resolution Mass Spectrometer (Waters, Milford, CT, USA) with an MS-Q-TOF detector and a UV–vis-DAD eλ detector. The UPLC retention times (*t*_R_) are given in minutes. The purity of target compounds determined using the chromatographic UPLC method was ≥97%. Preparative column chromatography was performed using silica gel 60 (particle size 0.063–0.200; 70–230 Mesh ATM) purchased from Merck (Darmstadt, Germany). Elemental analyses (C, H, and N) for final compounds were carried out by a micro method using the elemental Vario EI III Elemental analyzer (Hanau, Germany). The results of elemental analyses were within ±0.4% of the theoretical values. ^1^H NMR and ^13^C NMR spectra were obtained using a JEOL-500 spectrometer (JEOL USA, Inc., Peabod, MA, USA) in CDCl_3_ operating at 500 MHz (^1^H NMR) and 126 MHz (^13^C NMR) or using a Varian Mercury spectrometer (Varian Inc., Palo Alto, CA, USA) operating at 300 MHz (^1^H NMR), 75 MHz (^13^C NMR). Chemical shifts are reported in δ values (ppm) relative to TMS δ = 0 (^1^H) as an internal standard. The *J* values are expressed in Hertz (Hz). Signal multiplicities are represented by the following abbreviations: s (singlet), br s (broad singlet), d (doublet), dd (double doublet), ddd (double double doublet), t (triplet), td (triplet of doublets), q (quartet), and m (multiplet). 

#### 3.1.1. Synthetic Procedure for Amidoacids **1**–**2**

Succinic anhydride (6.0 g, 60 mmol, 1 eq) was dissolved in 15 mL of concentrated acetic acid, followed by the addition of appropriate amino acid (60 mmol, 1 eq). The mixture was heated to 70 °C and stirred at this temperature for 12 h. After this period, the acetic acid was evaporated to dryness. The amidoacids **1**–**2** were obtained as a white solid after washing with diethyl ether.

4-((1-carboxyethyl)amino)-4-oxobutanoic acid (**1**) White solid. Yield: 85% (9.64 g); UPLC (purity > 99.99%): *t*_R_ = 1.63 min. C_7_H_11_NO_5_ (189.17). LC-MS (ESI): *m*/*z* calcd for C_7_H_11_NO_5_ (M − H)^+^ 188.06, found 188.2.
4-((2-carboxypropan-2-yl)amino)-4-oxobutanoic acid (**2**) White solid. Yield: 79% (9.52 g); UPLC (purity 99.86%): *t*_R_ = 1.52 min. C_8_H_13_NO_5_ (203.19). LC-MS (ESI): *m*/*z* calcd for C_8_H_13_NO_5_ (M − H)^+^ 202.08, found 202.1.

#### 3.1.2. Synthetic Procedure for Succinamic Acids **3**–**4**

To a suspension of amidoacids **1**–**2** (45 mmol, 1 eq) in 1,4-dioxane (150 mL), ZnCl_2_ (6.13 g, 45 mmol, 1 eq) was added and the mixture was heated to 70 °C. Subsequently, a solution of hexamethyldisilazane (HMDS) (10.89 g, 67.5 mmol 1.5 eq) in 1,4-dioxane (50 mL) was added dropwise over 30 min. The reaction mixture was then heated for an additional 12 h and subsequently concentrated under reduced pressure. The residue was dissolved in DCM and extracted with 0.1 M of HCl (3 × 100 mL), water (3 × 100 mL), and brine (3 × 100 mL). The organic layer was then dried over anhydrous Na_2_SO_4_, and the solvent was evaporated. The product was obtained as a solid after washing with diethyl ether.

2-(2,5-dioxopyrrolidin-1-yl)propanoic acid (**3**) White solid. Yield: 95% (7.31 g); UPLC (purity > 99.99%): *t*_R_ = 1.71 min. C_7_H_9_NO_4_ (171.15). LC-MS (ESI): *m*/*z* calcd for C_7_H_9_NO_4_ (M + H)^+^ 172.05, found 172.1.
2-(2,5-dioxopyrrolidin-1-yl)-2-methylpropanoic acid (**4**) White solid. Yield: 89% (7.41 g); UPLC (purity 99.54%): *t*_R_ = 1.89 min. C_8_H_11_NO_4_ (185.18). LC-MS (ESI): *m*/*z* calcd for C_8_H_11_NO_4_ (M + H)^+^ 186.07, found 186.2.

#### 3.1.3. General Method for the Preparation of the Final Compounds **5**–**23**

Carbonyldiimidazole (0.39 g, 2.4 mmol, 1.2 eq) was dissolved in 10 mL of DMF. Afterward, this solution was added to the appropriate acid, **3** or **4** (2 mmol, 1 eq), dissolved in 10 mL of DMF (while stirring). After 0.5 h, the appropriate amine (2 mmol, 1 eq) dissolved in 5 mL of DMF was added in drops. The mixture was stirred for approximately 24 h at room temperature and evaporated to dryness. Column chromatography was applied for the purification of crude products using the developing system S_2_. The desired compounds were obtained as white powder followed by concentration of organic solvents under reduced pressure and crystallization from diethyl ether.

N-(4-(benzyloxy)benzyl)-2-(2,5-dioxopyrrolidin-1-yl)propanamide (**5**) White solid. Yield: 81% (0.59 g); mp. 102.9–103.4 °C; TLC: R_f_ **=** 0.39 (S_2_); UPLC (purity > 99.99%): *t*_R_ = 6.76 min. LC-MS (ESI): *m*/*z* calcd for C_21_H_22_N_2_O_4_ (M + H)^+^ 367.16, found 367.2. UPLC/HRMS (purity > 99.99%): *t*_R_ = 6.29 min. HRMS (ESI-QTOF): *m*/*z* calcd for C_21_H_22_N_2_O_4_Na (M + Na)^+^ 389.1477, found 389.1456. ^1^H NMR (500 MHz, CDCl_3_) δ 1.56 (d, *J* = 7.3 Hz, 3 H), 2.69 (s, 4 H), 4.34 (d, *J* = 5.6 Hz, 2 H), 4.76 (q, *J* = 7.3 Hz, 1 H), 5.03 (s, 2 H), 6.24 (br s, 1 H), 6.91–6.93 (m, 2 H), 7.17 (m, *J* = 8.6 Hz, 2 H), 7.31–7.38 (m, 3 H), 7.39–7.42 (m, 2 H); ^13^C NMR (126 MHz, CDCl_3_) δ 14.5, 28.3, 43.4, 49.8, 70.1, 115.1, 127.5, 128.1, 128.7, 129.2, 130.2, 137.0, 158.3, 168.5, 176.9. Anal. calcd for C_21_H_22_N_2_O_4_ (366.42): C: 68.84, H: 6.05, N: 7.65; Found C: 68.71, H: 6.10, N: 7.70.
2-(2,5-dioxopyrrolidin-1-yl)-*N*-(4-((2-fluorobenzyl)oxy)benzyl)propanamide (**6**) White solid. Yield: 85% (0.65 g); mp. 129.5–130.1 °C; TLC: R_f_ = 0.42 (S_2_); UPLC (purity 98.53%): *t*_R_ = 6.80 min, LC-MS (ESI): *m*/*z* calcd for C_21_H_21_FN_2_O_4_ (M + H)^+^ 385.15, found 385.2. UPLC/HRMS (purity 98.48%): *t*_R_ = 6.34 min. HRMS (ESI-QTOF): *m*/*z* calcd for C_21_H_21_FN_2_O_4_Na (M + Na)^+^ 407.1383, found 407.1388. ^1^H NMR (500 MHz, CDCl_3_) δ 1.57 (d, *J* = 7.3 Hz, 3 H), 2.70 (s, 4 H), 4.36 (d, *J* = 5.6 Hz, 2 H), 4.77 (q, *J* = 7.3 Hz, 1 H), 5.10 (s, 2 H), 6.22 (br s, 1 H), 6.93 (d, *J* = 8.6 Hz, 2 H), 7.07 (ddd, *J* = 9.8, 8.5, 0.9 Hz, 1 H), 7.14 (td, *J* = 7.5, 1.0 Hz, 1 H), 7.18 (d, *J* = 8.7 Hz, 2 H), 7.27–7.33 (m, 1 H), 7.45–7.50 (m, 1 H); ^13^C NMR (126 MHz, CDCl_3_) δ 14.5, 28.3, 43.4, 49.8, 63.8, 63.8, 115.1, 115.4, 115.5, 124.1 (d, *J* = 14.2 Hz), 124.4 (d, *J* = 3.7 Hz), 129.2, 129.8, 129.9, 130.4, 158.1, 159.4, 160.5 (d, *J* = 246.8 Hz), 161.6, 168.5, 176.9. Anal. calcd for C_21_H_21_FN_2_O_4_ (384.41): C: 65.62, H: 5.51, N: 7.29; Found C: 65.71, H: 5.31, N: 7.38.
2-(2,5-dioxopyrrolidin-1-yl)-*N*-(4-((3-fluorobenzyl)oxy)benzyl)propanamide (**7**) White solid. Yield: 87% (0.66 g); mp. 131.4–132.1 °C; TLC: R_f_ = 0.42 (S_2_); UPLC (purity 98.97%): *t*_R_ = 6.81 min, LC-MS (ESI): *m*/*z* calcd for C_21_H_21_FN_2_O_4_ (M + H)^+^ 385.15, found 385.2. UPLC/HRMS (purity 98.94%): *t*_R_ = 6.27 min. HRMS (ESI-QTOF): *m*/*z* calcd for C_21_H_21_FN_2_O_4_ (M + H)^+^ 385.1519, found 385.1533. ^1^H NMR (500 MHz, CDCl_3_) δ 1.58 (d, *J* = 7.3 Hz, 4 H), 2.73 (s, 4 H), 4.38 (d, *J* = 5.6 Hz, 2 H), 4.79 (q, *J* = 7.3 Hz, 1 H), 5.04 (s, 2 H), 6.14 (br s, 1 H), 6.91 (d, *J* = 8.6 Hz, 2 H), 7.00 (td, *J* = 8.4, 2.6 Hz, 1 H), 7.12–7.21 (m, 4 H), 7.33 (td, *J* = 7.9, 5.8 Hz, 1 H); ^13^C NMR (126 MHz, CDCl_3_) δ 14.6, 28.3, 43.5, 49.9, 69.3, 69.3, 114.3 (d, *J* = 22.0 Hz), 114.9 (d, *J* = 21.1 Hz), 115.2, 122.7, 122.8, 129.3, 130.2, 130.3, 130.4, 139.6, 139.6, 158.1, 163.1 (d, *J* = 246.4 Hz), 168.4, 176.8. Anal. calcd for C_21_H_21_FN_2_O_4_ (384.41): C: 65.62, H: 5.51, N: 7.29; Found C: 65.56, H: 5.60, N: 7.34.
2-(2,5-dioxopyrrolidin-1-yl)-*N*-(4-((4-fluorobenzyl)oxy)benzyl)propanamide (**8**) White solid. Yield: 79% (0.60 g); mp. 127.4–127.9 °C; TLC: R_f_ = 0.42 (S_2_); UPLC (purity 98.65%): *t*_R_ = 6.80 min, LC-MS (ESI): *m*/*z* calcd for C_21_H_21_FN_2_O_4_ (M + H)^+^ 385.15, found 385.2. UPLC/HRMS (purity 98.60%): *t*_R_ = 6.38 min. HRMS (ESI-QTOF): *m*/*z* calcd for C_21_H_21_FN_2_O_4_Na (M + Na)^+^ 407.1383, found 407.1388. ^1^H NMR (500 ^1^H NMR (500 MHz, CDCl_3_) δ 1.57 (d, *J* = 7.3 Hz, 3 H), 2.70 (s, 4 H), 4.36 (d, *J* = 5.6 Hz, 2 H), 4.77 (q, *J* = 7.3 Hz, 1 H), 4.99 (s, 2 H), 6.23 (br s, 1 H), 6.90 (d, *J* = 8.7 Hz, 3 H), 7.05 (t, *J* = 8.7 Hz, 2 H), 7.18 (d, *J* = 8.6 Hz, 3 H), 7.38 (dd, *J* = 8.7, 5.4 Hz, 2 H); ^13^C NMR (126 MHz, CDCl_3_) δ 14.5, 28.3, 43.4, 49.8, 69.4, 115.1, 115.5, 115.7, 129.2, 129.4, 129.4, 130.4, 132.7, 132.7, 158.2, 162.5 (d, *J* = 246.2 Hz), 161.6, 163.6, 168.5, 176.9. Anal. calcd for C_21_H_21_FN_2_O_4_ (384.41): C: 65.62, H: 5.51, N: 7.29; Found C: 65.58, H: 5.47, N: 7.26.
*N*-(4-((2-chlorobenzyl)oxy)benzyl)-2-(2,5-dioxopyrrolidin-1-yl)propanamide (**9**) White solid. Yield: 78% (0.62 g); mp. 112.1–112.8 °C; TLC: R_f_ = 0.44 (S_2_); UPLC (purity 99.36%): *t*_R_ = 7.40 min, LC-MS (ESI): *m*/*z* calcd for C_21_H_21_ClN_2_O_4_ (M + H)^+^ 401.12, found 401.2 UPLC/HRMS (purity 99.31%): *t*_R_ = 6.84 min. HRMS (ESI-QTOF): *m*/*z* calcd for C_21_H_21_ClN_2_O_4_ (M + H)^+^ 401.1223, found 401.1235. ^1^H NMR (500 MHz, CDCl_3_) δ 1.57 (d, *J* = 7.3 Hz, 3 H), 2.71 (s, 4 H), 4.36 (d, *J* = 5.6 Hz, 2 H), 4.77 (q, *J* = 7.4 Hz, 1 H), 5.01 (s, 2 H), 6.21 (br s, 1 H), 6.90 (d, *J* = 8.7 Hz, 2 H), 7.18 (d, *J* = 8.6 Hz, 2 H), 7.26–7.32 (m, 3 H), 7.38–7.43 (m, 1 H); ^13^C NMR (126 MHz, CDCl_3_) δ 14.5, 28.3, 43.4, 49.8, 53.5, 69.2, 115.1, 125.4, 127.5, 128.2, 129.2, 130.0, 130.5, 134.6, 139.1, 158.0, 168.5, 176.9. Anal. calcd for C_21_H_21_ClN_2_O_4_ (400.86): C: 62.92, H: 5.28, N: 6.99; Found C: 62.85, H: 5.29, N: 6.92.
*N*-(4-((3-chlorobenzyl)oxy)benzyl)-2-(2,5-dioxopyrrolidin-1-yl)propanamide (**10**) White solid. Yield: 82% (0.65 g); mp. 100.2–101.3 °C; TLC: R_f_ = 0.44 (S_2_); UPLC (purity 97.21%): *t*_R_ = 7.41 min, LC-MS (ESI): *m*/*z* calcd for C_21_H_21_ClN_2_O_4_ (M + H)^+^ 401.12, found 401.2 UPLC/HRMS (purity 97.56%): *t*_R_ = 6.87 min. HRMS (ESI-QTOF): *m*/*z* calcd for C_21_H_21_ClN_2_O_4_Na (M + Na)^+^ 423.1088, found 423.1104. ^1^H NMR (500 MHz, CDCl_3_) δ 1.57 (d, *J* = 7.3 Hz, 3 H), 2.71 (s, 4 H), 4.36 (d, *J* = 5.6 Hz, 2 H), 4.77 (q, *J* = 7.4 Hz, 1 H), 5.01 (s, 2 H), 6.21 (br s, 1 H), 6.90 (d, *J* = 8.7 Hz, 2 H), 7.18 (d, *J* = 8.6 Hz, 2 H), 7.26–7.30 (m, 3 H), 7.39–7.42 (m, 1 H), 7.41 (s, 1 H); ^13^C NMR (126 MHz, CDCl_3_) δ 14.5, 28.3, 43.4, 49.8, 53.5, 69.2, 115.1, 125.4, 127.5, 128.2, 129.2, 130.0, 130.5, 134.6, 139.1, 158.0, 168.5, 176.9. Anal. calcd for C_21_H_21_ClN_2_O_4_ (400.86): C: 62.92, H: 5.28, N: 6.99; Found C: 62.83, H: 5.34, N: 6.89.
*N*-(4-((4-chlorobenzyl)oxy)benzyl)-2-(2,5-dioxopyrrolidin-1-yl)propanamide (**11**) White solid. Yield: 84% (0.66 g); mp. 145.9–146.4 °C; TLC: R_f_ = 0.44 (S_2_); UPLC (purity 99.12%): *t*_R_ = 7.37 min, LC-MS (ESI): *m*/*z* calcd for C_21_H_21_ClN_2_O_4_ (M + H)^+^ 401.12, found 401.2. UPLC/HRMS (purity 99.42%): *t*_R_ = 6.89 min. HRMS (ESI-QTOF): *m*/*z* calcd for C_21_H_21_ClN_2_O_4_Na (M + Na)^+^ 423.1088, found 423.1104. ^1^H NMR (500 MHz, CDCl_3_) δ 1.57 (d, *J* = 7.3 Hz, 3 H), 2.70 (s, 4 H), 4.35 (d, *J* = 5.7 Hz, 2 H), 4.77 (q, *J* = 7.3 Hz, 1 H), 4.99 (s, 2 H), 6.23 (br s, 1 H), 6.89 (d, *J* = 8.7 Hz, 2 H), 7.17 (d, *J* = 8.9 Hz, 2 H), 7.33 (s, 4 H); ^13^C NMR (126 MHz, CDCl_3_) δ 14.5, 28.3, 43.4, 49.8, 69.3, 115.1, 128.8, 128.9, 129.2, 130.4, 133.8, 135.5, 158.1, 168.5, 176.9. Anal. calcd for C_21_H_21_ClN_2_O_4_ (400.86): C: 62.92, H: 5.28, N: 6.99; Found C: 62.95, H: 5.21, N: 6.89.
2-(2,5-dioxopyrrolidin-1-yl)-*N*-(4-((2-(trifluoromethyl)benzyl)oxy)benzyl)propanamide (**12**) White solid. Yield: 78% (0.67 g); mp. 122.9–123.4 °C; TLC: R_f_ = 0.48 (S_2_); UPLC (purity 98.70%): *t*_R_ = 7.49 min, LC-MS (ESI): *m*/*z* calcd for C_22_H_21_F_3_N_2_O_4_ (M + H)^+^ 435.15, found 435.3 UPLC/HRMS (purity 98.90%): *t*_R_ = 6.97 min. HRMS (ESI-QTOF): *m*/*z* calcd for C_22_H_21_F_3_N_2_O_4_Na (M + Na)^+^ 457.1351, found 457.1310. ^1^H NMR (500 MHz, CDCl_3_) δ 1.57 (d, *J* = 7.3 Hz, 4 H), 2.70 (s, 5 H), 4.35 (d, *J* = 5.6 Hz, 2 H), 4.77 (q, *J* = 7.3 Hz, 1 H), 5.23 (s, 2 H), 6.27 (br s, 1 H), 6.91 (d, *J* = 8.7 Hz, 3 H), 7.18 (d, *J* = 8.7 Hz, 3 H), 7.38–7.43 (m, 1 H), 7.54 (t, *J* = 7.6 Hz, 1 H), 7.69 (dd, *J* = 14.5, 7.8 Hz, 3 H); ^13^C NMR (126 MHz, CDCl_3_) δ 14.5, 28.3, 43.4, 49.8, 66.2, 66.2, 115.1, 124.4 (d, *J* = 273.8 Hz), 126.0 (q, *J* = 5.6 Hz), 127.3, 127.5, 127.8, 128.7, 129.2, 130.6, 132.3, 132.3, 135.6 (q, *J* = 1.6 Hz), 157.9, 168.5, 176.9. Anal. calcd for C_22_H_21_F_3_N_2_O_4_ (434.41): C: 60.83, H: 4.87, N: 6.45; Found C: 60.78, H: 4.92, N: 6.50.
2-(2,5-dioxopyrrolidin-1-yl)-*N*-(4-((3-(trifluoromethyl)benzyl)oxy)benzyl)propanamide (**13**) White solid. Yield: 83% (0.71 g); mp. 123.4–125.2 °C; TLC: R_f_ = 0.48 (S_2_); UPLC (purity 97.12%): *t*_R_ = 7.45 min, LC-MS (ESI): *m*/*z* calcd for C_22_H_21_F_3_N_2_O_4_ (M + H)^+^ 435.15, found 435.3 UPLC/HRMS (purity 97.01%): *t*_R_ = 6.97 min. HRMS (ESI-QTOF): *m*/*z* calcd for C_22_H_21_F_3_N_2_O_4_ (M + H)^+^ 435.1487, found 435.1508. ^1^H NMR (500 MHz, CDCl_3_) δ 1.59 (d, *J* = 7.3 Hz, 3 H), 2.73 (s, 4 H), 4.39 (d, *J* = 5.7 Hz, 2 H), 4.79 (q, *J* = 7.3 Hz, 1 H), 5.09 (s, 2 H), 6.15 (br s, 1 H), 6.93 (d, *J* = 8.7 Hz, 2 H), 7.21 (d, *J* = 8.7 Hz, 2 H), 7.45–7.52 (m, 1 H), 7.59 (dd, *J* = 12.4, 7.7 Hz, 2 H), 7.68 (s, 1 H); ^13^C NMR (126 MHz, CDCl_3_) δ 14.6, 28.3, 43.4, 49.9, 53.5, 69.3, 115.1, 124.5 (dd, *J* = 96.9, 3.9 Hz), 129.2, 129.3, 130.6, 130.7 (d, *J* = 1.3 Hz), 131.1 (d, *J* = 32.4 Hz), 138.0, 158.0, 168.4, 176.8. Anal. calcd for C_22_H_21_F_3_N_2_O_4_ (434.41): C: 60.83, H: 4.87, N: 6.45; Found C: 60.85, H: 4.79 N: 6.40.
2-(2,5-dioxopyrrolidin-1-yl)-*N*-(4-((4-(trifluoromethyl)benzyl)oxy)benzyl)propanamide (**14**) White solid. Yield: 77% (0.66 g); mp. 125.0–125.7 °C; TLC: R_f_ = 0.48 (S_2_); UPLC (purity > 99.99%): *t*_R_ = 7.43 min, LC-MS (ESI): *m*/*z* calcd for C_22_H_21_F_3_N_2_O_4_ (M + H)^+^ 435.15, found 453.3 UPLC/HRMS (purity > 99.99%): *t*_R_ = 7.10 min. HRMS (ESI-QTOF): *m*/*z* calcd for C_22_H_21_F_3_N_2_O_4_ (M + H)^+^ 435.1487, found 435.1508. ^1^H NMR (500 MHz, CDCl_3_) δ 1.57 (d, *J* = 7.3 Hz, 3 H), 2.71 (s, 4 H), 4.36 (d, *J* = 5.6 Hz, 2 H), 4.77 (q, *J* = 7.3 Hz, 1 H), 5.09 (s, 2 H), 6.24 (br s, 1 H), 6.90 (d, *J* = 8.7 Hz, 2 H), 7.19 (d, *J* = 8.7 Hz, 2 H), 7.52 (d, *J* = 8.2 Hz, 2 H), 7.62 (d, *J* = 8.2 Hz, 2 H); ^13^C NMR (126 MHz, CDCl_3_) δ 14.5, 28.3, 43.4, 49.8, 69.2, 115.1, 124.1 (d, *J* = 272.0 Hz), 125.6 (q, *J* = 3.8 Hz), 127.4, 129.3, 130.2 (d, *J* = 32.4 Hz), 130.6, 141.1 (d, *J* = 1.3 Hz), 157.9, 168.5, 176.9. Anal. calcd for C_22_H_21_F_3_N_2_O_4_ (434.41): C: 60.83, H: 4.87, N: 6.45; Found C: 60.77, H: 4.80 N: 6.51.
2-(2,5-dioxopyrrolidin-1-yl)-*N*-(4-((2-(trifluoromethoxy)benzyl)oxy)benzyl)propanamide (**15**) White solid. Yield: 87% (0.72 g); mp. 110.5–111.2 °C; TLC: R_f_ = 0.47 (S_2_); UPLC (purity > 99.99%): *t*_R_ = 7.51 min, LC-MS (ESI): *m*/*z* calcd for C_22_H_21_F_3_N_2_O_5_ (M + H)^+^ 451.14, found 451.2 UPLC/HRMS (purity > 99.99%): *t*_R_ = 7.11 min. HRMS (ESI-QTOF): *m*/*z* calcd for C_22_H_21_F_3_N_2_O_5_ (M + H)^+^ 451.1436, found 451.1451. ^1^H NMR (500 MHz, CDCl_3_) δ 1.57 (dd, *J* = 7.3, 1.1 Hz, 3 H), 2.71 (s, 4 H), 4.36 (d, *J* = 5.6 Hz, 2 H), 4.77 (q, *J* = 7.4 Hz, 1 H), 5.12 (s, 2 H), 6.23 (br s, 1 H), 6.89–6.96 (m, 2 H), 7.19 (d, *J* = 7.7 Hz, 2 H), 7.25–7.38 (m, 3 H), 7.57 (d, *J* = 7.6 Hz, 1 H); ^13^C NMR (126 MHz, CDCl_3_) δ 14.5, 28.3, 43.4, 49.8, 64.4, 115.1, 120.7 (q, *J* = 257.9 Hz), 120.7 (d, *J* = 1.3 Hz), 127.2, 129.2, 129.3, 129.4, 129.8, 130.5, 146.7 (q, *J* = 1.8 Hz), 158.0, 168.5, 176.9. Anal. calcd for C_22_H_21_F_3_N_2_O_5_ (450.41): C: 58.67, H: 4.70, N: 6.22; Found C: 58.71, H: 4.74 N: 6.18.
2-(2,5-dioxopyrrolidin-1-yl)-*N*-(4-((3-(trifluoromethoxy)benzyl)oxy)benzyl)propanamide (**16**) White solid. Yield: 78% (0.69 g); mp. 112.2–112.9 °C; TLC: R_f_ = 0.48 (S_2_); UPLC (purity 98.87%): *t*_R_ = 7.49 min, LC-MS (ESI): *m*/*z* calcd for C_22_H_21_F_3_N_2_O_5_ (M + H)^+^ 451.14, found 451.2 UPLC/HRMS (purity 98.91%): *t*_R_ = 7.21 min. HRMS (ESI-QTOF): *m*/*z* calcd for C_22_H_21_F_3_N_2_O_5_ (M + H)^+^ 451.1436, found 451.1451. ^1^H NMR (500 MHz, CDCl_3_) δ 1.60 (d, *J* = 7.3 Hz, 3 H), 2.74 (s, 4 H), 4.40 (d, *J* = 5.6 Hz, 2 H), 4.81 (q, *J* = 7.2 Hz, 1 H), 5.07 (s, 2 H), 6.20 (br s, 1 H), 6.94 (d, *J* = 8.4 Hz, 2 H), 7.18 (d, *J* = 8.0 Hz, 1 H), 7.22 (d, *J* = 8.3 Hz, 2 H), 7.30 (s, 1 H), 7.34–7.38 (m, 1 H), 7.39–7.44 (m, 1 H); ^13^C NMR (126 MHz, CDCl_3_) δ 14.6, 28.3, 43.4, 49.9, 53.5, 69.2, 115.1, 120.5 (d, *J* = 257.2 Hz), 119.8 (d, *J* = 0.9 Hz), 120.4 (d, *J* = 1.0 Hz), 125.5, 129.3, 130.1, 130.6, 139.4, 149.6 (d, *J* = 1.8 Hz), 158.0, 168.4, 176.8. Anal. calcd for C_22_H_21_F_3_N_2_O_5_ (450.41): C: 58.67, H: 4.70, N: 6.22; Found C: 58.64, H: 4.68 N: 6.24.
2-(2,5-dioxopyrrolidin-1-yl)-*N*-(4-((4-(trifluoromethoxy)benzyl)oxy)benzyl)propanamide (**17**) White solid. Yield: 84% (0.75 g); mp. 104.1–104.8 °C; TLC: R_f_ = 0.48 (S_2_); UPLC (purity > 99.99%): *t*_R_ = 7.52 min, LC-MS (ESI): *m*/*z* calcd for C_22_H_21_F_3_N_2_O_5_ (M + H)^+^ 451.14, found 451.2 UPLC/HRMS (purity > 99.99%): *t*_R_ = 7.25 min. HRMS (ESI-QTOF): *m*/*z* calcd for C_22_H_21_F_3_N_2_O_5_ (M + H)^+^ 451.1436, found 451.1451. ^1^H NMR (500 MHz, CDCl_3_) δ 1.57 (d, *J* = 7.3 Hz, 3 H), 2.71 (s, 4 H), 4.36 (d, *J* = 5.6 Hz, 2 H), 4.75–4.81 (m, 1 H), 5.02 (s, 2 H), 6.28 (br s, 1 H), 6.90 (d, *J* = 8.7 Hz, 2 H), 7.18 (d, *J* = 8.7 Hz, 2 H), 7.21 (d, *J* = 8.0 Hz, 2 H), 7.43 (d, *J* = 8.9 Hz, 2 H); ^13^C NMR (126 MHz, CDCl_3_) δ 14.6, 25.0, 25.7, 28.3, 34.0, 43.4, 49.8, 69.2, 115.1, 120.5 (q, *J* = 257.2 Hz), 121.2 (q, *J* = 1.0 Hz), 128.9, 129.2, 130.5, 135.7, 148.9, 148.9, 158.0, 168.5, 176.9. Anal. calcd for C_22_H_21_F_3_N_2_O_5_ (450.41): C: 58.67, H: 4.70, N: 6.22; Found C: 58.71, H: 4.73 N: 6.19.
2-(2,5-dioxopyrrolidin-1-yl)-*N*-(4-((2-methylbenzyl)oxy)benzyl)propanamide (**18**) White solid. Yield: 80% (0.60 g); mp. 126.1–126.8 °C; TLC: R_f_ = 0.43 (S_2_); UPLC (purity > 99.99%): *t*_R_ = 7.20 min, LC-MS (ESI): *m*/*z* calcd for C_22_H_24_N_2_O_4_ (M + H)^+^ 381.17, found 381.2 UPLC/HRMS (purity > 99.99%): *t*_R_ = 6.68 min. HRMS (ESI-QTOF): *m*/*z* calcd for C_22_H_24_N_2_O_4_Na (M + Na)^+^ 403.1634, found 403.1607. ^1^H NMR (500 MHz, CDCl_3_) δ 1.57 (d, *J* = 7.3 Hz, 3 H), 2.35 (s, 3 H), 2.70 (s, 4 H), 4.36 (d, *J* = 5.6 Hz, 2 H), 4.77 (q, *J* = 7.3 Hz, 1 H), 5.00 (s, 2 H), 6.26 (br s, 1 H), 6.94 (d, *J* = 8.6 Hz, 2 H), 7.17–7.22 (m, 5 H), 7.22–7.24 (m, 1 H), 7.38 (d, *J* = 7.4 Hz, 1 H); ^13^C NMR (126 MHz, CDCl_3_) δ 14.5, 19.0, 28.3, 43.4, 49.8, 68.7, 115.1, 115.1, 126.1, 128.4, 128.7, 129.2, 129.2, 130.2, 130.5, 134.7, 136.8, 136.8, 158.5, 158.5, 168.5, 177.0. Anal. calcd for C_22_H_24_N_2_O_4_ (380.44): C: 69.46, H: 6.36, N: 7.36; Found C: 69.50, H: 6.41 N: 7.38.
2-(2,5-dioxopyrrolidin-1-yl)-*N*-(4-((3-methylbenzyl)oxy)benzyl)propanamide (**19**) White solid. Yield: 82% (0.62 g); mp. 161.5–162.3 °C; TLC: R_f_ = 0.43 (S_2_); UPLC (purity 99.15%): *t*_R_ = 7.19 min, LC-MS (ESI): *m*/*z* calcd for C_22_H_24_N_2_O_4_ (M + H)^+^ 381.17, found 381.2 UPLC/HRMS (purity 99.13%): *t*_R_ = 6.63 min. HRMS (ESI-QTOF): *m*/*z* calcd for C_22_H_24_N_2_O_4_Na (M + Na)^+^ 403.1634, found 403.1607; ^1^H NMR (500 MHz, CDCl_3_) δ 1.57 (d, *J* = 7.3 Hz, 3 H), 2.35 (s, 3 H), 2.70 (s, 4 H), 4.36 (d, *J* = 5.6 Hz, 2 H), 4.77 (q, *J* = 7.3 Hz, 1 H), 5.00 (s, 2 H), 6.26 (br s, 1 H), 6.94 (d, *J* = 7.6 Hz, 2 H), 7.18 (s, 1 H), 7.20 (d, *J* = 5.6 Hz, 3 H), 7.23 (d, *J* = 1.6 Hz, 1 H), 7.24 (d, *J* = 3.7 Hz, 1 H), 7.38 (d, *J* = 7.4 Hz, 1 H); ^13^C NMR (126 MHz, CDCl_3_) δ 14.5, 14.6, 21.3, 21.3, 28.3, 43.5, 49.8, 49.8, 70.0, 115.1, 115.2, 115.2, 127.7, 129.1, 129.3, 129.3, 129.4, 129.5, 130.1, 133.9, 137.9, 158.4, 168.4, 176.9. Anal. calcd for C_22_H_24_N_2_O_4_ (380.44): C: 69.46, H: 6.36, N: 7.36; Found C: 69.43, H: 6.38 N: 7.41.
2-(2,5-dioxopyrrolidin-1-yl)-*N*-(4-((4-methylbenzyl)oxy)benzyl)propanamide (**20**) White solid. Yield: 79% (0.59 g); mp. 153.5–153.9 °C; TLC: R_f_ = 0.43 (S_2_); UPLC (purity 97.25%): *t*_R_ = 7.21 min, LC-MS (ESI): *m*/*z* calcd for C_22_H_24_N_2_O_4_ (M + H)^+^ 381.17, found 381.2 UPLC/HRMS (purity 97.51%): *t*_R_ = 6.76 min. HRMS (ESI-QTOF): *m*/*z* calcd for C_22_H_24_N_2_O_4_Na (M + Na)^+^ 403.1634, found 403.1607 ^1^H NMR (500 MHz, CDCl_3_) δ 1.56 (d, *J* = 7.3 Hz, 4 H), 2.34 (s, 3 H), 2.70 (s, 4 H), 4.35 (d, *J* = 5.6 Hz, 3 H), 4.76 (q, *J* = 7.3 Hz, 1 H), 4.99 (s, 2 H), 6.22 (br s, 1 H), 6.91 (d, *J* = 8.7 Hz, 3 H), 7.16 (d, *J* = 3.6 Hz, 3 H), 7.18 (d, *J* = 2.9 Hz, 3 H), 7.29 (d, *J* = 8.0 Hz, 3 H); ^13^C NMR (126 MHz, CDCl_3_) δ 14.5, 14.6, 21.3, 21.3, 28.3, 43.5, 49.8, 49.8, 70.0, 115.1, 115.2, 115.2, 127.7, 129.1, 129.3, 129.3, 129.4, 129.5, 130.1, 133.9, 137.9, 158.4, 168.4, 176.9. Anal. calcd for C_22_H_24_N_2_O_4_ (380.44): C: 69.46, H: 6.36, N: 7.36; Found C: 69.39, H: 6.41 N: 7.31.
*N*-(4-(benzyloxy)benzyl)-2-(2,5-dioxopyrrolidin-1-yl)-2-methylpropanamide (**21**) White solid. Yield: 83% (0.61 g); mp. 170.3–171.2 °C; TLC: R_f_ = 0.45 (S_2_); UPLC (purity > 99.99%): *t*_R_ = 6.93 min, LC-MS (ESI): *m*/*z* calcd for C_22_H_24_N_2_O_4_ (M + H)^+^ 381.17, found 381.2. UPLC/HRMS (purity > 99.99%): *t*_R_ = 6.39 min. HRMS (ESI-QTOF): *m*/*z* calcd for C_22_H_24_N_2_O_4_Na (M + Na)^+^ 403.1634, found 403.1613. ^1^H NMR (500 MHz, CDCl_3_) δ 1.71 (s, 6 H), 2.63 (s, 4 H), 4.37 (d, *J* = 5.4 Hz, 2 H), 5.04 (s, 2 H), 5.94 (br s, 1 H), 6.93 (d, *J* = 8.6 Hz, 2 H), 7.22 (d, *J* = 8.4 Hz, 2 H), 7.31 (s, 1 H), 7.35–7.39 (m, 2 H), 7.40 (s, 2 H); ^13^C NMR (126 MHz, CDCl_3_) δ 24.1, 28.5, 43.4, 61.7, 70.1, 70.1, 115.1, 127.5, 128.1, 128.7, 129.2, 129.3, 130.5, 137.0, 158.3, 172.6, 177.5. Anal. calcd for C_22_H_24_N_2_O_4_ (380.44): C: 69.46, H: 6.36, N: 7.36; Found C: 69.39, H: 6.30, N: 7.42
*N*-(3-(benzyloxy)benzyl)-2-(2,5-dioxopyrrolidin-1-yl)propanamide (**22**) White solid. Yield: 85% (0.62 g); mp. 105.3–106.1 °C; TLC: R_f_ = 0.40 (S_2_); UPLC (purity > 99.99%): *t*_R_ = 6.75 min, LC-MS (ESI): *m*/*z* calcd for C_21_H_22_N_2_O_4_ (M + H)^+^ 367.16, found 367.2 UPLC/HRMS (purity > 99.99%): *t*_R_ = 6.26 min. HRMS (ESI-QTOF): *m*/*z* calcd for C_21_H_22_N_2_O_4_Na (M + H)^+^ 367.1613, found 367.1671 ^1^H NMR (500 MHz, CDCl_3_) δ 1.56 (d, *J* = 7.3 Hz, 3 H), 2.66 (s, 4 H), 4.37 (d, *J* = 5.7 Hz, 2 H), 4.77 (q, *J* = 7.3 Hz, 1 H), 5.04 (s, 2 H), 6.41 (br s, 1 H), 6.82 (d, *J* = 7.4 Hz, 1 H), 6.84–6.88 (m, 2 H), 7.21 (t, *J* = 7.7 Hz, 1 H), 7.31 (d, *J* = 7.0 Hz, 1 H), 7.34–7.39 (m, 2 H), 7.39–7.44 (m, 2 H); ^13^C NMR (126 MHz, CDCl_3_) δ 14.5, 28.3, 43.8, 49.8, 70.0, 114.0, 114.2, 120.1, 127.6, 128.1, 128.7, 129.9, 137.0, 139.6, 159.2, 168.7, 176.9. Anal. calcd for C_21_H_22_N_2_O_4_ (366.42): C: 68.84, H: 6.05, N: 7.65; Found C: 68.70, H: 6.14, N: 7.69.
*N*-(2-(benzyloxy)benzyl)-2-(2,5-dioxopyrrolidin-1-yl)propanamide (**23**) White solid. Yield: 81% (0.58 g); mp. 113.4–114.2 °C; TLC: R_f_ = 0.42 (S_2_); UPLC (purity > 99.99%): *t*_R_ = 6.72 min, LC-MS (ESI): *m*/*z* calcd for C_21_H_22_N_2_O_4_ (M + H)^+^ 367.16, found 367.2. UPLC/HRMS (purity > 99.99%): *t*_R_ = 6.29 min. HRMS (ESI-QTOF): *m*/*z* calcd for C_21_H_22_N_2_O_4_Na (M + Na)^+^ 389.1477, found 389.1456. ^1^H NMR (500 MHz, CDCl_3_) δ 1.56 (d, *J* = 7.3 Hz, 3 H), 2.66 (s, 4 H), 4.37 (d, *J* = 5.7 Hz, 2 H), 4.77 (q, *J* = 7.3 Hz, 1 H), 5.04 (s, 2 H), 6.41 (br s, 1 H), 6.80–6.89 (m, 3 H), 7.21 (t, *J* = 7.7 Hz, 1 H), 7.28–7.33 (m, 1 H), 7.34–7.44 (m, 4 H); ^13^C NMR (126 MHz, CDCl_3_) δ 14.5, 28.3, 43.8, 49.8, 70.0, 114.0, 114.2, 120.1, 127.6, 128.1, 128.7, 129.9, 137.0, 139.6, 159.2, 168.7, 176.9. Anal. calcd for C_21_H_22_N_2_O_4_ (366.42): C: 68.84, H: 6.05, N: 7.65; Found C: 68.79, H: 6.01, N: 7.58.

#### 3.1.4. Synthetic Procedure for Boc-Protected Compounds **24**–**27**

To an anhydrous DCM (20 mL) solution of BOC-D,L-alanine (0.95 g, 5 mmol, 1 eq), we successively added DCC (1.55 g, 7.5 mmol, 1.5 eq) dissolved in 7 mL of DCM. After stirring (15 min), the appropriate amount of (4-(benzyloxy)phenyl)methanamine (5 mmol, 1.0 eq) dissolved in 5 mL of anhydrous DCM was added dropwise, and the reaction was stirred at room temperature for 2 h. The DCM was evaporated in vacuo, and the product was purified with column chromatography using a DCM:MeOH–9:0.5 (*v*/*v*) mixture as a solvent system.

*Tert*-butyl (1-((4-(benzyloxy)benzyl)amino)-1-oxopropan-2-yl)carbamate (**24**) Light oil. Yield: 96% (1.85 g); UPLC (purity 99.72%): *t*_R_ = 6.28 min. C_22_H_28_N_2_O_4_ (384.48). LC-MS (ESI): *m*/*z* calcd for C_22_H_28_N_2_O_4_ (M + H)^+^ 385.20, found 385.2.
*Tert*-butyl (1-oxo-1-((4-((2-(trifluoromethyl)benzyl)oxy)benzyl)amino)propan-2-yl)carbamate (**25**) Light oil. Yield: 94% (2.14 g); UPLC (purity 99.81%): *t*_R_ = 6.34 min. C_23_H_27_F_3_N_2_O_4_ (452.47). LC-MS (ESI): *m*/*z* calcd for C_23_H_27_F_3_N_2_O_4_ (M + H)^+^ 453.13, found 453.2
*Tert*-butyl (1-oxo-1-((3-((3-(trifluoromethyl)benzyl)oxy)benzyl)amino)propan-2-yl)carbamate (**26**) Light oil. Yield: 96% (2.18 g); UPLC (purity 99.64%): *t*_R_ = 6.35 min. C_23_H_27_F_3_N_2_O_4_ (452.47). LC-MS (ESI): *m*/*z* calcd for C_23_H_27_F_3_N_2_O_4_ (M + H)^+^ 453.13, found 453.1
*Tert*-butyl (1-oxo-1-((4-((4-(trifluoromethoxy)benzyl)oxy)benzyl)amino)propan-2-yl)carbamate (**27**) Light oil. Yield: 93% (2.19 g); UPLC (purity 99.58%): *t*_R_ = 6.42 min. C_23_H_27_F_3_N_2_O_5_ (468.47). LC-MS (ESI): *m*/*z* calcd for C_23_H_27_F_3_N_2_O_5_ (M + H)^+^ 469.19, found 469.2.

#### 3.1.5. Synthetic Procedure for Amines **28**–**31**

The DCM (5 mL) solution of one of the compounds **24**–**27** (4 mmol, 1 eq) was treated with TFA (1.37 g, 0.90 mL, 9 mmol, 3 eq) and stirred at room temperature for 3 h. Afterward, the organic solvents were evaporated in vacuo. The resulting oil residue was dissolved in water (20 mL), and then 25% ammonium hydroxide was carefully added to pH = 8. The aqueous layer was extracted with DCM (3 × 20 mL), dried over Na_2_SO_4_, and concentrated in vacuo to give compounds **28**–**31** as oils and were used to further reaction without purification.

2-amino-N-(4-(benzyloxy)benzyl)propanamide (**28**) Light oil. Yield: 97% (1.10 g); UPLC (purity 97.41%): *t*_R_ = 4.42 min. C_17_H_20_N_2_O_2_ (284.36). LC-MS (ESI): *m*/*z* calcd for C_17_H_20_N_2_O_2_ (M + H)^+^ 285.15, found 285.2.
2-amino-N-(4-((2-(trifluoromethyl)benzyl)oxy)benzyl)propanamide (**29**) Light oil. Yield: 95% (1.33 g); UPLC (purity 96.13%): *t*_R_ = 4.51 min. C_18_H_19_F_3_N_2_O_2_ (352.36). LC-MS (ESI): *m*/*z* calcd for C_18_H_19_F_3_N_2_O_2_ (M + H)^+^ 353.14, found 353.2.
2-amino-N-(4-((3-(trifluoromethyl)benzyl)oxy)benzyl)propanamide (**30**) Light oil. Yield: 94% (1.32 g); UPLC (purity 95.52%): *t*_R_ = 4.53 min. C_18_H_19_F_3_N_2_O_2_ (352.36). LC-MS (ESI): *m*/*z* calcd for C_18_H_19_F_3_N_2_O_2_ (M + H)^+^ 353.14, found 353.2.
2-amino-N-(4-((4-(trifluoromethoxy)benzyl)oxy)benzyl)propanamide (**31**) Light oil. Yield: 96% (1.41 g); UPLC (purity 96.82%): *t*_R_ = 4.64 min. C_18_H_19_F_3_N_2_O_3_ (368.36). LC-MS (ESI): *m*/*z* calcd for C_18_H_19_F_3_N_2_O_3_ (M + H)^+^ 369.19, found 369.2.

#### 3.1.6. Synthetic Procedure for Bromobutanamide **32**–**35**

To the DCM (50 mL) solution of 4-bromobutanoic acid (0.50 g, 3.0 mmol, 1.0 eq), we successively added DCC (0.93 g, 4.5 mmol, 1.5 eq) dissolved in 15 mL of DCM. After stirring (15 min), intermediate amine (3.0 mmol, 1 eq) was added, and the reaction was stirred at room temperature for 2 h. The DCM was evaporated in vacuo, and the product was purified with column chromatography using a DCM:MeOH–9:0.3 (*v*/*v*) mixture as a solvent system.

N-(1-((4-(benzyloxy)benzyl)amino)-1-oxopropan-2-yl)-4-bromobutanamide (**32**) Light oil. Yield: 89% (1.15 g); UPLC (purity 99.61%): *t*_R_ = 6.25 min. C_21_H_25_BrN_2_O_3_ (433.35). LC-MS (ESI): *m*/*z* calcd for C_21_H_25_BrN_2_O_3_ (M + H)^+^ 433.10, found 434.1
4-bromo-N-(1-oxo-1-((4-((2-(trifluoromethyl)benzyl)oxy)benzyl)amino)propan-2-yl)butanamide (**33**) Light oil. Yield: 92% (1.38 g); UPLC (purity 98.76%): *t*_R_ = 6.34 min. C_22_H_24_BrF_3_N_2_O_3_ (501.34). LC-MS (ESI): *m*/*z* calcd for C_21_H_25_BrN_2_O_3_ (M + H)^+^ 501.09, found 502.2
4-bromo-N-(1-oxo-1-((4-((3-(trifluoromethyl)benzyl)oxy)benzyl)amino)propan-2-yl)butanamide (**34**) Light oil. Yield: 94% (1.41 g); UPLC (purity 99.54%): *t*_R_ = 6.35 min. C_22_H_24_BrF_3_N_2_O_3_ (501.34). LC-MS (ESI): *m*/*z* calcd for C_21_H_25_BrN_2_O_3_ (M + H)^+^ 501.09, found 502.2
4-bromo-N-(1-oxo-1-((4-((4-(trifluoromethoxy)benzyl)oxy)benzyl)amino)propan-2-yl)butanamide (**35**) Light oil. Yield: 91% (1.40 g); UPLC (purity 99.78%): *t*_R_ = 6.41 min. C_22_H_24_BrF_3_N_2_O_4_ (517.34). LC-MS (ESI): *m*/*z* calcd for C_22_H_24_BrF_3_N_2_O_4_ (M + H)^+^ 517.09, found 518.2

#### 3.1.7. General Synthetic Procedure for Pyrrolidin-2-One Derivative **36**–**39**

NaH (0.10 g, 4 mmol, 2 eq) was added to a solution of the appropriate amount of bromobutanamide **32**–**35** (2 mmol, 1 eq) in anhydrous THF. The reaction mixture was stirred for 3 h and next concentrated under reduced pressure. The oily residue was dissolved in 0.1 M HCl (50 mL) and extracted with DCM (3 × 50 mL). The organic layer was dried over anhydrous Na_2_SO_4_ and evaporated to dryness. The crude product was purified with column chromatography using a DCM:MeOH (9:0.5; *v*/*v*) solvent system. After washing with Et_2_O, the compound was obtained as a white solid.

N-(4-(benzyloxy)benzyl)-2-(2-oxopyrrolidin-1-yl)propanamide (**36**) White solid. Yield: 86% (0.61 g); mp. 110.5–110.9 °C; TLC: R_f_ = 0.44 (S_2_); UPLC (purity > 99.99%): *t*_R_ = 6.70 min, LC-MS (ESI): *m*/*z* calcd for C_21_H_24_N_2_O_3_ (M + H)^+^ 353.18, found 353.3 UPLC/HRMS (purity > 99.99%): *t*_R_ = 6.17 min. HRMS (ESI-QTOF): *m*/*z* calcd for C_21_H_24_N_2_O_3_ (M + H)^+^ 353.1820, found 353.1862. ^1^H NMR (500 MHz, CDCl_3_) δ 1.4 (d, *J* = 7.2 Hz, 3 H), 1.9–2.0 (m, 2 H), 2.3–2.4 (m, 2 H), 3.3–3.5 (m, 2 H), 4.3 (d, *J* = 5.9 Hz, 2 H), 4.7 (q, *J* = 7.2 Hz, 1 H), 5.0 (s, 2 H), 6.6 (br s, 1 H), 6.9 (d, *J* = 8.7 Hz, 2 H), 7.1 (d, *J* = 8.7 Hz, 2 H), 7.3 (d, *J* = 7.2 Hz, 1 H), 7.3–7.4 (m, 2 H), 7.4 (s, 2 H); ^13^C NMR (126 MHz, CDCl_3_) δ 13.8, 18.1, 31.2, 43.0, 43.8, 50.3, 70.1, 115.1, 127.5, 128.1, 128.7, 129.0, 130.7, 137.0, 158.2, 170.5, 175.8. Anal. calcd for C_21_H_24_N_2_O_3_ (352.18): C: 71.57, H: 6.86, N: 7.95; Found C: 71.49, H: 6.80, N: 7.99.
2-(2-oxopyrrolidin-1-yl)-N-(4-((2-(trifluoromethyl)benzyl)oxy)benzyl)propanamide (**37**) White solid. Yield: 84% (0.70 g); mp. 121.8–122.5 °C; TLC: R_f_ = 0.45 (S_2_); UPLC (purity > 99.99%): *t*_R_ = 7.45 min, LC-MS (ESI): *m*/*z* calcd for C_22_H_23_F_3_N_2_O_3_ (M + H)^+^ 421.17, found 421.3 UPLC/HRMS (purity > 99.99%): *t*_R_ = 6.88 min. HRMS (ESI-QTOF): *m*/*z* calcd for C_22_H_23_F_3_N_2_O_3_ (M + H)^+^ 421.1694, found 421.1716 ^1^H NMR (500 MHz, CDCl_3_) δ 1.35 (d, *J* = 7.2 Hz, 3 H), 1.79 (s, 1 H), 1.95–1.99 (m, 1 H), 2.27–2.43 (m, 2 H), 3.33–3.46 (m, 2 H), 4.31 (d, *J* = 5.9 Hz, 2 H), 4.66 (q, *J* = 7.2 Hz, 1 H), 5.24 (s, 2 H), 6.58–6.64 (m, 1 H), 6.88 (d, *J* = 8.6 Hz, 2 H), 7.14 (d, *J* = 8.6 Hz, 2 H), 7.37–7.43 (m, 1 H), 7.54 (t, *J* = 7.7 Hz, 1 H), 7.69 (dd, *J* = 14.3, 7.9 Hz, 2 H); ^13^C NMR (126 MHz, CDCl_3_) δ 13.8, 18.1, 31.2, 42.9, 43.8, 50.4, 66.2, 66.2, 115.1, 124.4 (d, *J* = 273.8 Hz), 126.0 (q, *J* = 5.6 Hz), 127.4 (d, *J* = 30.9 Hz), 127.8, 128.6, 129.1, 131.0, 132.2 (d, *J* = 1.0 Hz), 135.7, 157.7, 170.5, 175.8. Anal. calcd for C_22_H_23_F_3_N_2_O_3_ (420.43): C: 62.85, H: 5.51, N: 6.66; Found C: 62.79, H: 5.48, N: 6.59.
2-(2-oxopyrrolidin-1-yl)-N-(4-((3-(trifluoromethyl)benzyl)oxy)benzyl)propanamide (**38**) White solid. Yield: 80% (0.67 g); mp. 123.5–123.8 °C; TLC: R_f_ = 0.47 (S_2_); UPLC (purity > 99.99%): *t*_R_ = 7.48 min, LC-MS (ESI): *m*/*z* calcd for C_22_H_23_F_3_N_2_O_3_ (M + H)^+^ 421.17, found 421.3 UPLC/HRMS (purity > 99.99%): *t*_R_ = 6.88 min. HRMS (ESI-QTOF): *m*/*z* calcd for C_22_H_23_F_3_N_2_O_3_ (M + H)^+^ 421.1694, found 421.1716. ^1^H NMR (500 MHz, CDCl_3_) δ 1.35 (d, *J* = 7.2 Hz, 3 H), 1.79 (s, 1 H), 1.93–2.02 (m, 1 H), 2.26–2.43 (m, 2 H), 3.34–3.45 (m, 2 H), 4.31 (d, *J* = 5.9 Hz, 2 H), 4.66 (q, *J* = 7.2 Hz, 1 H), 5.24 (s, 2 H), 6.58–6.64 (m, 1 H), 6.88 (d, *J* = 8.6 Hz, 2 H), 7.14 (d, *J* = 8.6 Hz, 2 H), 7.37–7.43 (m, 1 H), 7.54 (t, *J* = 7.7 Hz, 1 H), 7.65–7.73 (m, 2 H); ^13^C NMR (126 MHz, CDCl_3_) δ 13.8, 18.1, 31.2, 42.9, 43.8, 50.4, 66.2, 66.2, 115.1, 124.4 (d, *J* = 273.8 Hz), 126.0 (q, *J* = 5.6 Hz), 127.4 (d, *J* = 30.9 Hz), 127.8, 128.6, 129.1, 131.0, 132.2 (d, *J* = 1.0 Hz), 135.7, 157.7, 170.5, 175.8. Anal. calcd for C_22_H_23_F_3_N_2_O_3_ (420.43): C: 62.85, H: 5.51, N: 6.66; Found C: 62.798, H: 5.46, N: 6.69.
2-(2-oxopyrrolidin-1-yl)-N-(4-((4-(trifluoromethoxy)benzyl)oxy)benzyl)propanamide (**39**) White solid. Yield: 87% (0.76 g); mp. 103.4–103.9 °C; TLC: R_f_ = 0.48 (S_2_); UPLC (purity 99.75%): *t*_R_ = 7.75 min, LC-MS (ESI): *m*/*z* calcd for C_22_H_23_F_3_N_2_O_4_ (M + H)^+^ 437.16, found 437.3 UPLC/HRMS (purity 99.82%): *t*_R_ = 7.20 min. HRMS (ESI-QTOF): *m*/*z* calcd for C_22_H_23_F_3_N_2_O_4_Na (M + Na)^+^ 459.1508, found 459.1549 ^1^H NMR (500 MHz, CDCl_3_) δ 1.35 (d, *J* = 7.2 Hz, 3 H), 1.87 (br s, 1 H), 1.95–2.00 (m, 1 H), 2.27–2.40 (m, 2 H), 3.34–3.45 (m, 2 H), 4.31 (d, *J* = 5.9 Hz, 2 H), 4.66 (q, *J* = 7.2 Hz, 1 H), 5.02 (s, 2 H), 6.66 (br s, 1 H), 6.88 (d, *J* = 8.7 Hz, 2 H), 7.14 (d, *J* = 8.6 Hz, 2 H), 7.21 (d, *J* = 8.0 Hz, 2 H), 7.43 (d, *J* = 8.7 Hz, 2 H); ^13^C NMR (126 MHz, CDCl_3_) δ 13.8, 18.1, 31.2, 42.9, 43.8, 50.4, 69.2, 115.0, 119.5, 121.2 (q, *J* = 0.9 Hz), 121.5, 128.9, 129.1, 131.0, 135.7, 148.9 (q, *J* = 1.8 Hz), 157.9, 170.5, 175.8. Anal. calcd for C_22_H_23_F_3_N_2_O_4_ (436.43): C: 60.55, H: 5.31, N: 6.42; Found C: 60.47, H: 5.24, N: 6.35.

### 3.2. In Vivo Studies, Anticonvulsant Activity, and Neurotoxicity Studies

In this study, we utilized adult male albino Swiss mice (CD-1) weighing between 22 and 26 g. The mice were maintained under standardized conditions in colony cages with unrestricted access to food and tap water. Each experimental group comprised four mice that were randomly assigned, with each mouse being used only once for the initial anticonvulsant screening. All animal procedures and care adhered to current European Community and Polish legislation on animal experimentation. The research protocols were approved by the Local Ethical Committee in Cracow, Poland (Nos. 276/2019, and 654/2022), and experimental procedures complied with the European Union Directive of 22 September 2010 (2010/63/EU) and relevant Polish regulations.

All test substances were suspended in a 1% aqueous solution of Tween 80 and administered intraperitoneally (i.p.) at a dose of 10 mL/kg body weight. Fresh solutions were prepared daily for each experimentation session. Detailed in vivo procedures, including the maximal electroshock seizure test (MES) [23], the 6 Hz (32 and 44 mA) psychomotor seizure model [42], and the rotarod test for acute neurological toxicity, have been described elsewhere [43].

#### Data Analysis

To evaluate the ED_50_ or TD_50_, 3–4 groups of six mice were injected with various doses of tested compounds. Each group consisted of six animals. Both ED_50_ and TD_50_ values with 95% confidence limits were calculated by probit analysis [29]. Protective indexes for the compounds under investigation and reference antiseizure drugs (ASMs) were determined by dividing the TD_50_ value, obtained from the rotarod test, by the corresponding ED_50_ value from the MES or 6 Hz (32 mA or 44 mA) tests. The protective index serves as an indicator of the safety margin and tolerability, reflecting the difference between anticonvulsant doses and doses causing acute adverse effects, such as sedation, impaired motor coordination, ataxia, or other neurotoxic symptoms. 

### 3.3. In Vitro Pharmacology and Tox Studies

#### 3.3.1. Radioligand Binding/Functional Assays

Binding/functional studies were performed commercially in Cerep Laboratories (Poitiers, France) using testing procedures described elsewhere. The general information is listed in Table S3 in the Supporting Information.

#### 3.3.2. Hepatocytotoxicity and Neurocytotoxicity Assessment

To evaluate selected compounds’ hepatocytotoxicity and neurocytotoxicity, a human hepatocellular carcinoma cell line, HepG2 (ATCC^®^ HB-8065™), and a human neuroblastoma cell line, SH-SY5Y (ATCC^®^, CRL-2266™), were used. Both cell lines were cultured in standard culture conditions (5% CO_2_, 37 °C, 95% humidity) using Dulbecco′s Modified Eagle′s—Medium (DMEM; Gibco, Thermo Scientific, Waltham, MA, USA) supplemented with 10% (vol/vol) fetal bovine serum (FBS; Gibco, Thermo Scientific, Waltham, MA, USA) and an antibiotic mixture (Penicillin, Streptomycin, Amphotericin B; Gibco, Thermo Scientific, Waltham, MA, USA). Cells were cultured with compounds **5**, **7**, or **39** (1–50 µM) for 24 h and then an MTT (3-(4,5-dimethylthiazol-2-yl)-2,5diphenyltetrazolium bromide, Sigma-Aldrich, St. Louis, MO, USA) viability assay was performed. The formazan crystals were dissolved in DMSO (Sigma-Aldrich, St. Louis, MO, USA), and the absorbance at 570 nm was measured using a microplate reader (SpectraMax^®^ iD3, Molecular Devices, San Jose, CA, USA). The experiment was performed three times in duplicate. Each bar represents the mean (±SEM) percentage of viable cells in comparison to the control (defined as 100% and represented by untreated cells). 

#### 3.3.3. Monoamine Oxidase Assays

The inhibition activity of evaluated compounds was measured using human recombinant MAO-B (Sigma Aldrich) in the fluorometric method for detecting monoamine oxidase activity. The assay was carried out in a 96-well plate. A total of 2 μL of the appropriate concentration of tested compounds in DMSO were added to wells that contained 98 μL of enzyme dilution (0.53 U/mL) in phosphate buffer (50 mM, pH 7.4). After 30 min of preincubation at room temperature, 50 μL of the solution of 800 μM10-acetyl-3,7 dihydroxyphenoxazine (Cayman Chemical Company, Ann Arbor, MI, USA, 10010469) and 4 U/mL of horse radish peroxidase (HRP, Sigma Aldrich, St. Louis, MO, USA, P6782) were added, and an enzymatic reaction was started with the addition of 50 μL of 800 μM p-tyramine (Alfa Aesar, Haverhill, MA, USA, A12220) solution. The signal was measured after 1 h (excitation at 570 nm and emission at 585 nm) using an EnSpire^®^ multimode plate reader (PerkinElmer, Inc., Waltham, MA, USA). Rasagiline (1 μM) was tested as a reference compound for MAO-B [44]. 

## 4. Conclusions

The present chemical and pharmacological studies led to the identification of new alaninamide derivatives with broad-spectrum anticonvulsant activity. Compound **5** emerged as a lead molecule, demonstrating robust protection in several acute seizure models in mice (i.p.), namely the MES, 6 Hz (32 mA), and 6 Hz (44 mA). Importantly, compound **5** showed a low propensity for inducing motor impairment in the rotarod test, suggesting a favorable therapeutic window. In vitro toxicity assays further supported its safety profile, making compound **5** a promising candidate for further preclinical development.

Preliminary studies have been conducted to evaluate the mechanism of action of compound **5**. The results of the in vitro binding and functional assays demonstrate that compound **5**, at a concentration of 100 µM, exhibits significant inhibition of the Cav_1_._2_ (L-type) calcium channel, with an inhibition rate exceeding 50%. This suggests that the antagonism of the Cav_1_._2_ (L-type) calcium channels may play a role in the anticonvulsant properties of this compound. Nevertheless, more detailed insight into the pharmacodynamics of lead compound **5** is necessary, especially in electrophysiology and glutamate uptake studies. Furthermore, future research should also assess the compound’s activity in pain models to explore its potential as an antinociceptive agent. Finally, compounds reported in the current studies are racemates; therefore, further development will include asymmetric synthesis and the comprehensive pharmacological characterization of respective enantiomers.

## Figures and Tables

**Table 1:** Drug-like parameters estimated according to Lipinski’s and Veber’s rules, as well as CNS MPO scores.

Cmpd	R	Lipinski Rules				Violations of Rules	Veber		CNS MPO ^e^
		MW ≤500	Log P ≤5	NHD ^a^ ≤5	NHA ^b^ ≤10		NBR ^c^ ≤10	PSA ^d^ ≤140 Å^2^	
**5**	H	366.41	2.20	1	4	0	8	75.71	5.34
**6**	2-F	384.40	2.60	1	5	0	8	75.71	5.29
**7**	3-F	384.40	2.56	1	5	0	8	75.71	5.31
**8**	4-F	384.40	2.57	1	5	0	8	75.71	5.30
**9**	2-Cl	400.86	2.77	1	4	0	8	75.71	5.17
**10**	3-Cl	400.86	2.79	1	4	0	8	75.71	5.16
**11**	4-Cl	400.86	2.80	1	4	0	8	75.71	5.15
**12**	2-CF_3_	434.41	3.26	1	7	0	9	75.71	4.87
**13**	3-CF_3_	434.41	3.29	1	7	0	9	75.71	4.85
**14**	4-CF_3_	434.41	3.30	1	7	0	9	75.71	4.84
**15**	2-OCF_3_	450.41	3.07	1	8	0	10	84.94	4.57
**16**	3-OCF_3_	450.41	3.14	1	8	0	10	84.94	4.53
**17**	4-OCF_3_	450.41	3.10	1	8	0	10	84.94	4.55
**18**	2-CH_3_	380.44	2.61	1	4	0	10	75.71	5.24
**19**	3-CH_3_	380.44	2.60	1	4	0	10	75.71	5.25
**20**	4-CH_3_	380.44	2.61	1	4	0	10	75.71	5.24
**21**	H	380.44	2.49	1	4	0	8	75.71	5.31
**22**	H	366.41	2.29	1	4	0	8	75.71	5.33
**23**	H	366.41	2.27	1	4	0	8	75.71	5.34
**36**	H	352.43	2.69	1	3	0	8	58.64	5.64
**37**	2-CF_3_	420.42	3.70	1	6	0	9	58.64	4.69
**38**	3-CF_3_	420.42	3.72	1	6	0	9	58.64	4.67
**39**	4-OCF_3_	436.42	3.56	1	7	0	10	67.87	4.57

^a^ NHD—number of hydrogen bond donors, ^b^ NHA—number of hydrogen bond acceptors, ^c^ NBR—number of rotatable bonds, ^d^ TPSA—total polar surface area, ^e^ CNS MPO—central nervous system multiparameter optimization scores were calculated using the Instant JChem 21.4.0 software (ChemAxon, Budapest, Hungary).

**Table 2:** The quantitative pharmacological parameters ED_50_, TD_50_, and PIs in mice i.p.

Cmpd	TPE (h) ^a^	ED_50_ MES (mg/kg) ^b^	ED_50_ 6 Hz (32 mA) (mg/kg) ^c^	TD_50_ (mg/kg) ^d^	PI (TD_50_/ED_50_) ^e^
**5**	0.5	**48.00** (41.91–54.96)	**45.19** (37.31–54.73)	>300	>6.3 (MES) >6.6 (6 Hz)
**7**	0.5	78.30 (65.82–93.13)	71.73 (66.6–77.23)	>300	>3.8 (MES) >4.2 (6 Hz)
**9**	0.5	48.00 (41.9–54.96)	57.69 (33.98–97.94)	>300	>6.3 (MES) >5.2 (6 Hz)
**10**	0.5	50.87 (45.29–57.13)	59.31 (45.54–73.08)	>300	>5.9 (MES) >5.0 (6 Hz)
**12**	0.5	**46.19** (40.44–52.76)	**52.66** (37.70–73.56)	>300	>6.5 (MES) >5.7 (6 Hz)
**13**	0.5	69.72 (64.72–75.12)	67.93 (48.77–94.61)	>300	>4.3 (MES) >4.4 (6 Hz)
**16**	0.5	**40.55** (33.55–49.02)	**62.95** (45.71–86.69)	>300	>7.4 (MES) >4.8 (6 Hz)
**17**	0.5	**21.03** (10.8–40.85)	**77.00** (58.06–102.12)	>300	>14.3 (MES) >3.9 (6 Hz)
**18**	0.5	89.20 (69.25–114.90)	57.69 (33.98–97.94)	>300	>3.4 (MES) >5.2 (6 Hz)
**38**	0.5	54.83 (43.54–69.05)	65.28 (37.25–114.41)	>300	>5.5 (MES) >4.6 (6 Hz)
**39**	0.5	**36.20** (32.34–40.53)	**37.27** (23.00–60.41)	<300	<8.3 (MES) <8.0 (6 Hz)
**(*R*)-AS-1** *	0.5	66.3 (53.6–82.0)	15.6 (9.1–26.9)	>500	>7.5 (MES) >32.0 (6 Hz)
**LCS ^f^**	0.5	9.2 (8.5–10.0)	5.3 (3.5–7.8)	46.2 (44.5–48.0)	5.0 (MES) 8.8 (6 Hz)
**LEV ^f^**	1.0	>500	15.7 (10.4–23.7)	>500	>31.8 (6 Hz)
**VPA ^f^**	0.5	252.7 (220.1–290.2)	130.6 (117.6–145.2)	430.7 (407.9–454.9)	1.7 (MES) 3.3 (6 Hz)

The data for the most potent compounds have been bolded for better visualization. Values in parentheses are 95% confidence intervals determined using probit analysis [29]. ^a^ Time to peak effect. ^b^ ED_50_ (MES-maximal electroshock seizure test). ^c^ ED_50_ (6 Hz seizure test, 32 mA). ^d^ TD_50_ (NT-acute neurological deficit determined in the rotarod test). ^e^ Protective index (TD_50_/ED_50_). ^f^ Reference ASMs: lacosamide (LCS), levetiracetam (LEV), and valproic acid (VPA) tested in the same conditions [19]. TPEs for model ASMs taken from own experiments or literature [30]. * Data for (***R***)-**AS-1** have been published previously; see compound (***R***)-**7** in ref. [11].

**Table 3:** The quantitative data in the 6 Hz (44 mA) test in mice i.p.

Cmpd	TPE (h) ^a^	ED_50_ 6 Hz (44 mA) (mg/kg) ^b^	TD_50_ (mg/kg) ^c^	PI (TD_50_/ED_50_) ^d^
**5**	0.5	201.3 (152.4–265.8)	>300	>1.5
**(*R*)-AS-1** *	0.5	41.6 (32.8–52.7)	>500	>12.0
**LCS ^e^**	0.5	6.9 (5.4–8.6)	46.2 (44.5–48.0)	6.7
**LEV ^e^**	1.0	>500	>500	n.c.
**VPA ^e^**	0.5	183.1 (143.5–233.7)	430.7 (407.9–454.9)	2.3

Values in parentheses are 95% confidence intervals determined using probit analysis [29]. ^a^ Time to peak effect. ^b^ ED_50_ (6 Hz-psychomotor seizure test, 44 mA). ^c^ TD_50_ (NT-acute neurological toxicity determined in the rotarod test). ^d^ Protective index (TD_50_/ED_50_). ^e^ Data from ref. [32]. * Data for (***R***)**-AS-1** have been published previously; see compound (***R***)-**7** in ref. [11].

**Table 4:** In vitro binding and functional assays for compound **5**.

Binding Studies	Source	% Inhibition of Control Specific Binding (Concentration [µM]) ^a^
Na^+^ channel (site 2)	Rat cerebral cortex	38.2 (100)
Cav_2_._2_ N-type (antagonist radioligand)	Rat cerebral cortex	1.1 (100)
Cav_1_._2_ (L-type) (dihydropyridine site, antagonist radioligand)	Rat cerebral cortex	**82.3** (100)
Cav_1_._2_ (L-type) (diltiazem site, antagonist radioligand)	Rat cerebral cortex	**68.5** (100)
Cav_1_._2_ (L-type) (verapamil site, antagonist radioligand)	Rat cerebral cortex	**57.1** (100)
GABA transporter (antagonist radioligand)	Rat cerebral cortex	0.3 (100)
GlyT1 (antagonist radioligand)	Rat cerebral cortex	23.0 (100)
Potassium channel (hERG)	Human recombinant HEK-293 cell	4.5 (100)
**Functional Studies**	**Source**	**% Inhibition of Control Agonist Response (Concentration [µM]) ^a^**
Cav_1_._2_ (*h*) calcium ion channel cell-based antagonist calcium flux assay	Human recombinant HEK-293 cell	**85.0** (100)

^a^ Results showing activity higher than 50% are considered to represent significant effects of the test compounds have been bolded for better visualization; results showing an inhibition between 25% and 50% are indicative of weak effect; results showing an inhibition lower than 25% are not considered significant and mostly attributable to variability of the signal around the control level. Binding studies were performed commercially in Eurofins Laboratories (Poitiers, France).

## Data Availability

Data is contained within the article and Supplementary Material.

## Supplementary Materials

The following are available online at https://www.mdpi.com/article/10.3390/ijms25189861/s1. References [45,46,47,48,49,50,51,52,53,54] are cited in the supplementary materials.

## Abbreviations

ASMs, antiseizure medications; CDI, carbonyldiimidazole; CNS, central nervous system; DCM, dichloromethane; DCC, *N*,*N*′-dicykloheksylokarbodiimid; DDIs, drug-drug interactions; DRE, drug-resistant epilepsy; GABA, gamma-aminobutyric acid; 6 Hz, six-Hertz seizure test; LCS, Lacosamide; LEV, Levetiracetam; MAO-B, monoamine oxidase B; MeCN, acetonitrile; MES, maximal electroshock seizure test; MeOH, methanol; PI, protective index (TD_50_/ED_50_); SAF, safinamide; *sc*PTZ, subcutaneous pentylenetetrazole seizure test; TFA, trifluoroacetic acid; TP, time of peak effect; VPA, valproic acid.
