# Supplementary material for: Development of Novel Alaninamide Derivatives with Anticonvulsant Activity and Favorable Safety Profiles in Animal Models
Source: Int J Mol Sci. 2024 Sep 12;25(18):9861. doi: 10.3390/ijms25189861 (PMC11432405; doi:10.3390/ijms25189861)

Supplementary data for

Development of Novel Alaninamide Derivatives with Anticonvulsant Activity and Favorable Safety Profiles in Animal Models

Michał Abram [1,*], Marcin Jakubiec [1], Paulina Koczurkiewicz-Adamczyk [2], Agata Doroz-Płonka [3], Anna Rapacz [4,*] and Krzysztof Kamiński [1]

[1] Department of Medicinal Chemistry, Faculty of Pharmacy, Jagiellonian University Medical College, Medyczna 9, 30-688 Kraków, Poland; marcin.jakubiec@uj.edu.pl (M.J.); k.kaminski@uj.edu.pl (K.K.)
[2] Department of Pharmaceutical Biochemistry, Faculty of Pharmacy, Jagiellonian University Medical College, Medyczna 9, 30-688 Kraków, Poland; paulina.koczurkiewicz@uj.edu.pl
[3] Department of Technology and Biotechnology of Drugs, Faculty of Pharmacy, Jagiellonian University Medical College, Medyczna 9, 30-688 Kraków, Poland; a.doroz-plonka@uj.edu.pl
[4] Department of Pharmacodynamics, Faculty of Pharmacy, Jagiellonian University Medical College, Medyczna 9, 30-688 Kraków, Poland
* Correspondence: michal.abram@uj.edu.pl (M.A.); a.rapacz@uj.edu.pl (A.R.); Tel.: +48-12-620-54-59 (M.A.); Fax: +48 12 620 54 58 (M.A.)

Table of contents

Table S1. Anticonvulsant activity screening data in the MES and 6 Hz (32 mA) seizure models in mice *i.p.* (dose of 100 mg/kg) pretreatment time–0.5 h. 2

General synthetic procedure for (benzyloxy)benzonitrile A1–A18 3

General synthetic procedure for (benzyloxy)phenyl)methanamine A19–A36 6

Table S2. Inhibitory effects of obtained compounds on MAO-B. 9

Table S3. The general information of radioligand binding/functional assays. 10

References 11

UPLC/HRMS traces for target final compounds 12

^{1}H NMR, ^{13}C NMR spectra for all final compounds 35

Table S1. Anticonvulsant activity screening data in the MES and 6 Hz (32 mA) seizure models in mice *i.p.* (dose of 100 mg/kg) pretreatment time–0.5 h.

Compd	R	R[1]	MES test [a]	6 Hz (32 mA) test [a]
**Pyrrolidine-2,5-dione derivatives**				
**5**	H	H	**4/4**	**2/4**
**6**	2-F	H	**4/4**	**4/4**
**7**	3-F	H	1/4	**4/4**
**8**	4-F	H	**2/4**	**2/4**
**9**	2-Cl	H	**4/4**	**3/4**
**10**	3-Cl	H	**4/4**	**3/4**
**11**	4-Cl	H	**0/4**	**1/4**
**12**	2-CF_3	H	**4/4**	**3/4**
**13**	3-CF_3	H	**4/4**	**4/4**
**14**	4-CF_3	H	**2/4**	**2/4**
**15**	2-OCF_3	H	**2/4**	**1/4**
**16**	3-OCF_3	H	**4/4**	**2/4**
**17**	4-OCF_3	H	**4/4**	**3/4**
**18**	2-CH_3	H	**3/4**	**3/4**
**19**	3-CH_3	H	1/4	1/4
**20**	4-CH_3	H	0/4	0/4
**21**	H	CH_3	1/4	0/4
**22**	H	H	1/4	0/4
**23**	H	H	1/4	0/4
**Pyrrolidin-2-one derivatives**				
**36**	H	H	**4/4**	1/4
**37**	2-CF_3	H	**4/4**	**2/4**
**38**	3-CF_3	H	**4/4**	**3/4**
**39**	4-OCF_3	H	**4/4**	**4/4**

Ratios where at least 50% of animals were protected have been highlighted in bold for easier data interpretation.

[a] Data indicate: number of mice protected / number of mice tested. The animals were examined at one pretreatment time–0.5 h. MES – the maximal electroshock seizure test. 6 Hz (32 mA) seizure test,.

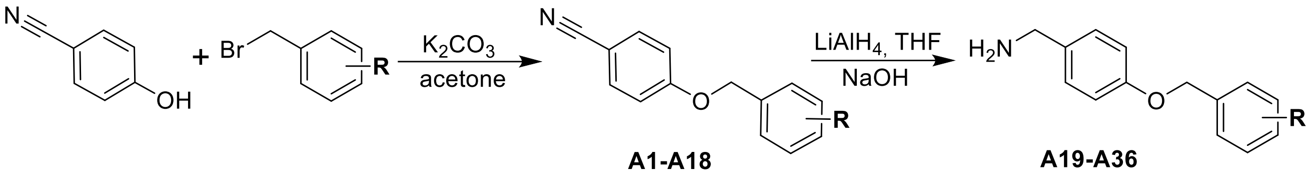

A1-A18

A1: **R** = H, **A10:** **R** = 4-CF_3,
A2: **R** = 2-F, **A11:** **R** = 2-OCF_3,
A3: **R** = 3-F, **A12:** **R** = 3-OCF_3,
A4: **R** = 4-F, **A13:** **R** = 4-OCF_3,
A5: **R** = 2-Cl, **A14:** **R** = 2-CH_3,
A6: **R** = 3-Cl, **A15:** **R** = 3-CH_3,
A7: **R** = 4-Cl, **A16:** **R** = 4-CH_3
A8: **R** = 2-CF_3, **A17:** ***ortho*** **R** = H,
A9: **R** = 3-CF_3, **A18:** ***meta*** **R** = H,

A19-A36

A19: **R** = H, **A28:** **R** = 4-CF_3
A20: **R** = 2-F, **A29:** **R** = 2-OCF_3,
A21: **R** = 3-F, **A30:** **R** = 3-OCF_3,
A22: **R** = 4-F, **A31:** **R** = 4-OCF_3,
A23: **R** = 2-Cl, **A32:** **R** = 2-CH_3,
A24: **R** = 3-Cl, **A33:** **R** = 3-CH_3,
A25: **R** = 4-Cl, **A34:** **R** = 4-CH_3,
A26: **R** = 2-CF_3, **A35:** ***ortho*** **R** = H,
A27: **R** = 3-CF_3, **A36:** ***meta*** **R** = H,

Scheme S1. Synthesis of intermediates benzonitrile **A1–A18** and amines **A19–A36**.

General synthetic procedure for (benzyloxy)benzonitrile A1–A18

The appropriate cyanophenol (3.57 g, 30 mmol, 1 eq) was dissolved in acetone (100 ml). Anhydrous potassium carbonate (16.58 g, 120 mmol, 4 eq) was added, followed by the dropwise addition of the appropriate benzyl bromide (30 mmol, 1 eq). The reaction mixture was heated to 60°C and stirred for 12 hours. After the reaction was complete, the solvent was evaporated, and the resulting derivative was crystallized from anhydrous ethanol.

4-(benzyloxy)benzonitrile (A1)

White solid. Yield: 83% (5.2 g); UPLC (purity: 98.2%): t_R = 3.89 min, LC-MS (ESI): *m/z* calcd for $C_{14}H_{11}NO$ $(M+H)^+$ 210.08, found 210.10. ^1H NMR (300 MHz, $CDCl_3$) δ 5.12 (s, 2 H), 6.95–7.08 (m, 2 H), 7.21–7.46 (m, 5 H), 7.52–7.65 (m, 2 H).

4-((2-fluorobenzyl)oxy)benzonitrile (A2)

White solid. Yield: 78% (5.31 g); UPLC (purity: 97.5%): t_R = 3.93 min, LC-MS (ESI): *m/z* calcd for $C_{14}H_{10}FNO$ $(M+H)^+$ 228.07, found 228.2. ^1H NMR (300 MHz, $CDCl_3$) δ 5.12 (s, 2 H), 6.97–7.11 (m, 2 H), 7.31–7.48 (m, 4 H), 7.52–7.67 (m, 2 H).

4-((3-fluorobenzyl)oxy)benzonitrile (A3)

White solid. Yield: 80% (5.44 g); UPLC (purity: 99.2%): t_R = 3.98 min, LC-MS (ESI): *m/z* calcd for $C_{14}H_{10}FNO$ $(M+H)^+$ 228.07, found 228.1. ^1H NMR (300 MHz, $CDCl_3$) δ 5.11 (s, 2 H), 6.93–7.09 (m, 3 H), 7.09–7.43 (m, 3 H), 7.52–7.66 (m, 2 H).

4-((4-fluorobenzyl)oxy)benzonitrile (A4)

White solid. Yield: 77% (5.24 g); UPLC (purity: 98.4%): t_R = 3.97 min, LC-MS (ESI): *m/z* calcd for $C_{14}H_{10}FNO$ $(M+H)^+$ 228.07, found 228.1. ^1HNMR (300 MHz, $CDCl_3$) δ 5.12 (s, 2 H), 6.97–7.11 (m, 2 H), 7.31–7.48 (m, 4 H), 7.52–7.67 (m, 2 H).

4-((2-chlorobenzyl)oxy)benzonitrile (A5)

White solid. Yield: 74% (5.54 g); UPLC (purity: 98.8%): t_R = 3.96 min, LC-MS (ESI): *m/z* calcd for $C_{14}H_{10}ClNO$ $(M+H)^+$ 244.05, found 244.2. ^1H NMR (300 MHz, $CDCl_3$) δ 5.13 (s, 2 H), 6.98–7.13 (m, 2 H), 7.25–7.37 (m, 2 H), 7.39–7.54 (m, 2 H), 7.56–7.65 (m, 2 H).

4-((3-chlorobenzyl)oxy)benzonitrile (A6)

White solid. Yield: 81% (5.91 g); UPLC (purity: 99.5%): t_R = 3.95 min, LC-MS (ESI): *m/z* calcd for $C_{14}H_{10}ClNO$ $(M+H)^+$ 244.05, found 244.1. ^1H NMR (300 MHz, $CDCl_3$) δ 5.08 (s, 2 H), 6.82–7.07 (m, 2 H), 7.24–7.36 (m, 3 H), 7.41 (s, 1 H), 7.53–7.65 (m, 2 H).

4-((4-chlorobenzyl)oxy)benzonitrile (A7)

White solid. Yield: 75% (5.48 g); UPLC (purity: 99.7%): t_R = 3.94 min, LC-MS (ESI): *m/z* calcd for $C_{14}H_{10}ClNO$ $(M+H)^+$ 244.05, found 244.1. ^1H NMR (300 MHz, $CDCl_3$) δ 5.08 (s, 2 H), 6.89–7.10 (m, 2 H), 7.19–7.46 (m, 4 H), 7.49–7.76 (m, 2 H).

4-((2-(trifluoromethyl)benzyl)oxy)benzonitrile (A8)

White solid. Yield: 74% (6.14 g); UPLC (purity: 98.7%): t_R = 4.31 min, LC-MS (ESI): *m/z* calcd for $C_{15}H_{10}F_3NO$ $(M+H)^+$ 278.07, found 278.2. ^1H NMR (300 MHz, $CDCl_3$) δ 5.12 (s, 2 H,), 6.97–7.11 (m, 2 H), 7.31–7.48 (m, 4 H), 7.52–7.67 (m, 2 H).

4-((3-(trifluoromethyl)benzyl)oxy)benzonitrile (A9)

White solid. Yield: 76% (6.30 g); UPLC (purity: 99.8%): t_R = 4.32 min, LC-MS (ESI): *m/z* calcd for $C_{15}H_{10}F_3NO$ $(M+H)^+$ 278.07, found 278.2. ^1H NMR (300 MHz, $CDCl_3$) δ 5.16 (s, 2 H), 6.94–7.11 (m, 2 H), 7.26 (s, 1 H), 7.44–7.78 (m, 5 H).

4-((4-(trifluoromethyl)benzyl)oxy)benzonitrile (A10)

White solid. Yield: 68% (5.64 g); UPLC (purity: 98.9%): t_R = 4.32 min, LC-MS (ESI): *m/z* calcd for $C_{15}H_{10}F_3NO$ $(M+H)^+$ 278.07, found 278.2. ^1H NMR (300 MHz, $CDCl_3$) δ 5.18 (s, 2 H), 6.86–7.08 (m, 2 H), 7.27 (s, 1 H), 7.42–7.90 (m, 5 H)

4-((2-(trifluoromethoxy)benzyl)oxy)benzonitrile (A11)

White solid. Yield: 74% (6.49 g); UPLC (purity: 99.5%): t_R = 4.42 min, LC-MS (ESI): *m/z* calcd for $C_{15}H_{10}F_3NO_2$ $(M+H)^+$ 294.07, found 294.1. ^1H NMR (300 MHz, $CDCl_3$) δ 5.12 (s, 2 H,), 6.95–7.14 (m, 2 H), 7.32–7.48 (m, 4 H), 7.56–7.69 (m, 2 H).

4-((3-(trifluoromethoxy)benzyl)oxy)benzonitrile (A12)

White solid. Yield: 69% (6.05 g); UPLC (purity: 99.1%): t_R = 4.41 min, LC-MS (ESI): *m/z* calcd for $C_{15}H_{10}F_3NO_2$ $(M+H)^+$ 294.07, found 294.2. ^1H NMR (300 MHz, $CDCl_3$) δ 5.13 (s, 2 H,), 6.94–7.11 (m, 2 H), 7.30–7.51 (m, 4 H), 7.58–7.74 (m, 2 H).

4-((4-(trifluoromethoxy)benzyl)oxy)benzonitrile (A13)

White solid. Yield: 78% (6.84 g); UPLC (purity: 98.7%): t_R = 4.43 min, LC-MS (ESI): *m/z* calcd for $C_{15}H_{10}F_3NO_2$ $(M+H)^+$ 294.07, found 294.2. ^1H NMR (300 MHz, $CDCl_3$) δ 5.12 (s, 2 H,), 6.91–7.14 (m, 2 H), 7.29–7.54 (m, 4 H), 7.59–7.77 (m, 2 H).

4-((2-methylbenzyl)oxy)benzonitrile (A14)

White solid. Yield: 71% (4.74 g); UPLC (purity: 99.3%): t_R = 4.21 min, LC-MS (ESI): *m/z* calcd for $C_{15}H_{13}NO$ $(M+H)^+$ 224.10, found 224.2. ^1H NMR (300 MHz, $CDCl_3$) δ 2.35 (s, 3 H), 5.13 (s, 2 H,), 6.87–7.12 (m, 2 H), 7.29–7.50 (m, 3 H), 7.59–7.80 (m, 3 H).

4-((3-methylbenzyl)oxy)benzonitrile (A15)

White solid. Yield: 79% (5.27 g); UPLC (purity: 98.2%): t_R = 4.25 min, LC-MS (ESI): *m/z* calcd for $C_{15}H_{13}NO$ $(M+H)^+$ 224.10, found 224.2. ^1H NMR (300 MHz, $CDCl_3$) δ 2.36 (s, 3 H), 5.12 (s, 2 H,), 6.85–7.11 (m, 2 H), 7.26–7.47 (m, 4 H), 7.61–7.77 (m, 3 H).

4-((4-methylbenzyl)oxy)benzonitrile (A16)

White solid. Yield: 83% (5.48 g); UPLC (purity: 97.6%): t_R = 4.28 min, LC-MS (ESI): *m/z* calcd for $C_{15}H_{13}NO$ $(M+H)^+$ 224.10, found 224.2. ^1H NMR (300 MHz, $CDCl_3$) δ 2.35 (s, 3 H), 5.12 (s, 2 H,), 6.84–7.09 (m, 2 H), 7.25–7.49 (m, 3 H), 7.62–7.78 (m, 3 H).

2-(benzyloxy)benzonitrile (A17)

White solid. Yield: 85% (5.30 g); UPLC (purity: 99.4%): t_R = 3.81 min, LC-MS (ESI): *m/z* calcd for $C_{14}H_{11}NO$ $(M+H)^+$ 209.25, found 209.3.

3-(benzyloxy)benzonitrile (A18)

White solid. Yield: 81% (5.06 g); UPLC (purity: 98.7%): t_R = 3.87min, LC-MS (ESI): *m/z* calcd for $C_{14}H_{11}NO$ $(M+H)^+$ 209.25, found 209.3.

General synthetic procedure for (benzyloxy)phenyl)methanamine A19–A36

In a dry round-bottom flask placed in an ice bath under an inert gas atmosphere, a solution of 2.4 M $LiAlH_4$ (60 mmol, 3 eq) in THF was added. Then, a solution of the appropriate nitrile (20 mmol, 1 eq) in 20 ml THF was added dropwise. After 10 minutes, the reaction was continued at room temperature under an argon atmosphere. The reaction progress was monitored using HPLC. After one hour, the reaction mixture was cooled in an ice bath again, and sequentially, 4 ml of distilled water, 2 ml of 10% NaOH, and another 4 ml of distilled water were added. The mixture was stirred for 2 hours, then filtered through Celite, concentrated, and extracted with a DCM/H_2O. The organic layer was dried over anhydrous Na_2SO_4, and the solvent was evaporated under reduced pressure.

(4-(benzyloxy)phenyl)methanamine (A19)

White solid. Yield: 84% (3.58 g); UPLC (purity: 89.4%): t_R = 7.13 min, LC-MS (ESI): *m/z* calcd for $C_{14}H_{15}NO$ $(M+H)^+$ 214.12, found 214.2.

(4-((2-fluorobenzyl)oxy)phenyl)methanamine (A20)

White solid. Yield: 81% (3.74 g); UPLC (purity: 99.1%): t_R = 6.83 min, LC-MS (ESI): *m/z* calcd for $C_{14}H_{14}FNO$ $(M+H)^+$ 232.11, found 232.2.

(4-((3-fluorobenzyl)oxy)phenyl)methanamine (A21)

White solid. Yield: 77% (3.55 g); UPLC (purity: 98.4%): t_R = 6.94 min, LC-MS (ESI): *m/z* calcd for $C_{14}H_{14}FNO$ $(M+H)^+$ 232.11, found 232.2.

(4-((4-fluorobenzyl)oxy)phenyl)methanamine (A22)

White solid. Yield: 69% (3.18 g); UPLC (purity: 97.2%): t_R = 6.96 min, LC-MS (ESI): *m/z* calcd for $C_{14}H_{14}FNO$ $(M+H)^+$ 232.11, found 232.2.

(4-((2-chlorobenzyl)oxy)phenyl)methanamine (A23)

White solid. Yield: 78% (3.85 g); UPLC (purity: 96.5%): t_R = 7.73 min, LC-MS (ESI): *m/z* calcd for $C_{14}H_{14}ClNO$ $(M+H)^+$ 248.08, found 248.1.

(4-((3-chlorobenzyl)oxy)phenyl)methanamine (A24)

White solid. Yield: 72% (3.55 g); UPLC (purity: 98.5%): t_R = 7.75 min, LC-MS (ESI): *m/z* calcd for $C_{14}H_{14}ClNO$ $(M+H)^+$ 248.08, found 248.2.

(4-((4-chlorobenzyl)oxy)phenyl)methanamine (A25)

White solid. Yield: 83% (4.10 g); UPLC (purity: 97.6%): t_R = 7.76 min, LC-MS (ESI): *m/z* calcd for $C_{14}H_{14}ClNO$ $(M+H)^+$ 248.08, found 248.2.

(4-((2-(trifluoromethyl)benzyl)oxy)phenyl)methanamine (A26)

White solid. Yield: 78% (4.38 g); UPLC (purity: 99.3%): t_R = 7.82 min, LC-MS (ESI): *m/z* calcd for $C_{15}H_{14}F_3NO$ $(M+H)^+$ 282.10, found 282.2.

(4-((3-(trifluoromethyl)benzyl)oxy)phenyl)methanamine (A27)

White solid. Yield: 82% (4.60 g); UPLC (purity: 97.4%): t_R = 6.74 min, LC-MS (ESI): *m/z* calcd for $C_{15}H_{14}F_3NO$ $(M+H)^+$ 282.10, found 282.2.

(4-((4-(trifluoromethyl)benzyl)oxy)phenyl)methanamine (A28)

White solid. Yield: 68% (3.88 g); UPLC (purity: 98.5%): t_R = 7.83 min, LC-MS (ESI): *m/z* calcd for $C_{15}H_{14}F_3NO$ $(M+H)^+$ 282.10, found 282.2.

(4-((2-(trifluoromethoxy)benzyl)oxy)phenyl)methanamine (A29)

White solid. Yield: 81% (4.81 g); UPLC (purity: 95.4%): t_R = 7.89 min, LC-MS (ESI): *m/z* calcd for $C_{15}H_{14}F_3NO_2$ $(M+H)^+$ 298.10, found 298.2.

(4-((3-(trifluoromethoxy)benzyl)oxy)phenyl)methanamine (A30)

White solid. Yield: 77% (4.57 g); UPLC (purity: 96.2%): t_R = 7.71 min, LC-MS (ESI): *m/z* calcd for $C_{15}H_{14}F_3NO_2$ $(M+H)^+$ 298.10, found 298.2.

(4-((4-(trifluoromethoxy)benzyl)oxy)phenyl)methanamine (A31)

White solid. Yield: 83% (4.93 g); UPLC (purity: 100%): t_R = 7.87 min, LC-MS (ESI): *m/z* calcd for $C_{15}H_{14}F_3NO_2$ $(M+H)^+$ 298.10, found 298.2.

(4-((2-methylbenzyl)oxy)phenyl)methanamine (A32)

White solid. Yield: 80% (3.63 g); UPLC (purity: 98.7%): t_R = 7.10 min, LC-MS (ESI): *m/z* calcd for $C_{15}H_{17}NO$ $(M+H)^+$ 228.13, found 228.2.

(4-((3-methylbenzyl)oxy)phenyl)methanamine (A33)

White solid. Yield: 84% (3.81 g); UPLC (purity: 96.8%): t_R = 7.08 min, LC-MS (ESI): *m/z* calcd for $C_{15}H_{17}NO$ $(M+H)^+$ 228.13, found 228.2.

(4-((4-methylbenzyl)oxy)phenyl)methanamine (A34)

White solid. Yield: 82% (3.72 g); UPLC (purity: 97.6%): t_R = 7.12 min, LC-MS (ESI): *m/z* calcd for $C_{15}H_{17}NO$ $(M+H)^+$ 228.13, found 228.2.

(2-(benzyloxy)phenyl)methanamine (A35)

White solid. Yield: 81% (3.49 g); UPLC (purity: 99.1%): t_R = 7.02 min, LC-MS (ESI): *m/z* calcd for $C_{14}H_{15}NO$ $(M+H)^+$ 214.12, found 214.2.

(3-(benzyloxy)phenyl)methanamine (A36)

White solid. Yield: 78% (3.42 g); UPLC (purity: 98.7%): t_R = 7.08 min, LC-MS (ESI): *m/z* calcd for $C_{14}H_{15}NO$ $(M+H)^+$ 214.12, found 214.2.

Table S2. Inhibitory effects of obtained compounds on MAO-B.

**Compound**	% inhibition MAO-B (1 µM) [a]	IC_{50} ± SEM [nM] [b]
**5**	0	-
**6**	0	-
**7**	0	-
**8**	0	-
**9**	0	-
**10**	0	-
**11**	0	-
**12**	0	-
**13**	0	-
**14**	0	-
**15**	0	-
**16**	31	-
**17**	0	-
**18**	0	-
**19**	0	-
**20**	0	-
**21**	0	-
**22**	0	-
**23**	0	-
**36**	0	-
**37**	0	-
**38**	2	-
**39**	0	-
**Positive control (Safinamide)**	100	8 ± 1
**Positive control (Rasagiline)**	100	25 ± 6

[a] % of inhibition at 1 µM; mean values of two independent experiments.[b] IC_{50} values were determined by fluorometric method using human recombinant MAO-B and rasagiline [1 µM] as a positive control.

Table S3. The general information of radioligand binding/functional assays.

**Binding studies**	Ref.
Na^{+} channel (site 2)	[1]
Ca^{2+}channel (L, dihydropyridine site) (antagonist radioligand)	[2]
Ca^{2+}channel (L, diltiazem site) (antagonist radioligand)	[3]
Ca^{2+}channel (L, verapamil site) (antagonist radioligand)	[4]
N-type Ca^{2+} [^{3}H] Gabapentin binding (antagonist radioligand)	[5]
GABA transporter (antagonist radioligand)	[6]
GLYT1 (glycine transporter binding) (antagonist radioligand)	[7]
Potassium channel (hERG)	[8]
**Functional studies**	
$Cav_{1.2}$ (L-type) *(h)* calcium ion channel cell based antagonist calcium flux assay	[9,10]

Assays were performed commercially in Eurofins Cerep SA (Celle l'Evescault, France) and Eurofins Panlabs Discovery Services Taiwan, Ltd. (New Taipei City, Taiwan), using procedures describe elsewhere:

References

1. Brown, G.B. 3H-Batrachotoxinin-A Benzoate Binding to Voltage-Sensitive Sodium Channels: Inhibition by the Channel Blockers Tetrodotoxin and Saxitoxin. *J. Neurosci.* **1986**, *6*, 2064–2070.
2. Gould, R.J.; Murphy, K.M.; Snyder, S.H. [3H]Nitrendipine-Labeled Calcium Channels Discriminate Inorganic Calcium Agonists and Antagonists. *Proc. Natl. Acad. Sci. USA* **1982**, *79*, 3656–3660, doi:10.1073/pnas.79.11.3656.
3. Schoemaker, H.; Langer, S.Z. [3H]Diltiazem Binding to Calcium Channel Antagonists Recognition Sites in Rat Cerebral Cortex. *Eur. J. Pharmacol.* **1985**, *111*, 273–277, doi:10.1016/0014-2999(85)90768-x.
4. Reynolds, I.J.; Snowman, A.M.; Snyder, S.H. (-)-[3H] Desmethoxyverapamil Labels Multiple Calcium Channel Modulator Receptors in Brain and Skeletal Muscle Membranes: Differentiation by Temperature and Dihydropyridines. *J. Pharmacol. Exp. Ther* **1986**, *237*, 731–738.
5. Marais, E.; Klugbauer, N.; Hofmann, F. Calcium Channel Alpha(2)Delta Subunits-Structure and Gabapentin Binding. *Mol. Pharmacol.* **2001**, *59*, 1243–1248, doi:10.1124/mol.59.5.1243.
6. Shank, R.P.; Baldy, W.J.; Mattucci, L.C.; Villani, F.J. Ion and Temperature Effects on the Binding of γ-Aminobutyrate to Its Receptors and the High-Affinity Transport System. *J. Neurochem.* **1990**, *54*, 2007–2015, doi:10.1111/j.1471-4159.1990.tb04905.x.
7. Atkinson, B.N.; Bell, S.C.; Vivo, M.D.; Kowalski, L.R.; Lechner, S.M.; Ognyanov, V.I.; Tham, C.-S.; Tsai, C.; Jia, J.; Ashton, D.; et al. ALX 5407: A Potent, Selective Inhibitor of the hGlyT1 Glycine Transporter. *Mol. Pharmacol.* **2001**, *60*, 1414–1420, doi:10.1124/mol.60.6.1414.
8. Huang, X.-P.; Mangano, T.; Hufeisen, S.; Setola, V.; Roth, B.L. Identification of Human Ether-à-Go-Go Related Gene Modulators by Three Screening Platforms in an Academic Drug-Discovery Setting. *Assay Drug. Dev. Technol.* **2010**, *8*, 727–742, doi:10.1089/adt.2010.0331.
9. Sirenko, O.; Crittenden, C.; Callamaras, N.; Hesley, J.; Chen, Y.-W.; Funes, C.; Rusyn, I.; Anson, B.; Cromwell, E.F. Multiparameter in Vitro Assessment of Compound Effects on Cardiomyocyte Physiology Using iPSC Cells. *J. Biomol. Screen.* **2013**, *18*, 39–53, doi:10.1177/1087057112457590.
10. Xia, M.; Imredy, J.P.; Koblan, K.S.; Bennett, P.; Connolly, T.M. State-Dependent Inhibition of L-Type Calcium Channels: Cell-Based Assay in High-Throughput Format. *Anal. Biochem.* **2004**, *327*, 74–81, doi:10.1016/j.ab.2004.01.003.

UPLC/HRMS traces for target final compounds

***N*-(4-(benzyloxy)benzyl)-2-(2,5-dioxopyrrolidin-1-yl)propanamide (5)**

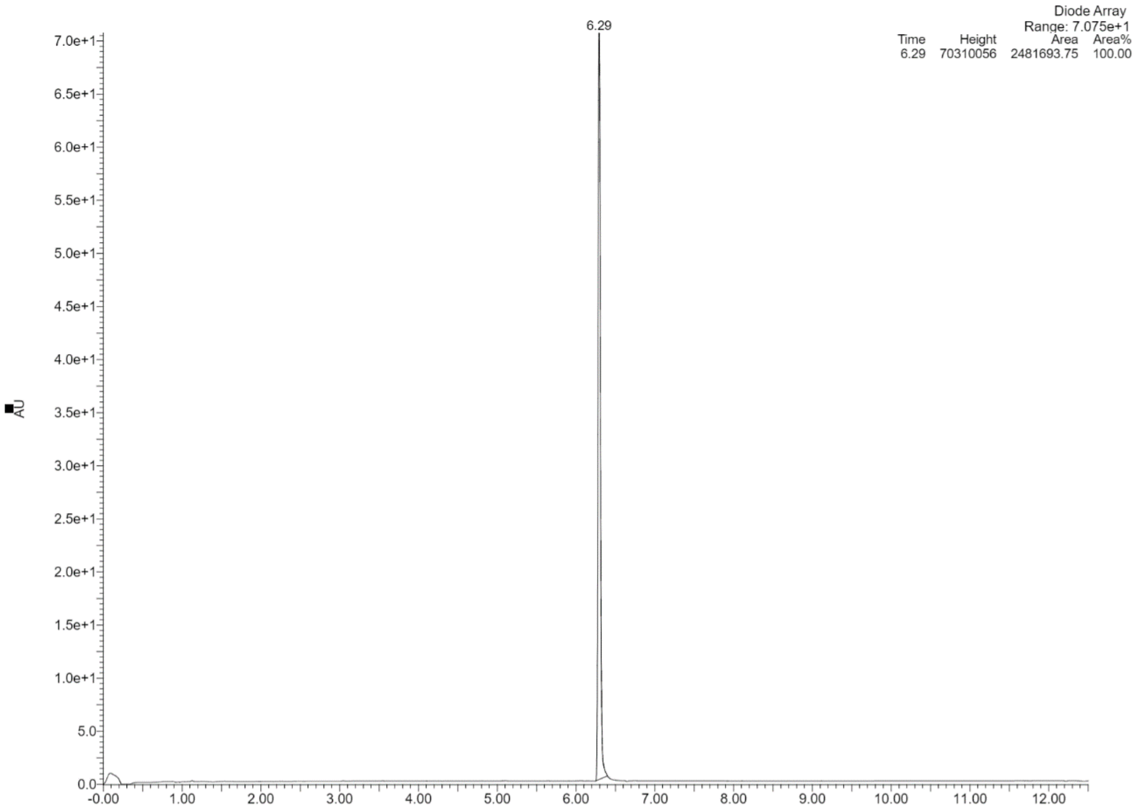

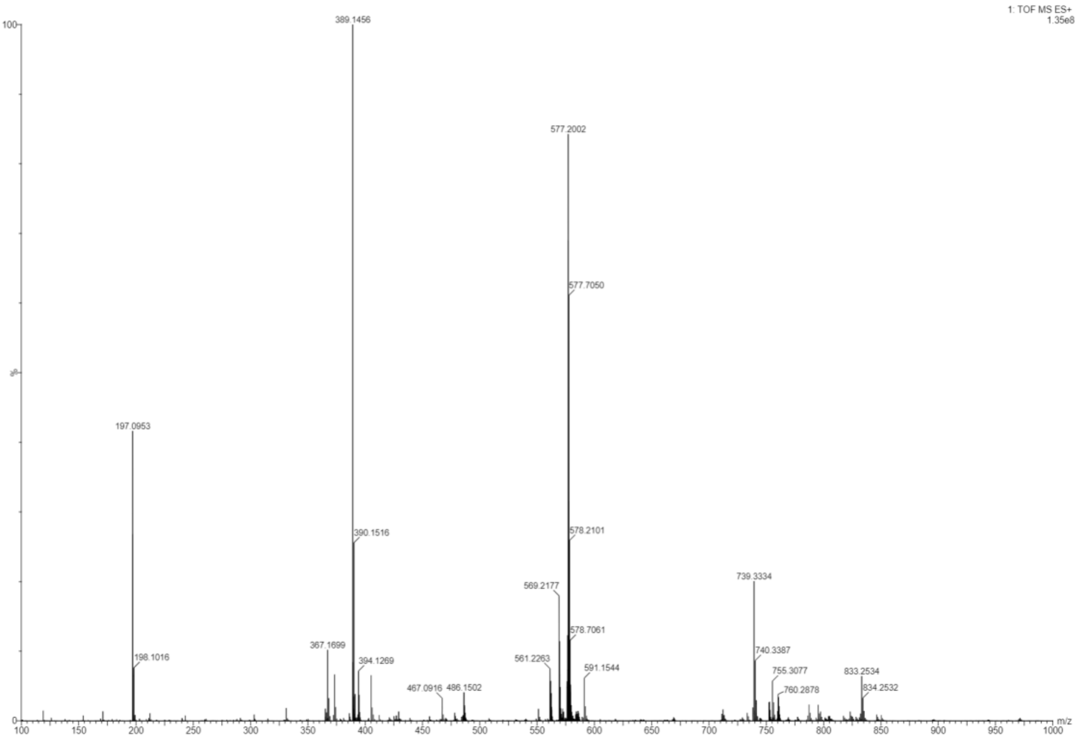

2-(2,5-dioxopyrrolidin-1-yl)-*N*-(4-((2-fluorobenzyl)oxy)benzyl)propanamide (6)

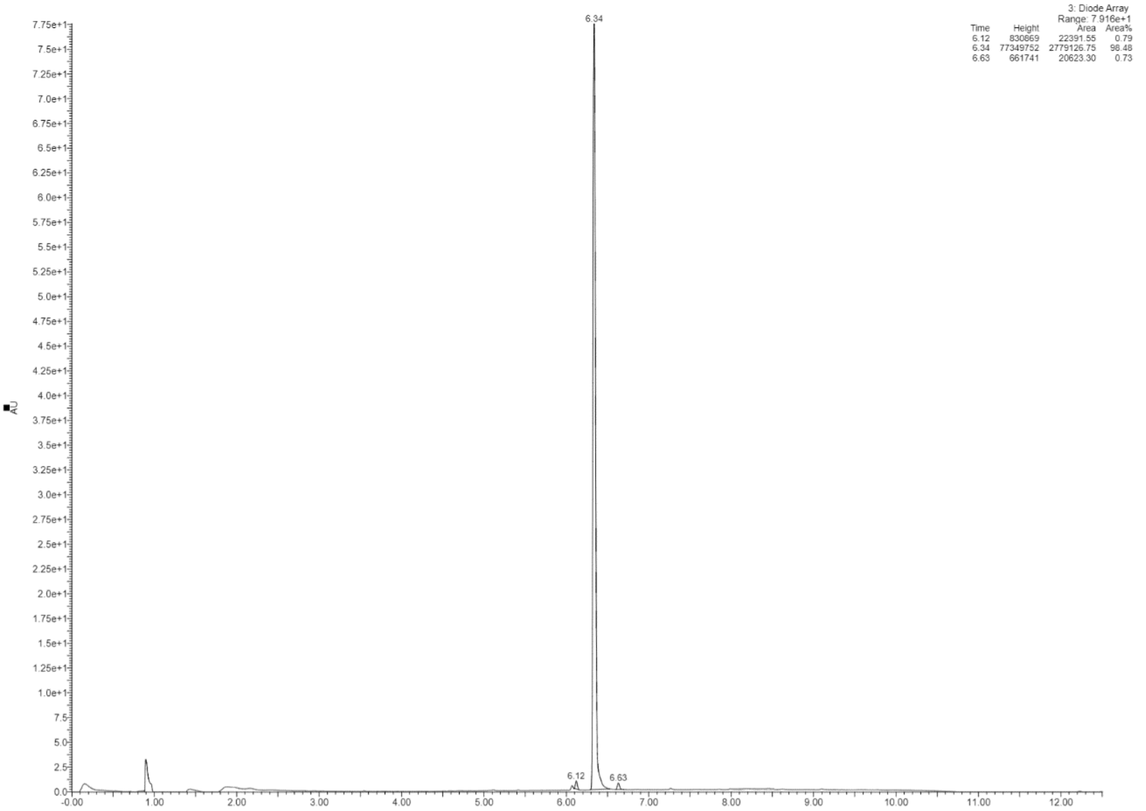

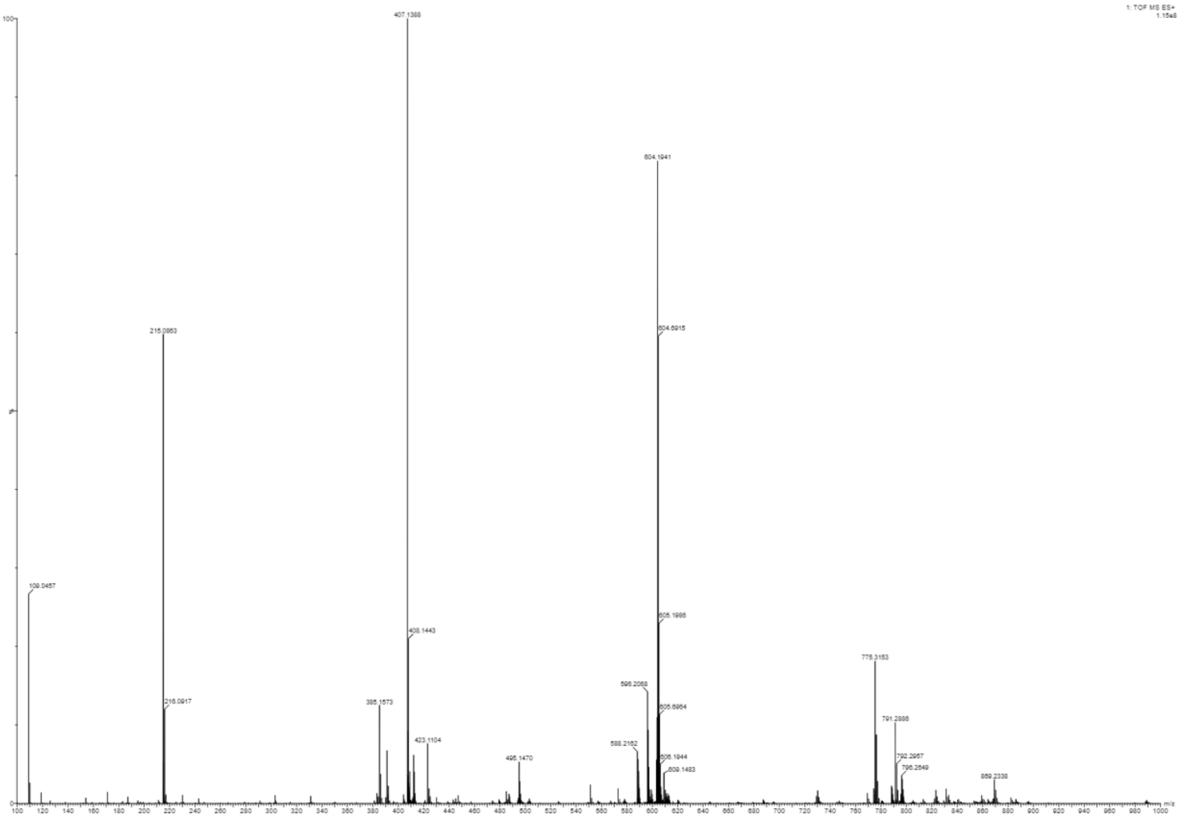

2-(2,5-dioxopyrrolidin-1-yl)-*N*-(4-((3-fluorobenzyl)oxy)benzyl)propanamide (7)

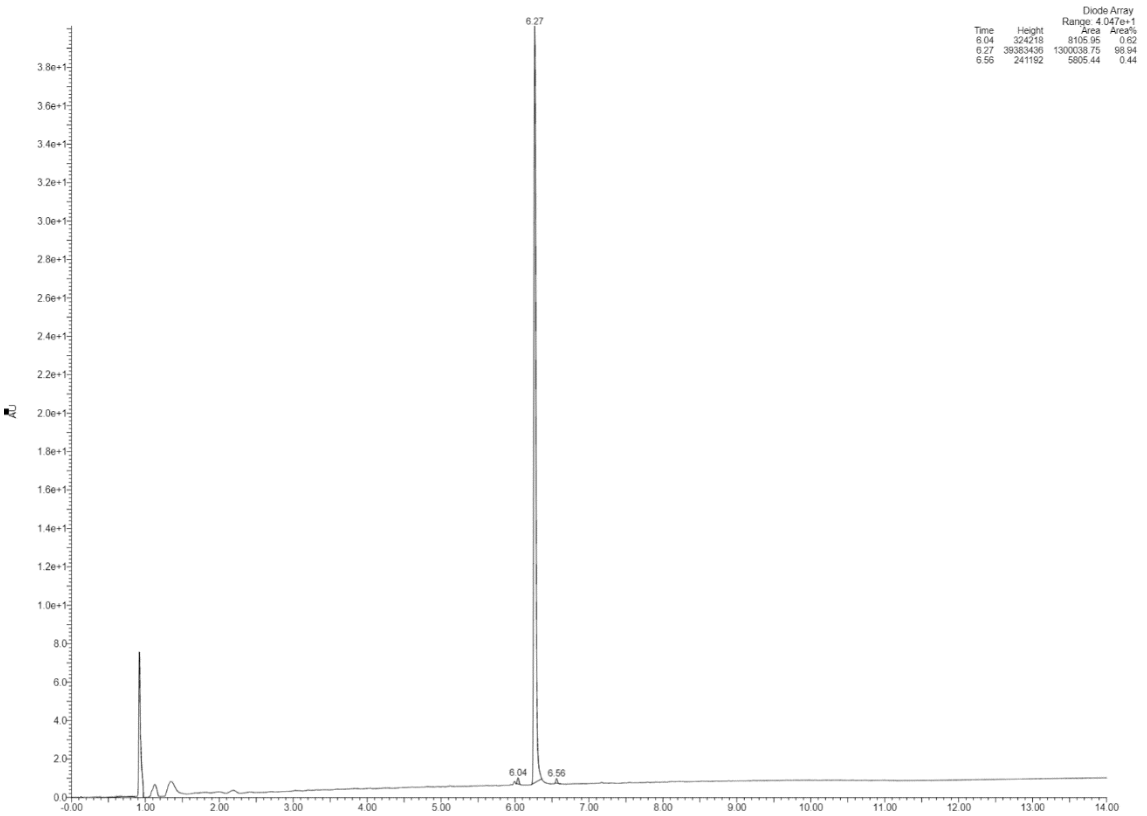

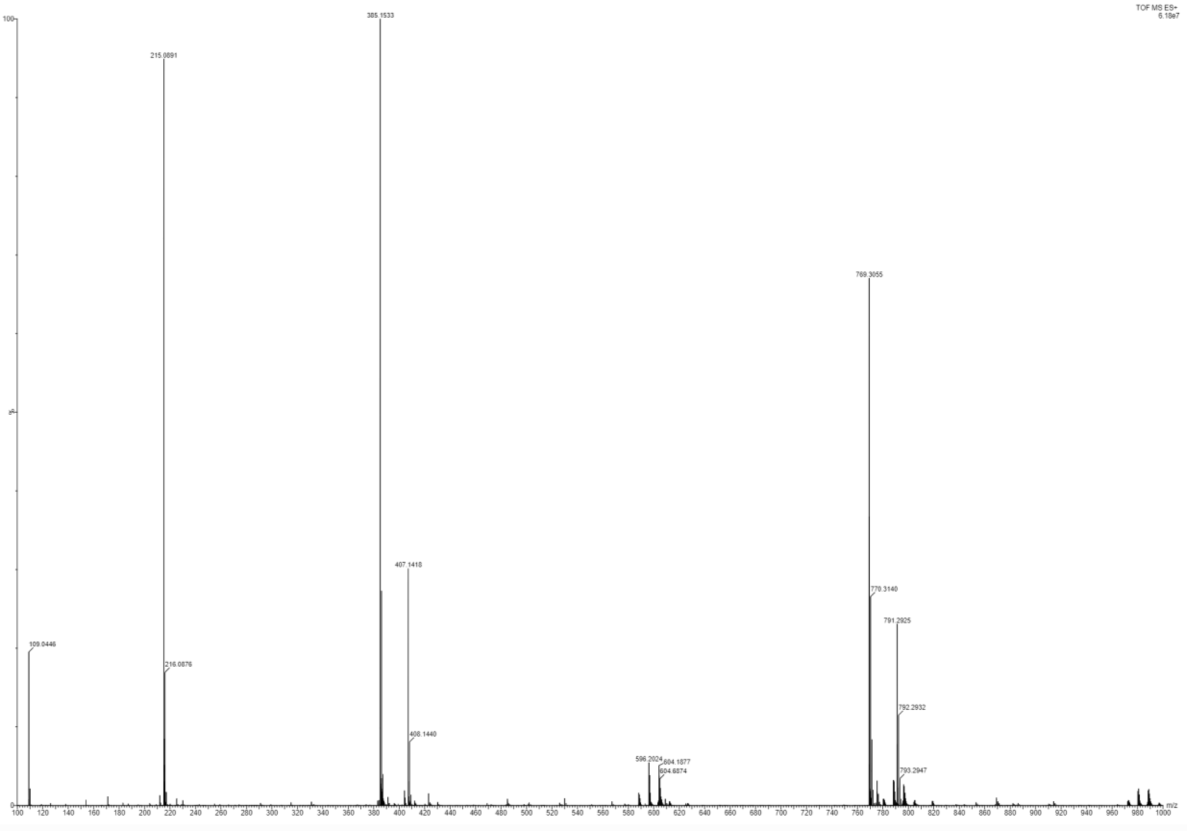

2-(2,5-dioxopyrrolidin-1-yl)-*N*-(4-((4-fluorobenzyl)oxy)benzyl)propanamide (8)

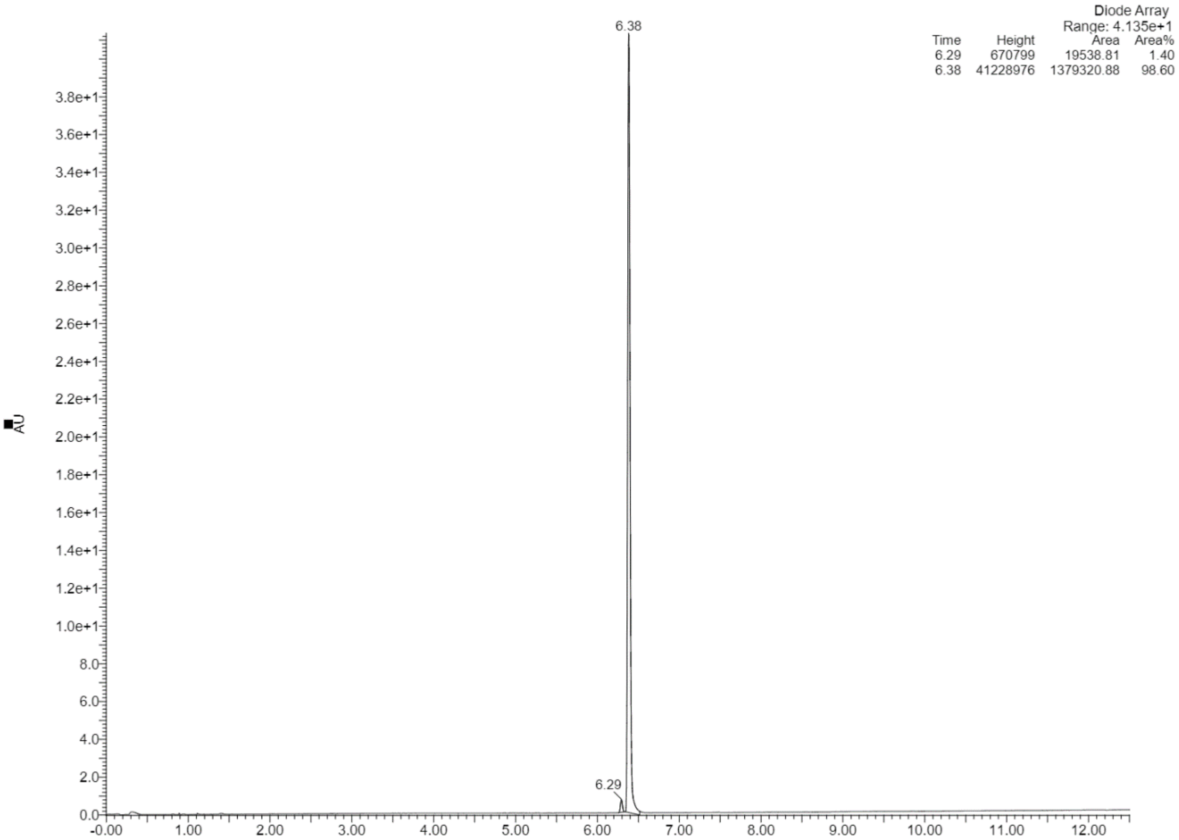

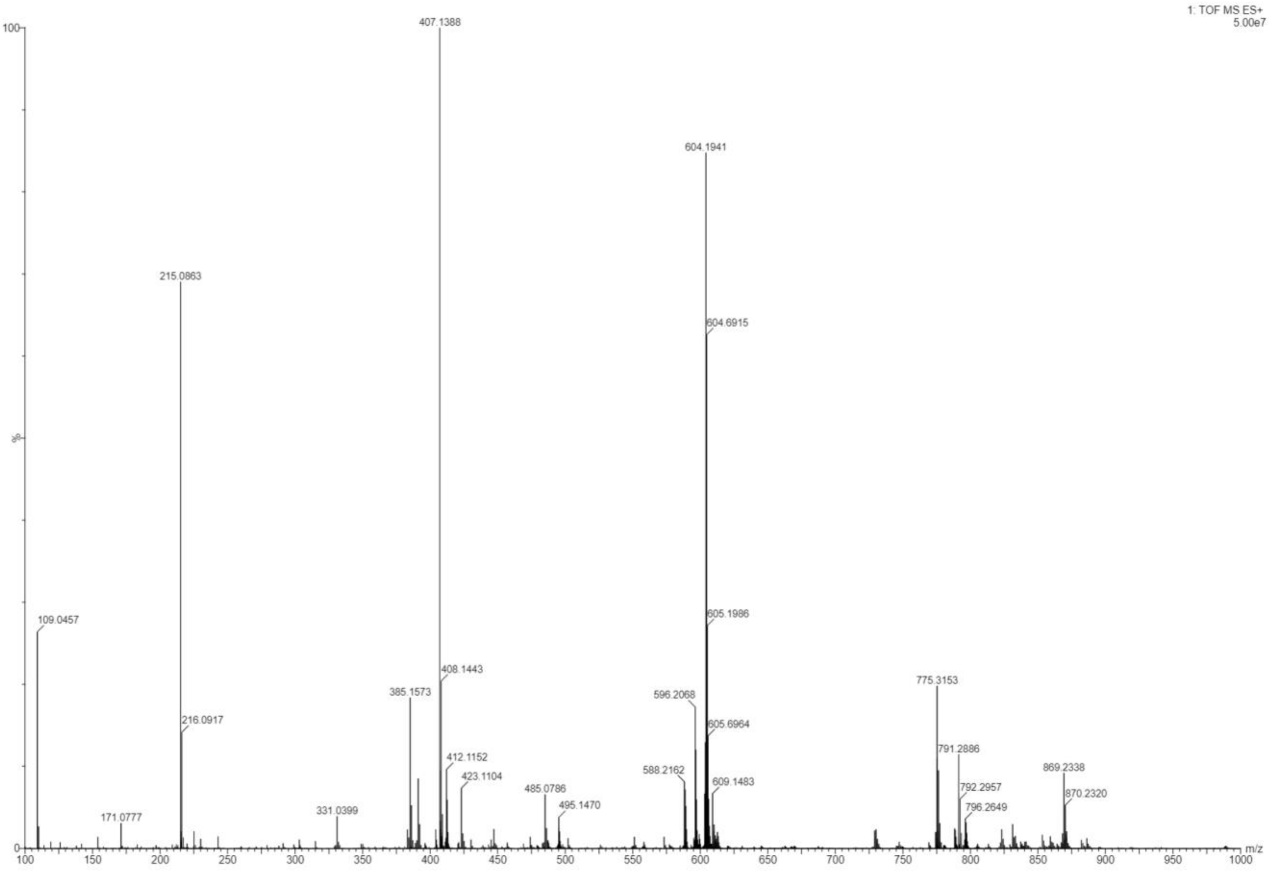

N-(4-((2-chlorobenzyl)oxy)benzyl)-2-(2,5-dioxopyrrolidin-1-yl)propanamide (9)

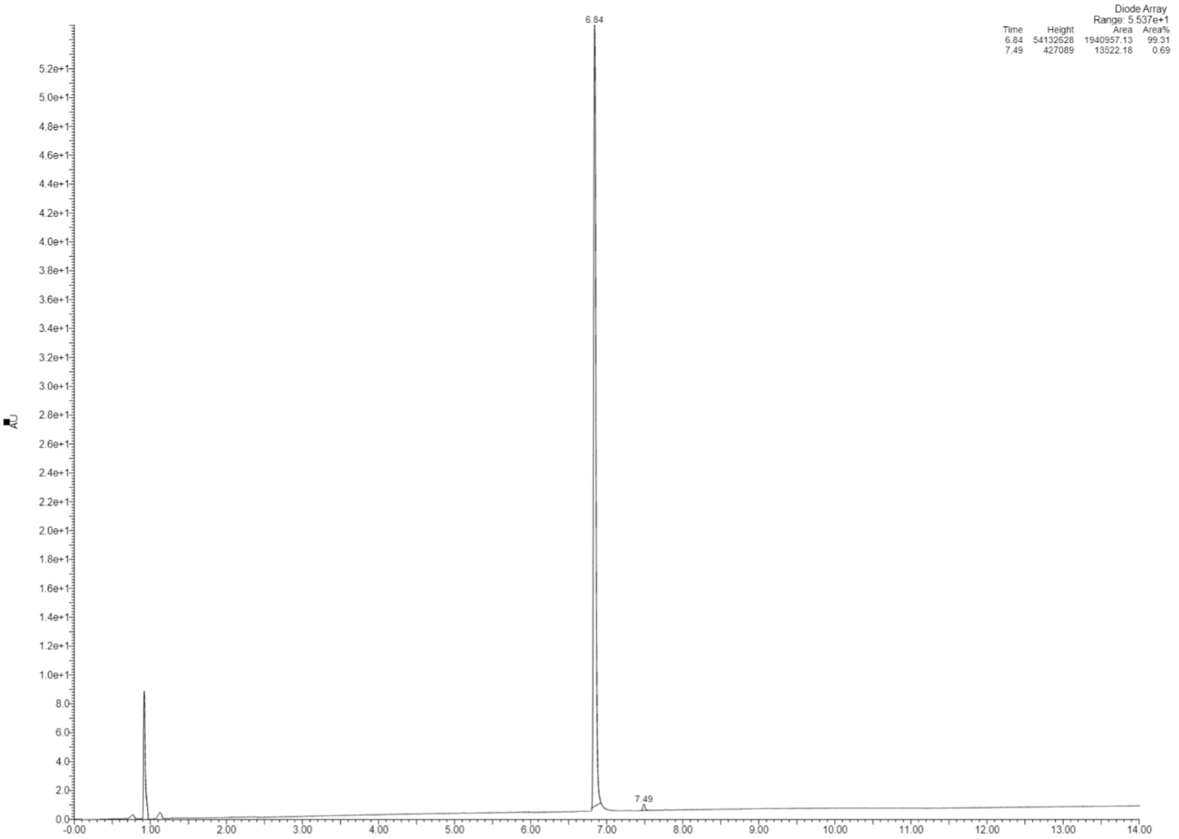

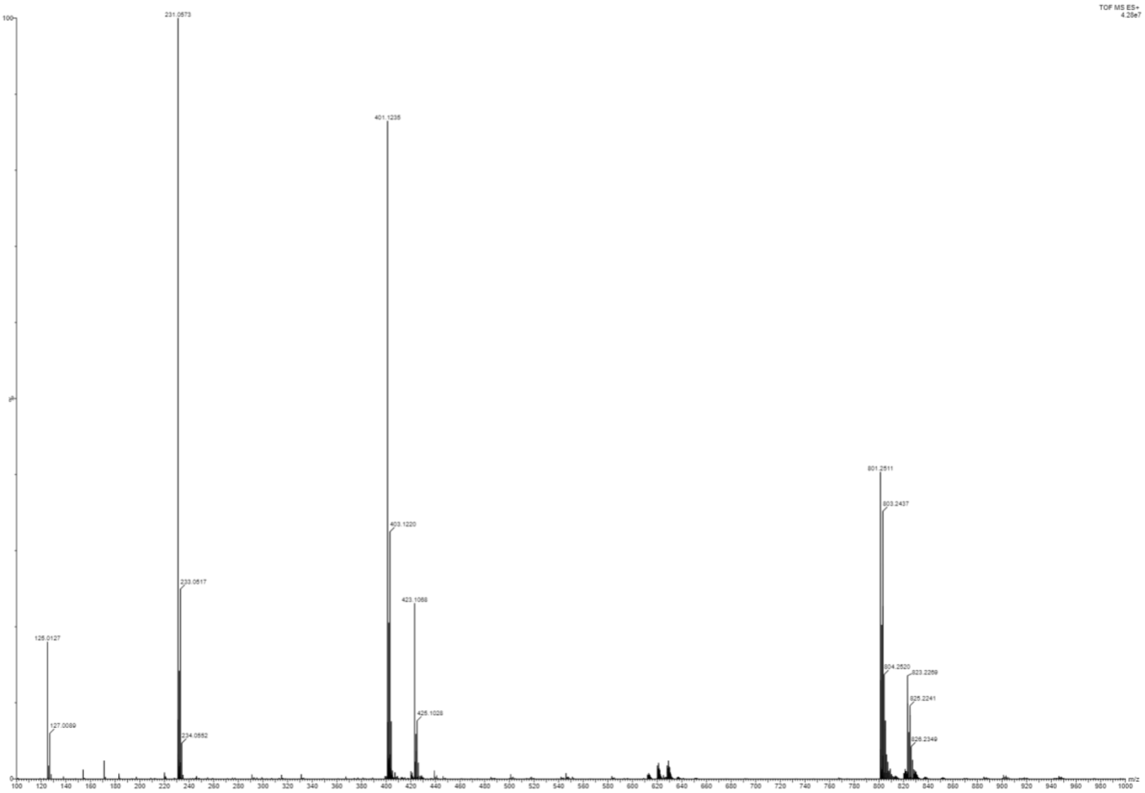

_N_-(4-((3-chlorobenzyl)oxy)benzyl)-2-(2,5-dioxopyrrolidin-1-yl)propanamide (10)

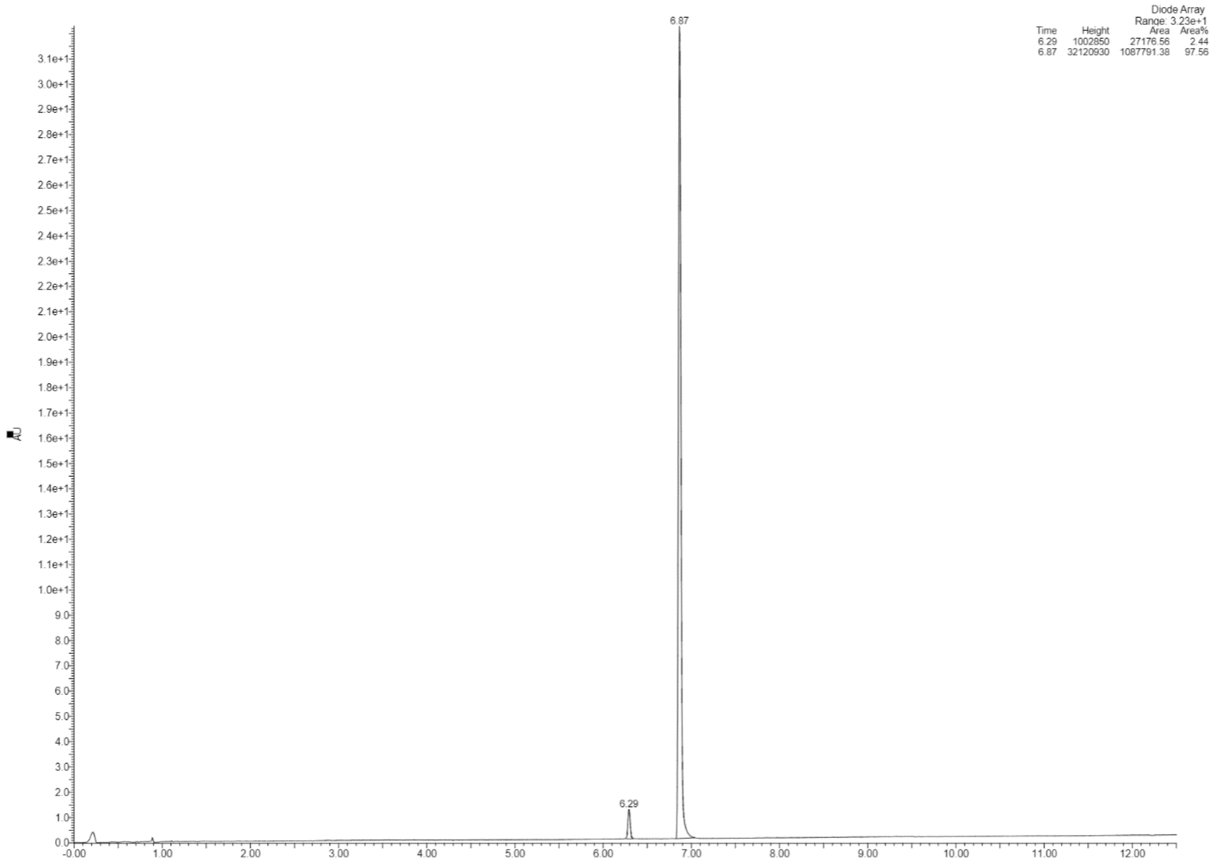

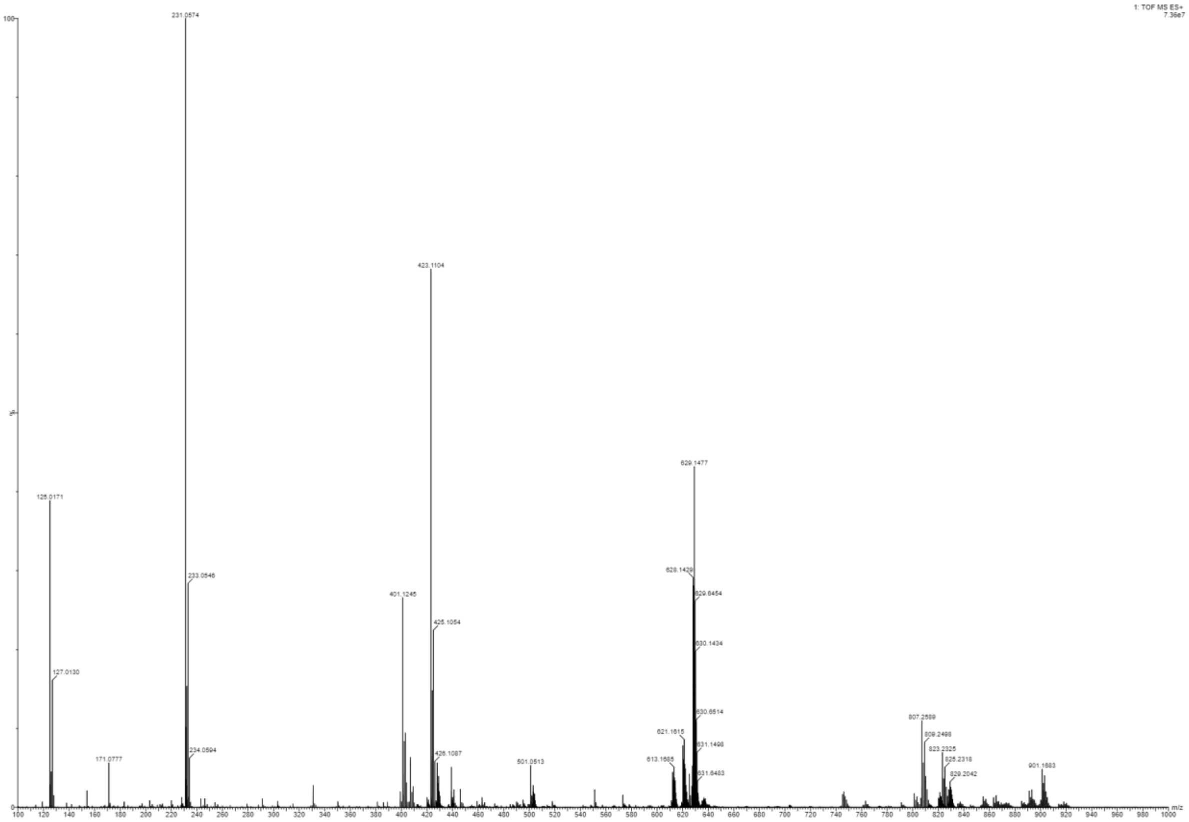

***N*-(4-((4-chlorobenzyl)oxy)benzyl)-2-(2,5-dioxopyrrolidin-1-yl)propanamide (11)**

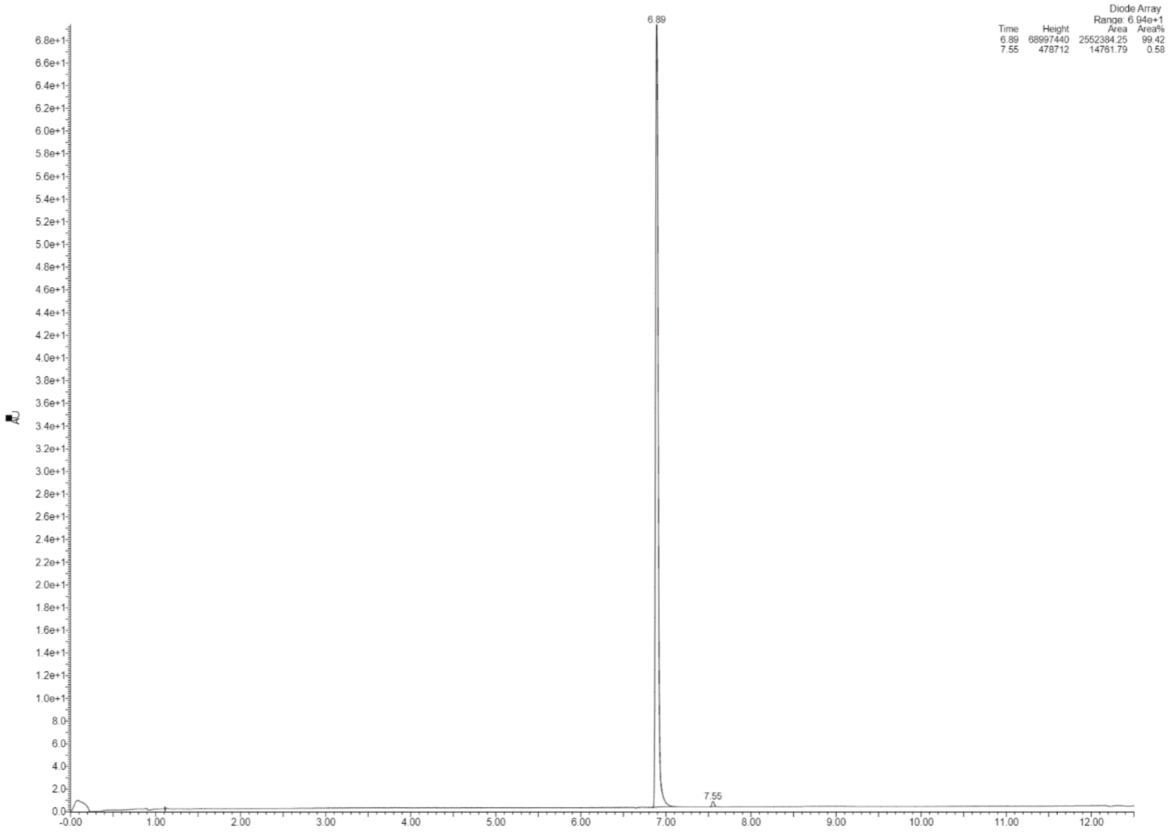

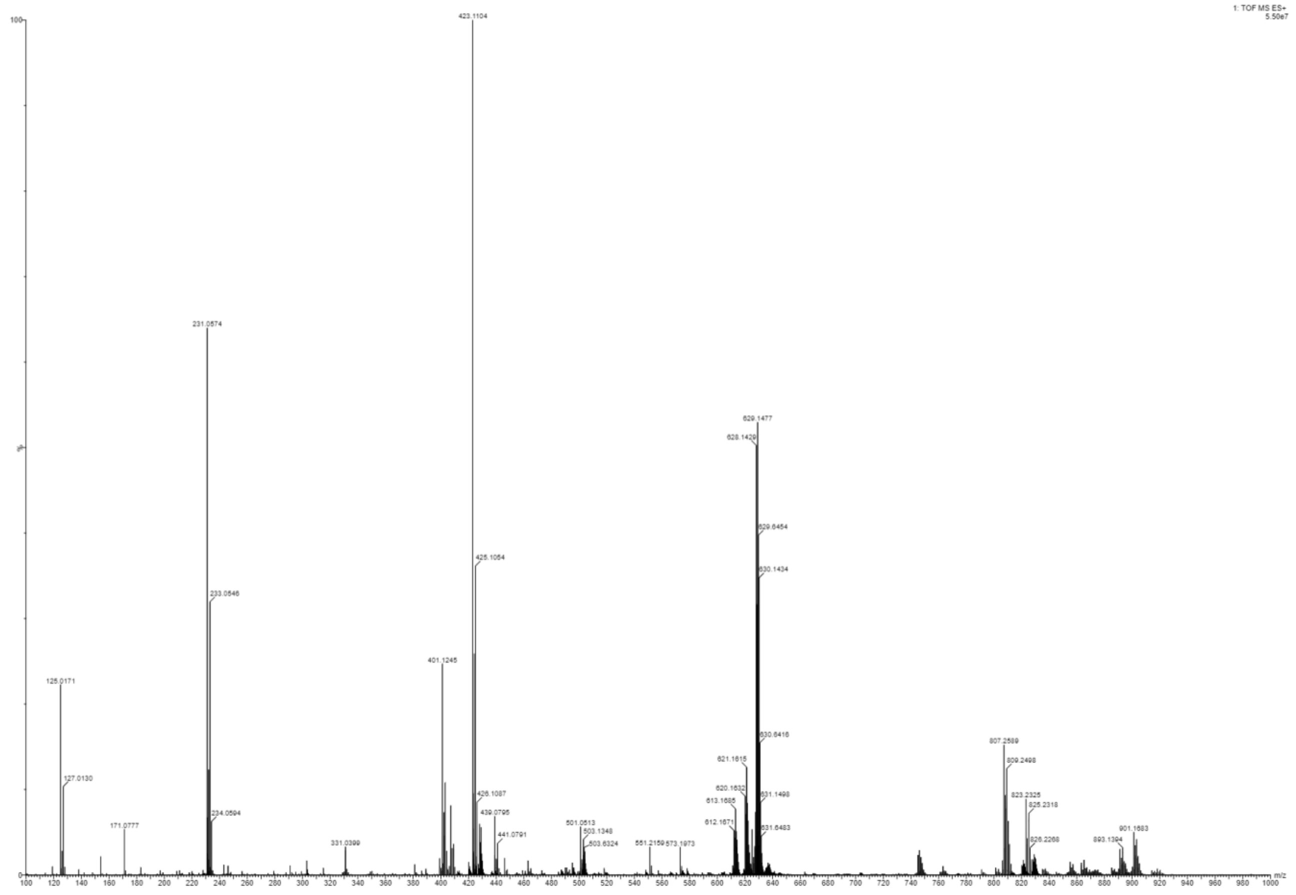

2-(2,5-dioxopyrrolidin-1-yl)-*N*-(4-((2-(trifluoromethyl)benzyl)oxy)benzyl)propanamide (12)

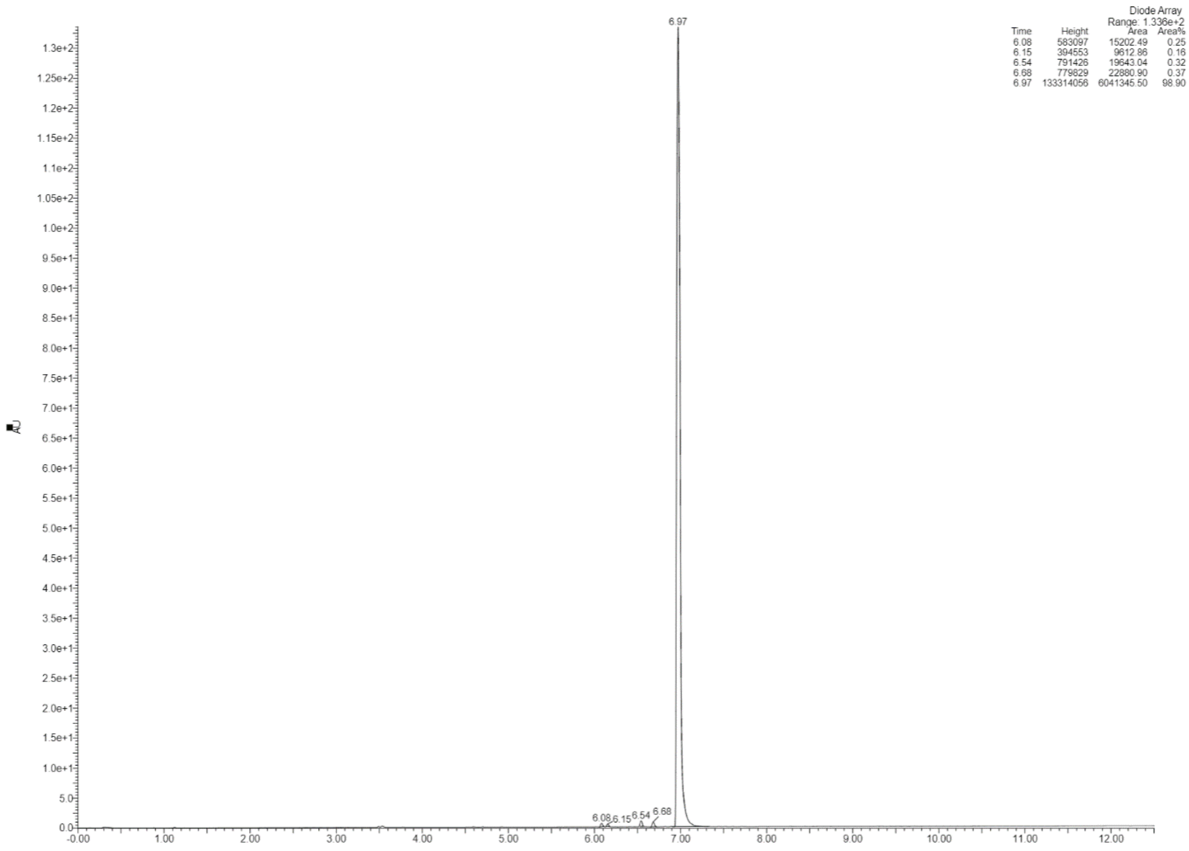

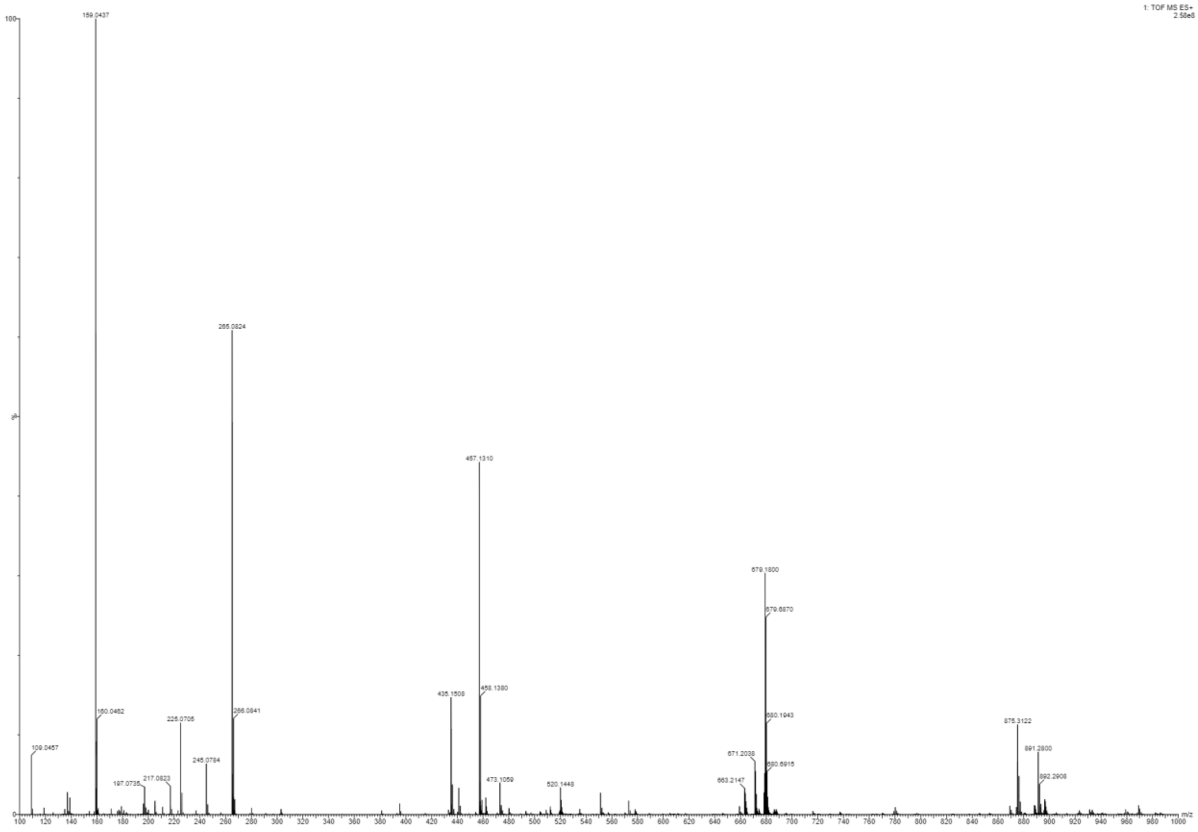

2-(2,5-dioxopyrrolidin-1-yl)-*N*-(4-((3-(trifluoromethyl)benzyl)oxy)benzyl)propenamide (13)

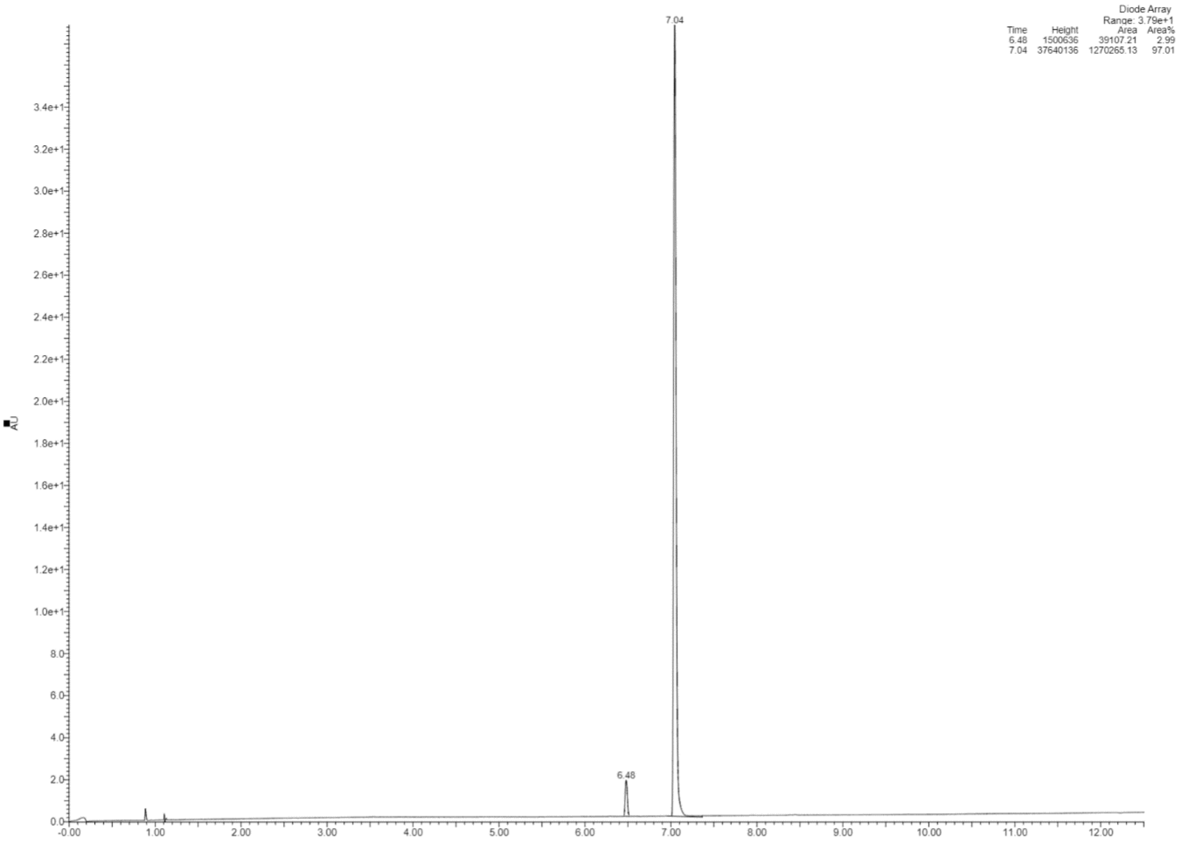

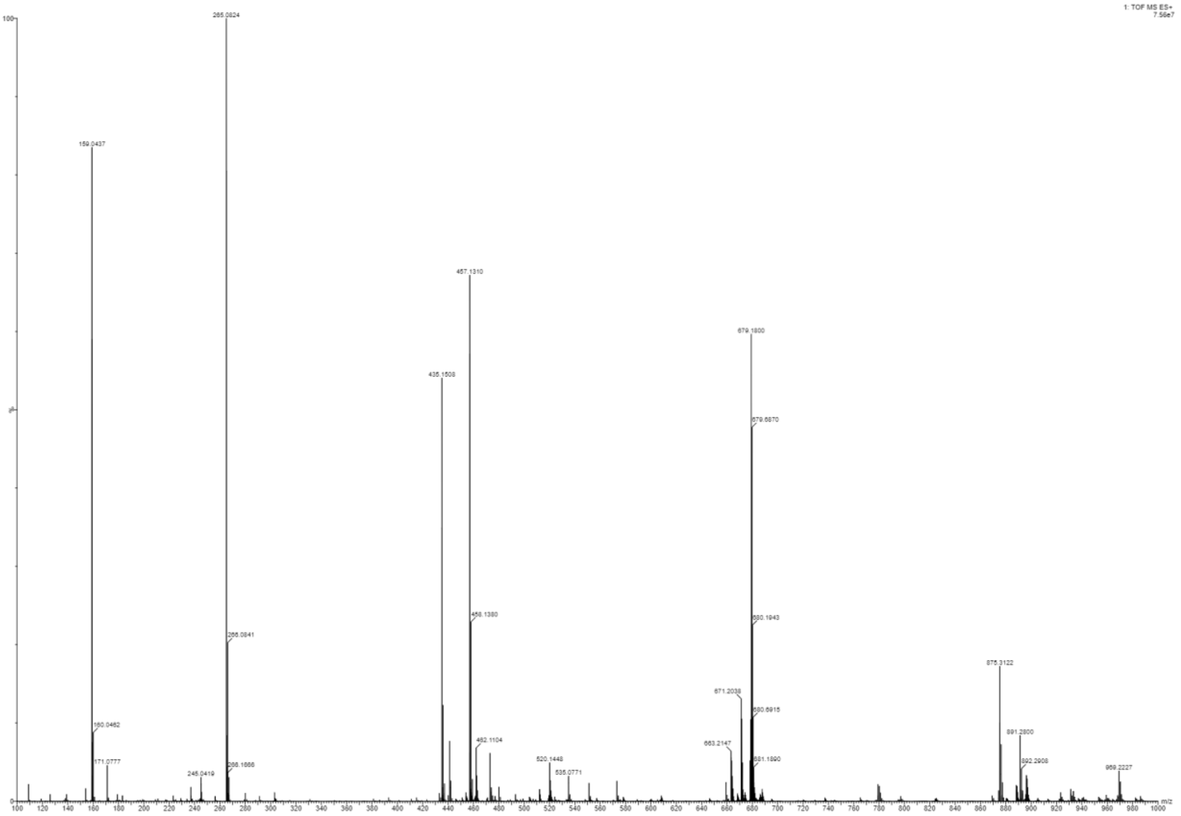

2-(2,5-dioxopyrrolidin-1-yl)-*N*-(4-((4-(trifluoromethyl)benzyl)oxy)benzyl)propenamide (14)

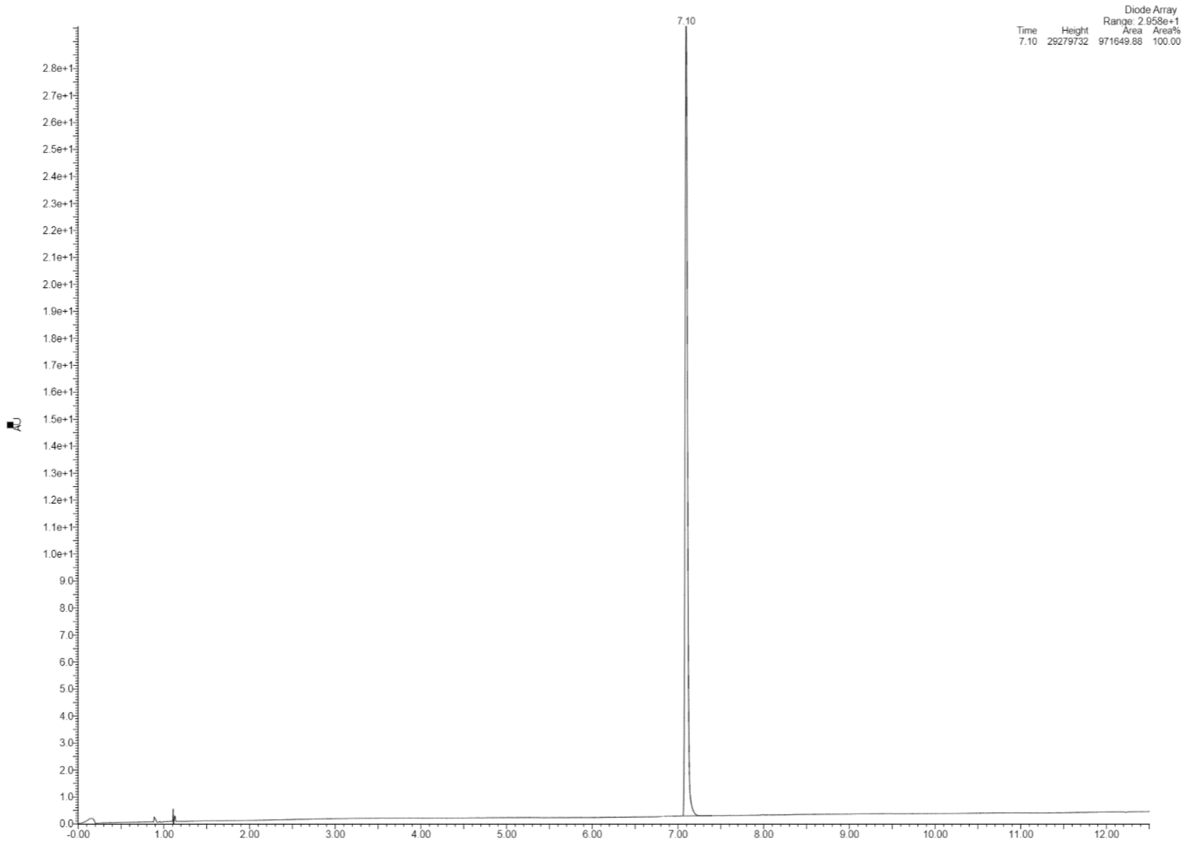

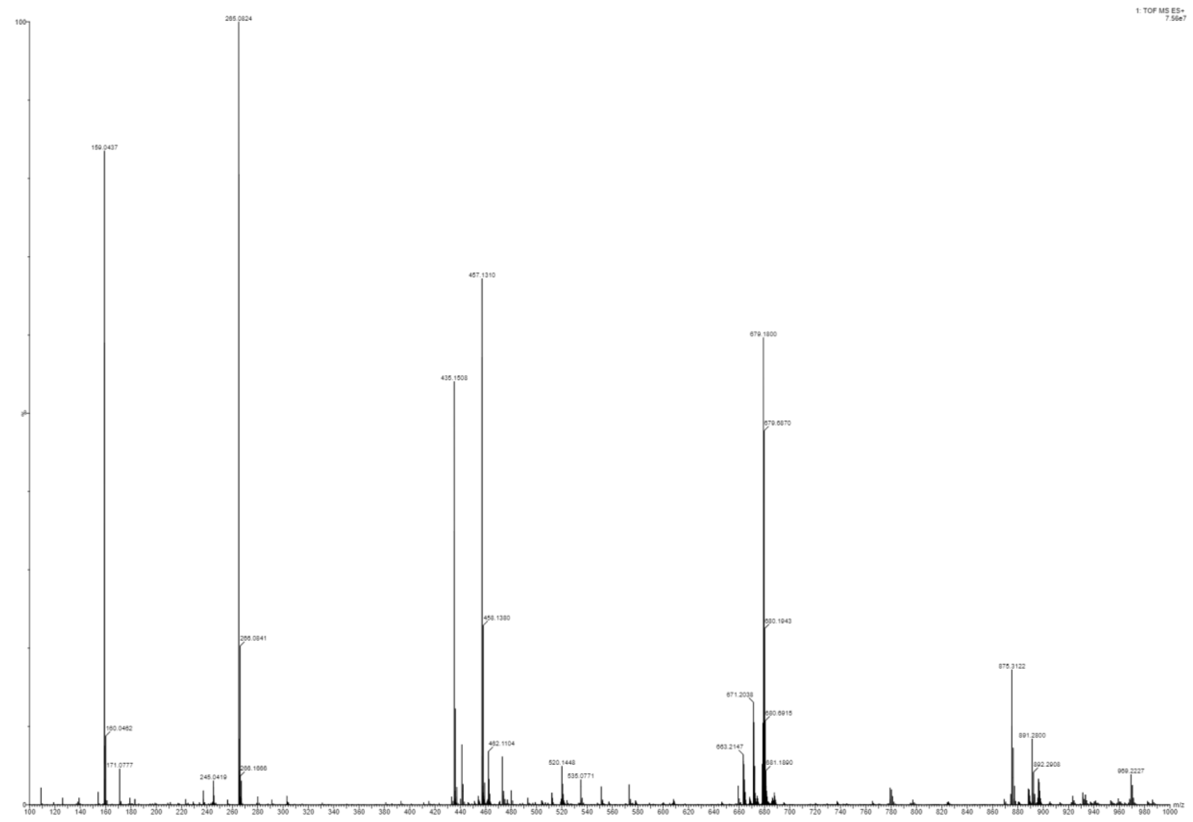

2-(2,5-dioxopyrrolidin-1-yl)-*N*-(4-((2-(trifluoromethoxy)benzyl)oxy)benzyl)propanamide (15)

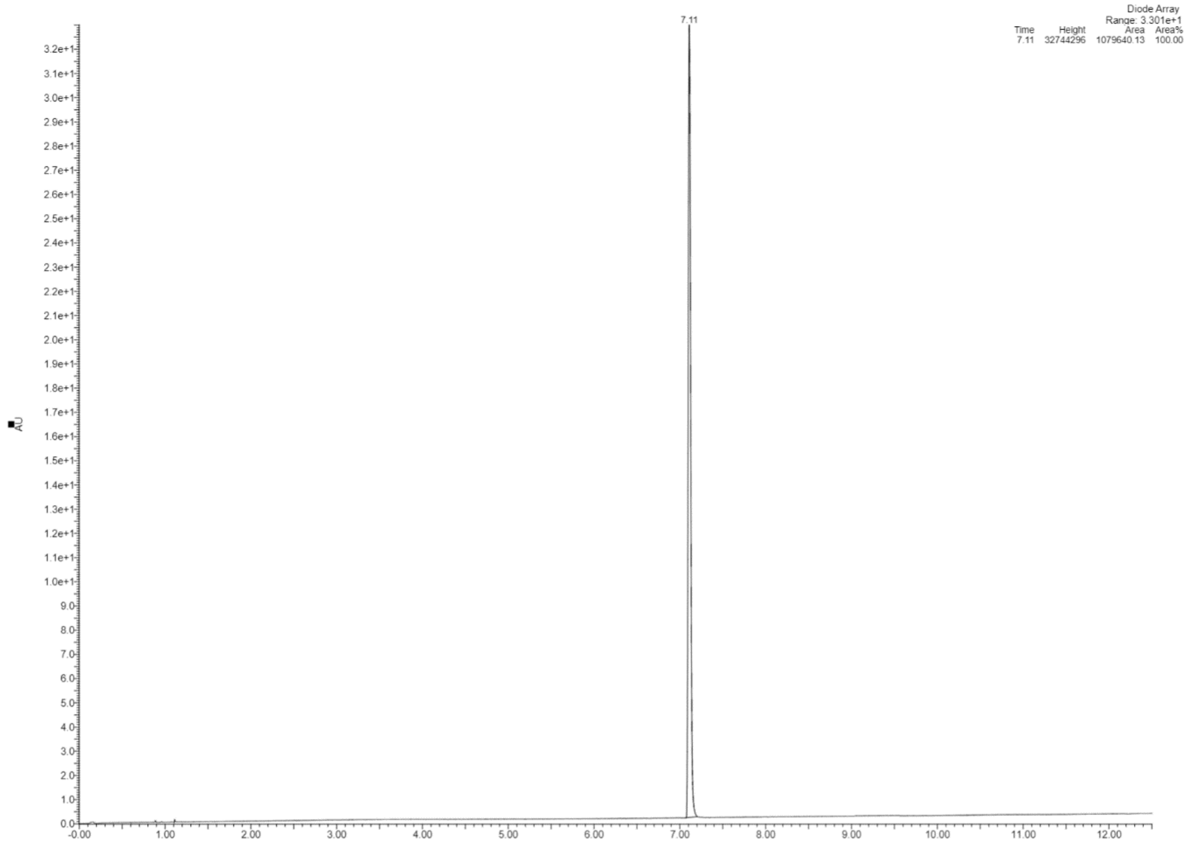

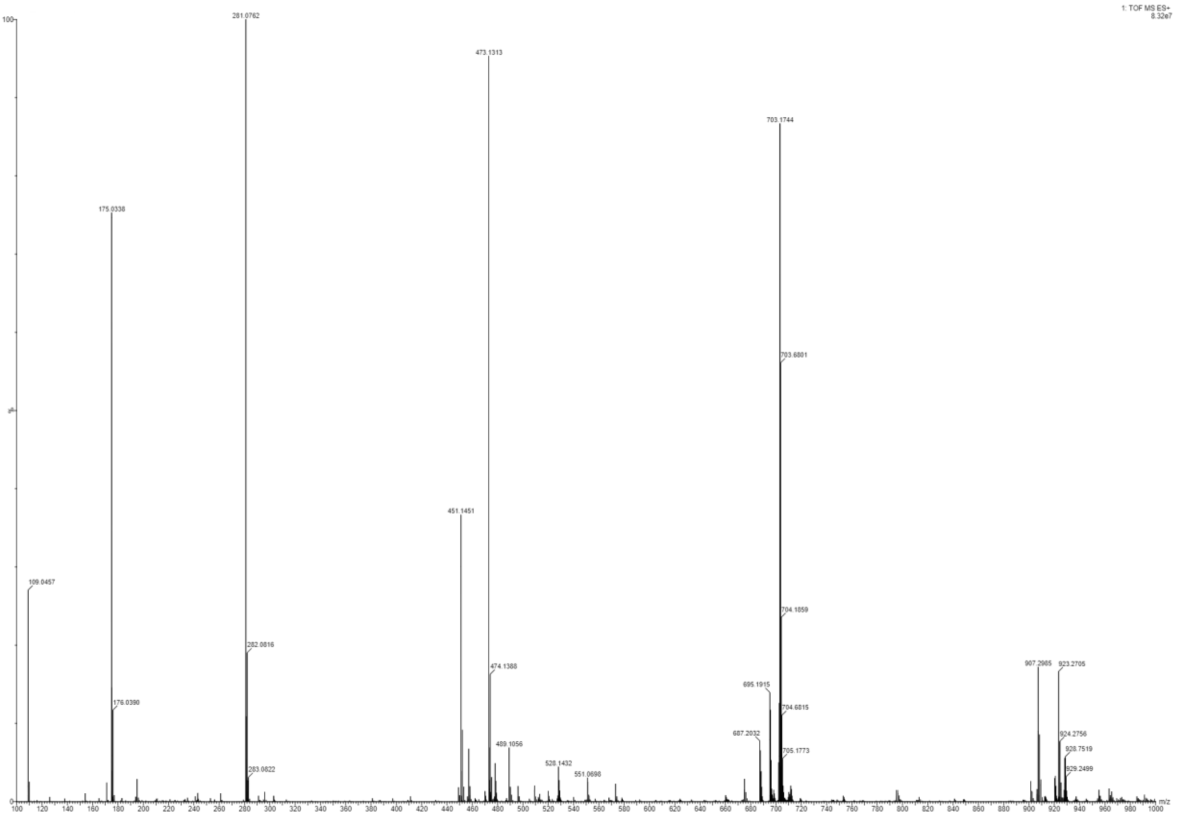

2-(2,5-dioxopyrrolidin-1-yl)-*N*-(4-((3-(trifluoromethoxy)benzyl)oxy)benzyl)propenamide (16)

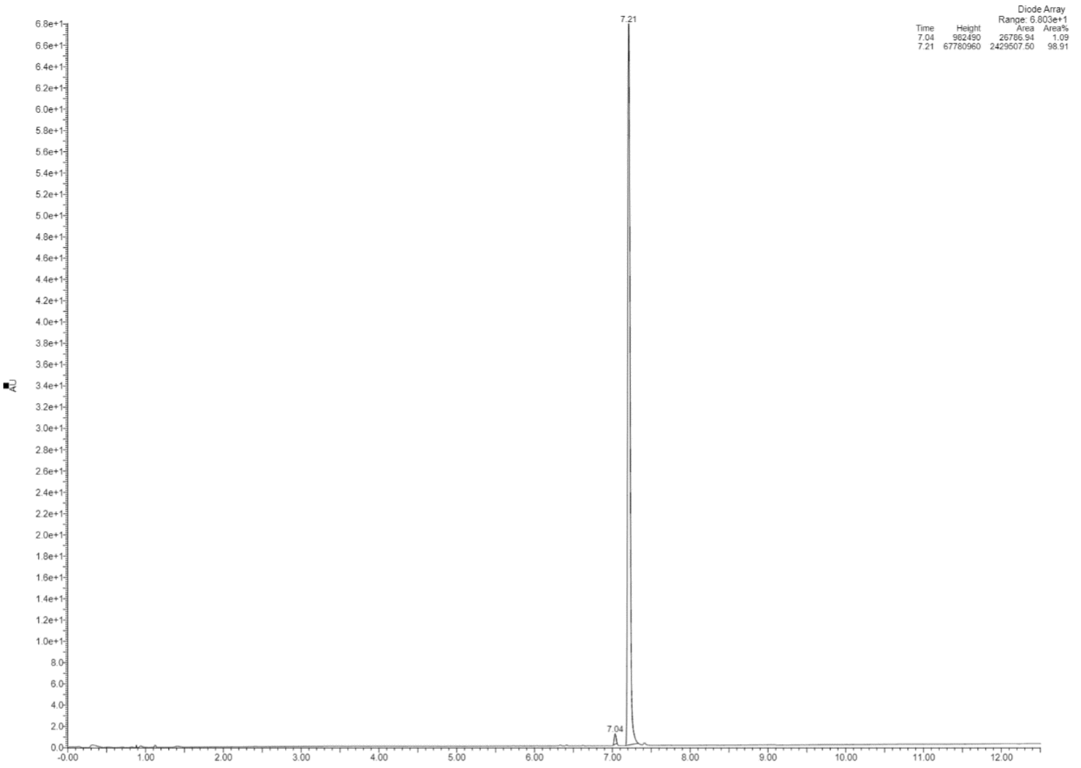

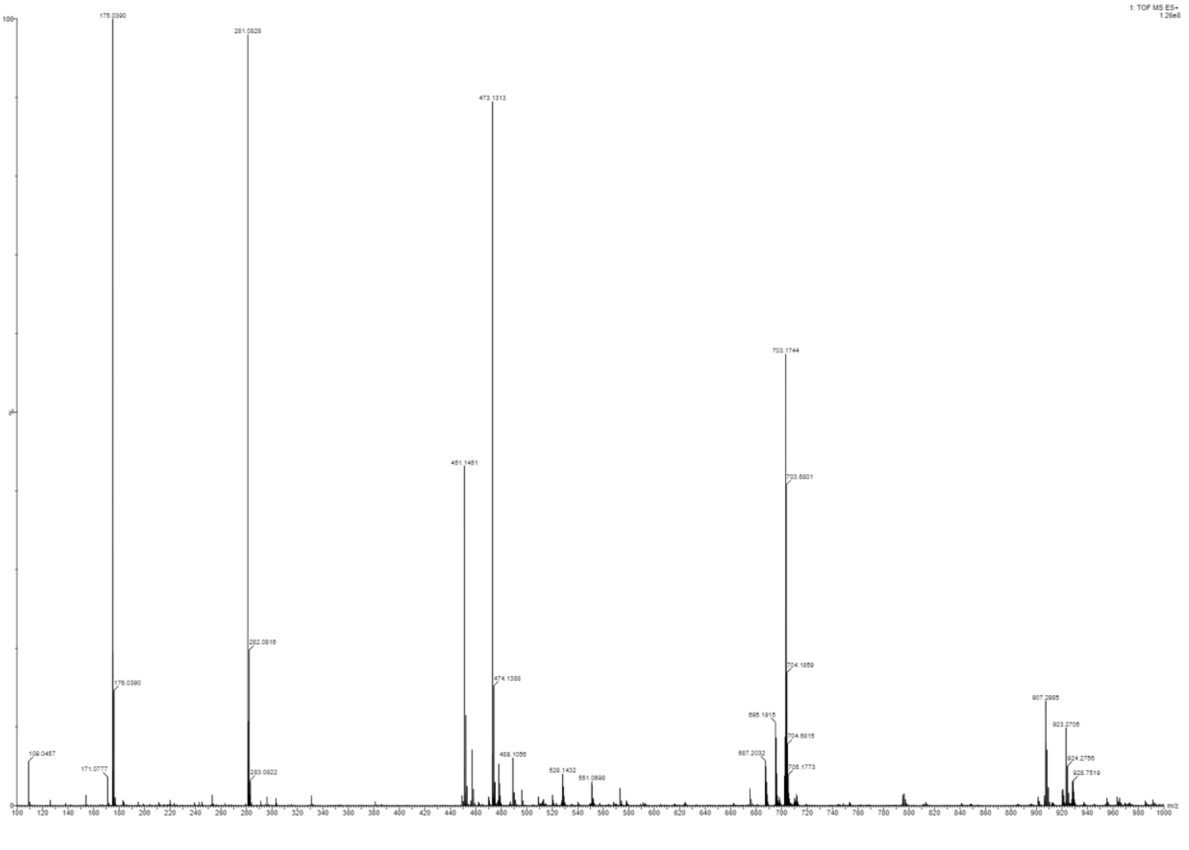

2-(2,5-dioxopyrrolidin-1-yl)-*N*-(4-((4-(trifluoromethoxy)benzyl)oxy)benzyl)propanamide (17)

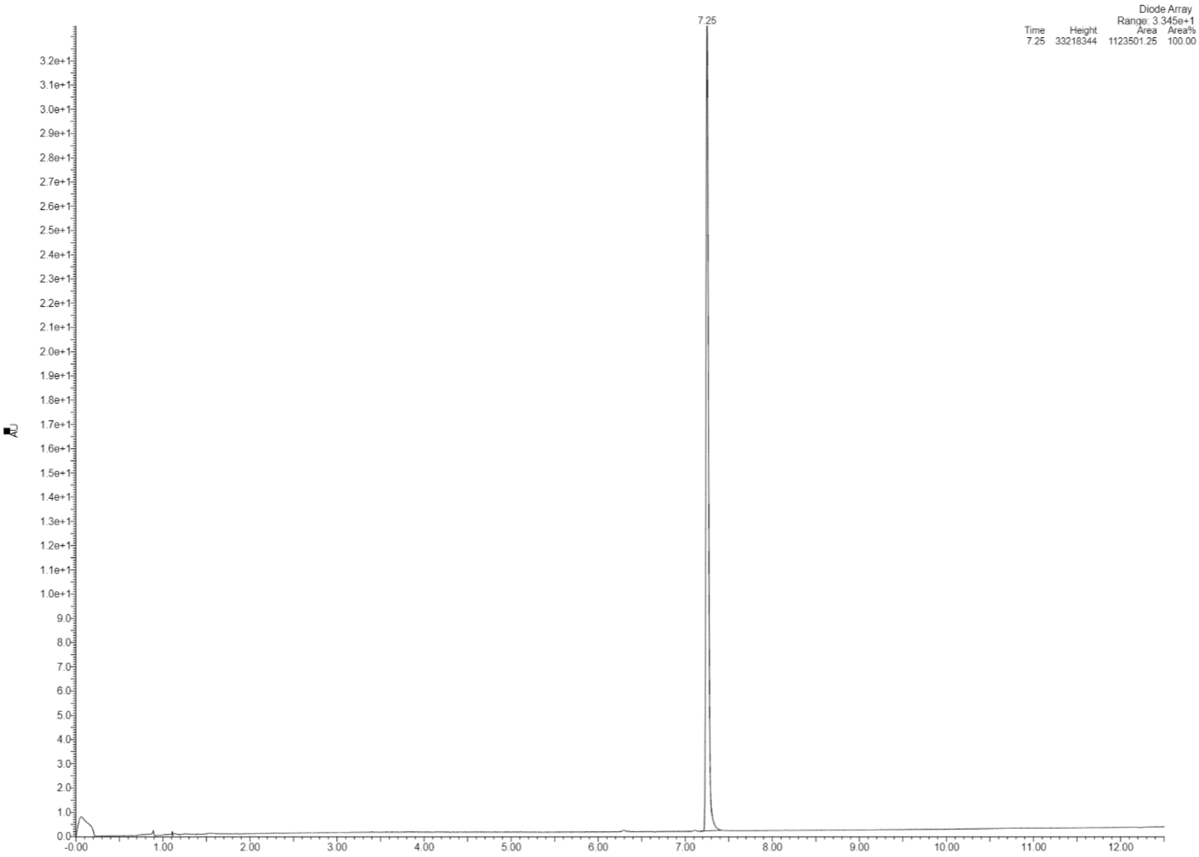

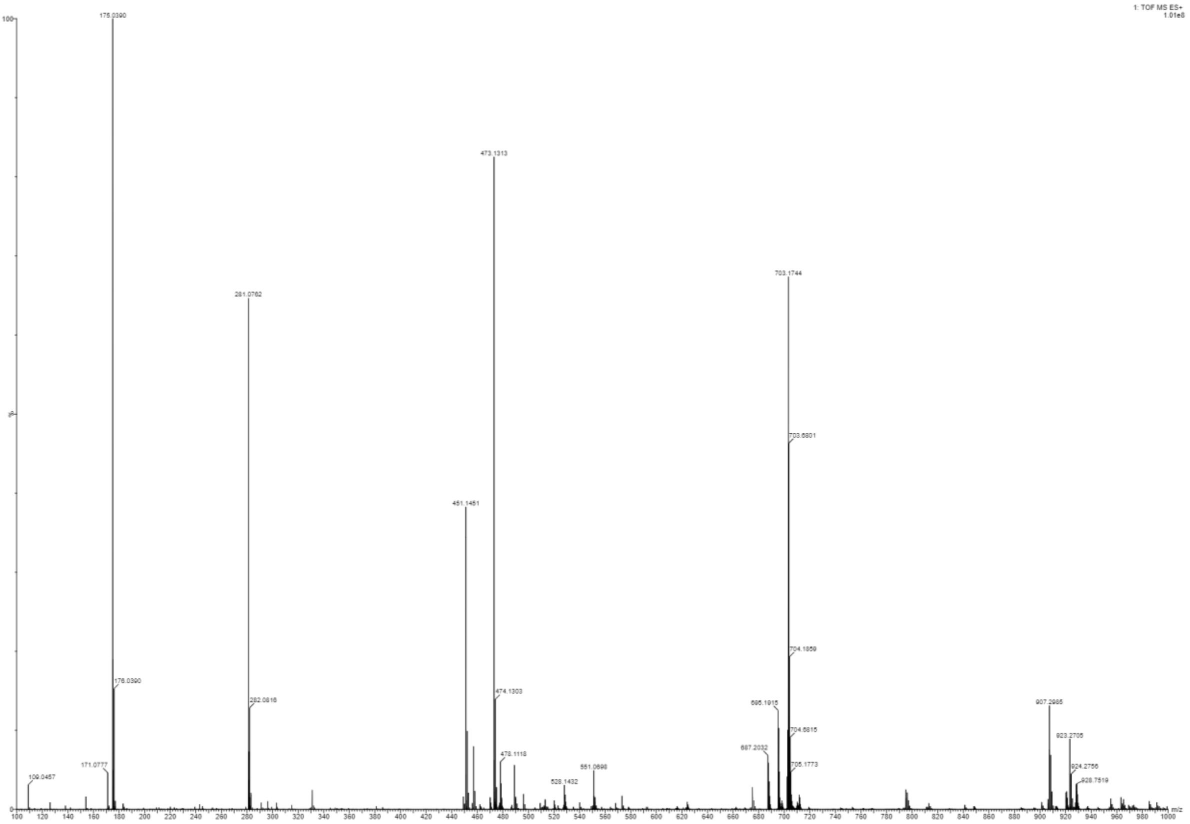

2-(2,5-dioxopyrrolidin-1-yl)-*N*-(4-((2-methylbenzyl)oxy)benzyl)propanamide (18)

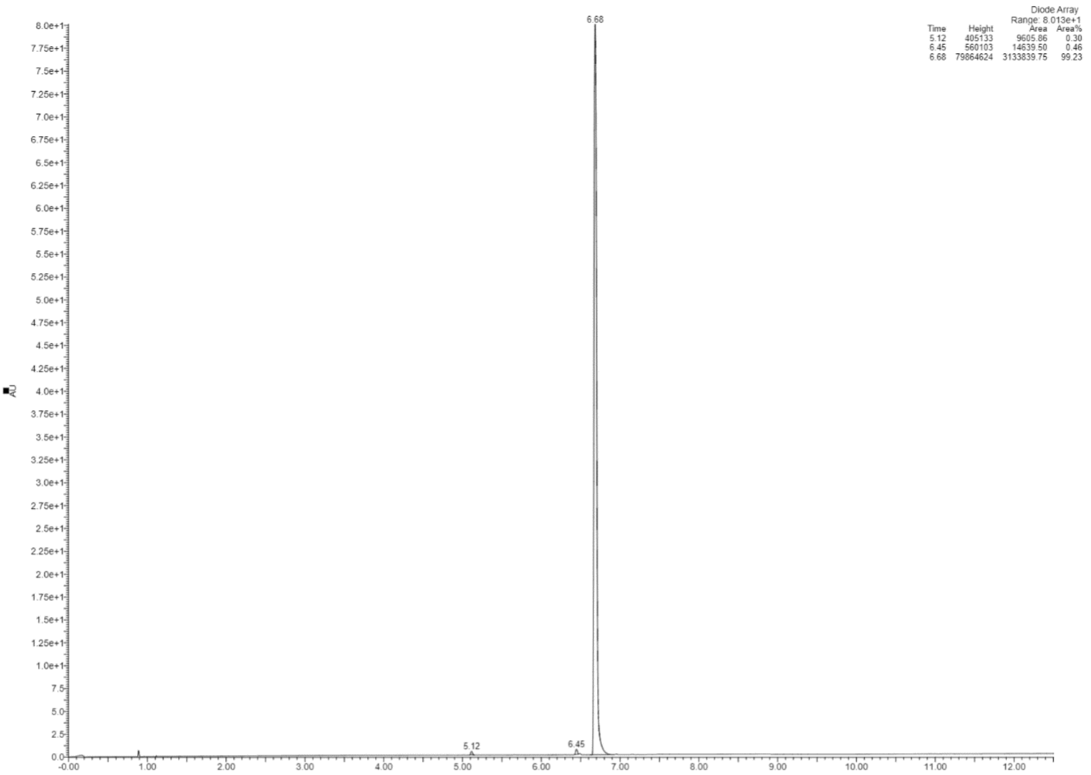

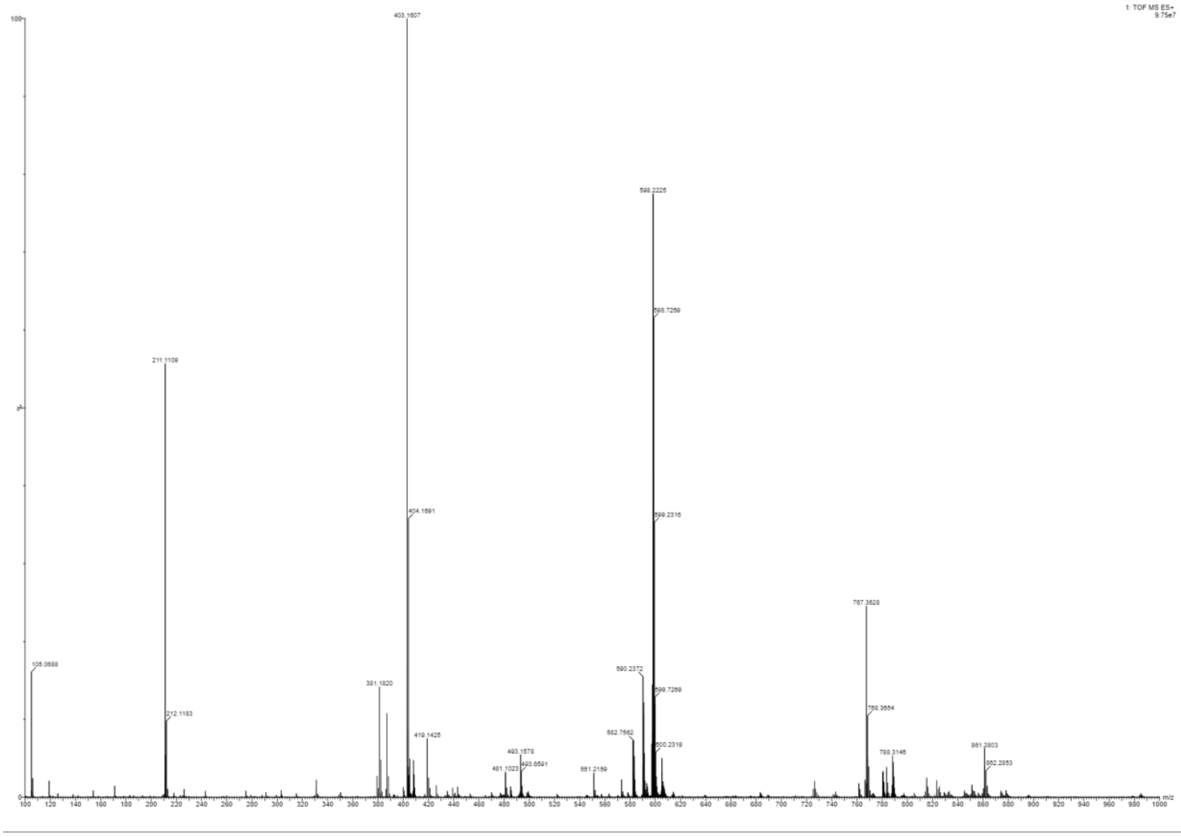

2-(2,5-dioxopyrrolidin-1-yl)-*N*-(4-((3-methylbenzyl)oxy)benzyl)propanamide (19)

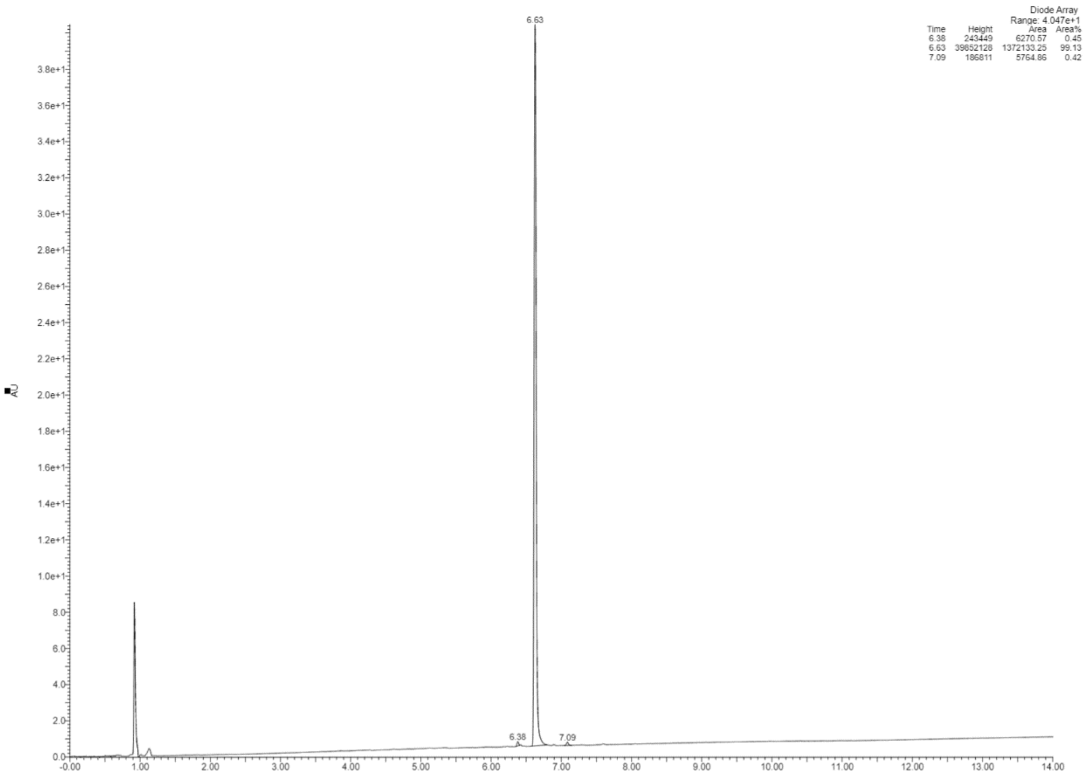

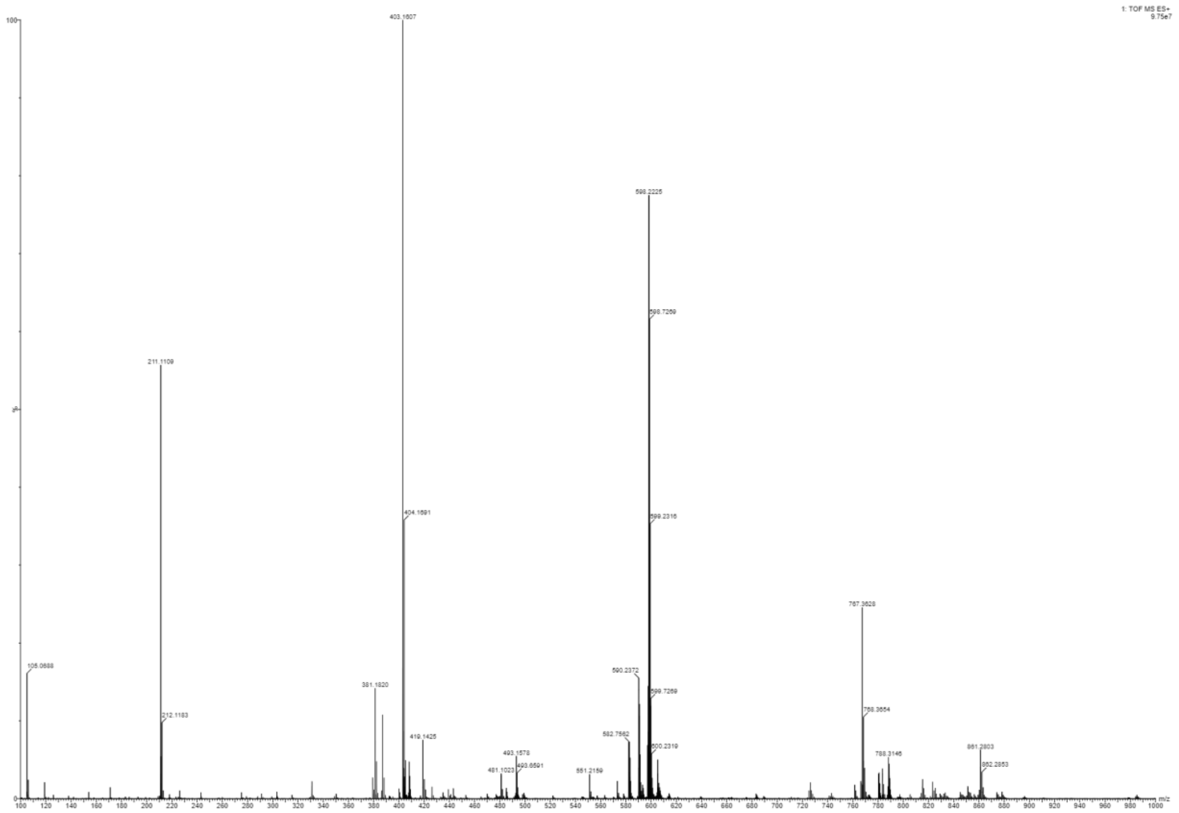

2-(2,5-dioxopyrrolidin-1-yl)-*N*-(4-((4-methylbenzyl)oxy)benzyl)propanamide (20)

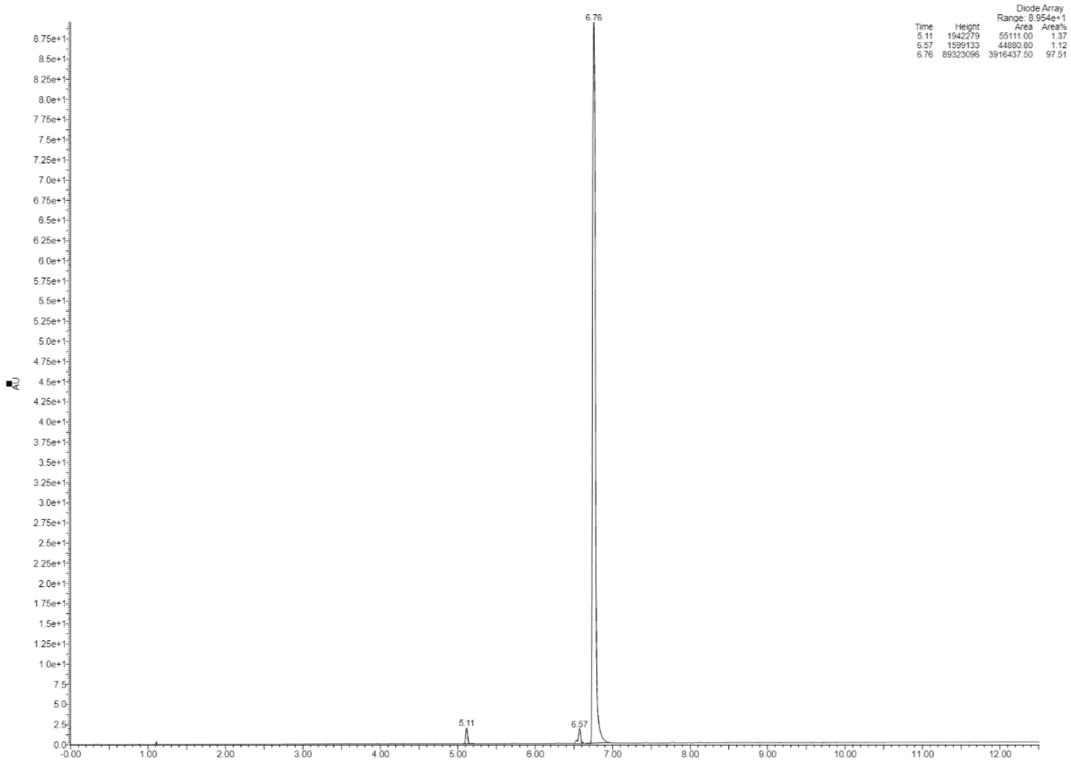

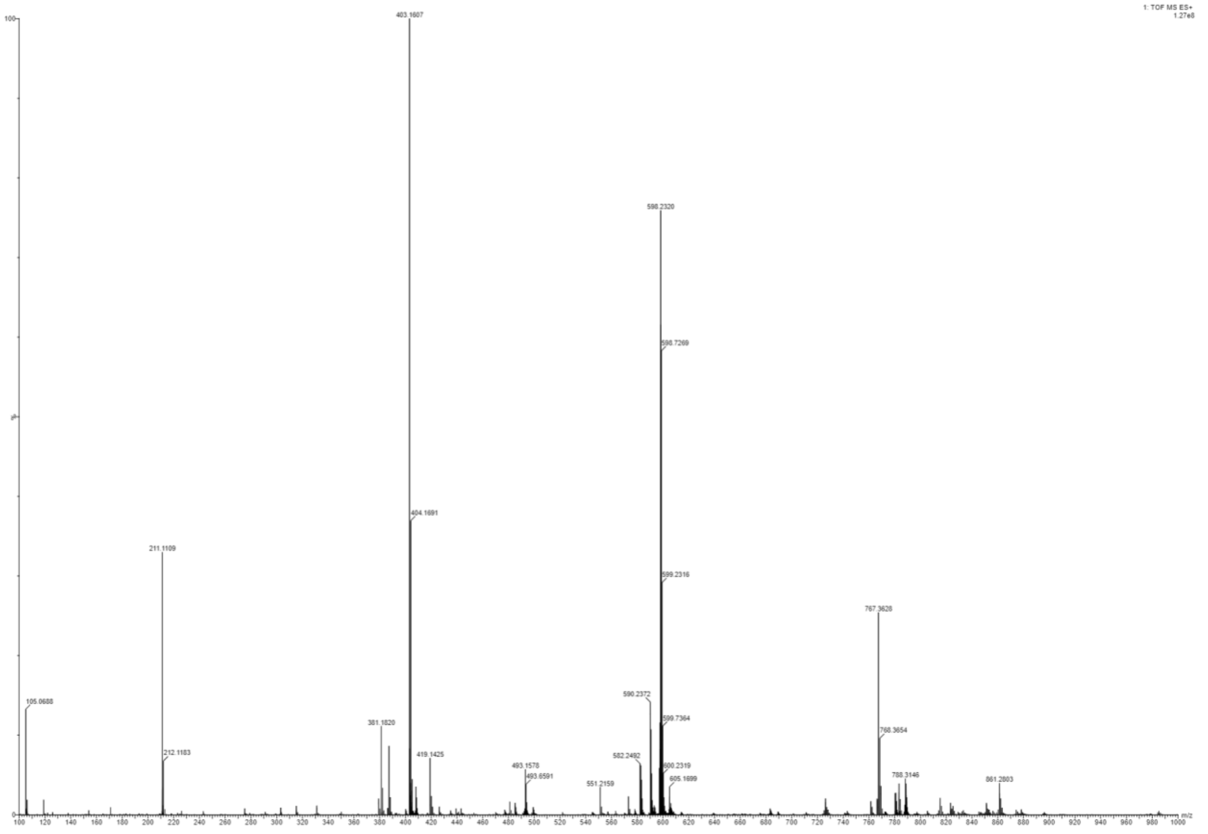

N-(4-(benzyloxy)benzyl)-2-(2,5-dioxopyrrolidin-1-yl)-2-methylpropanamide (21)

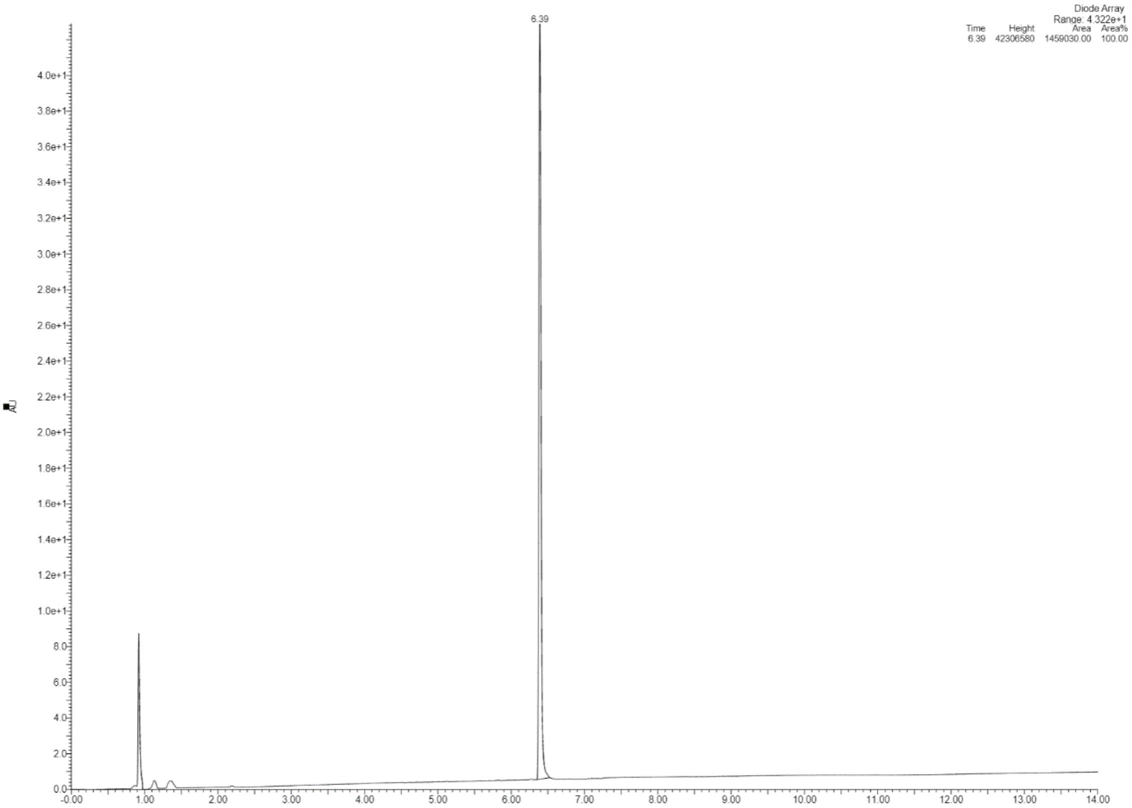

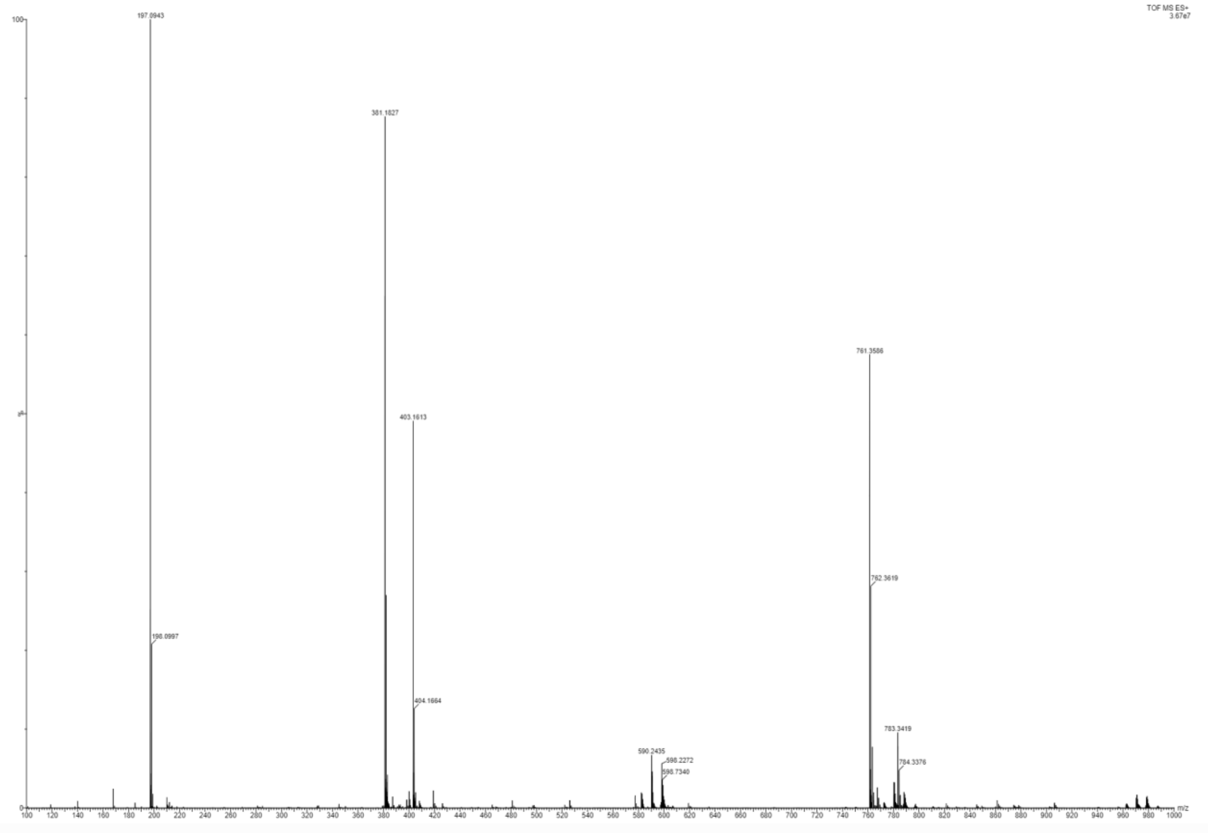

***N*-(3-(benzyloxy)benzyl)-2-(2,5-dioxopyrrolidin-1-yl)propanamide (22)**

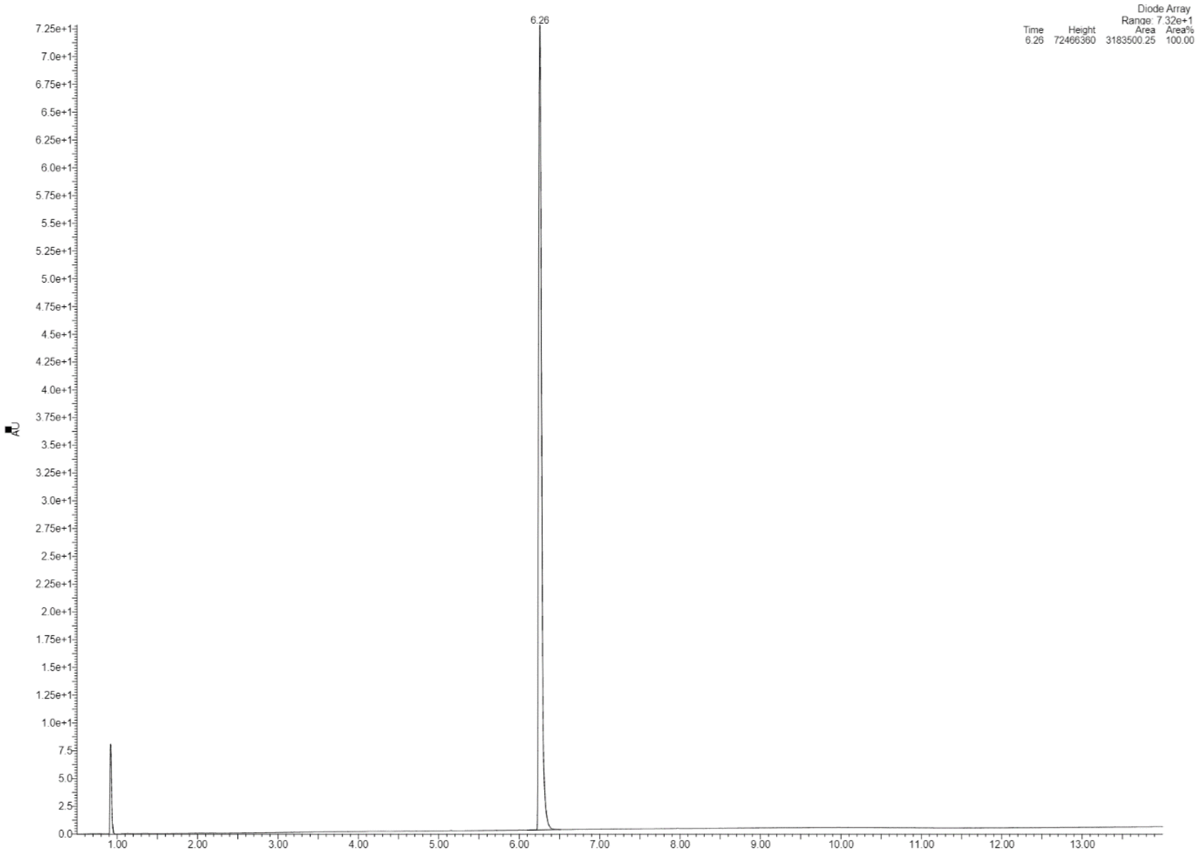

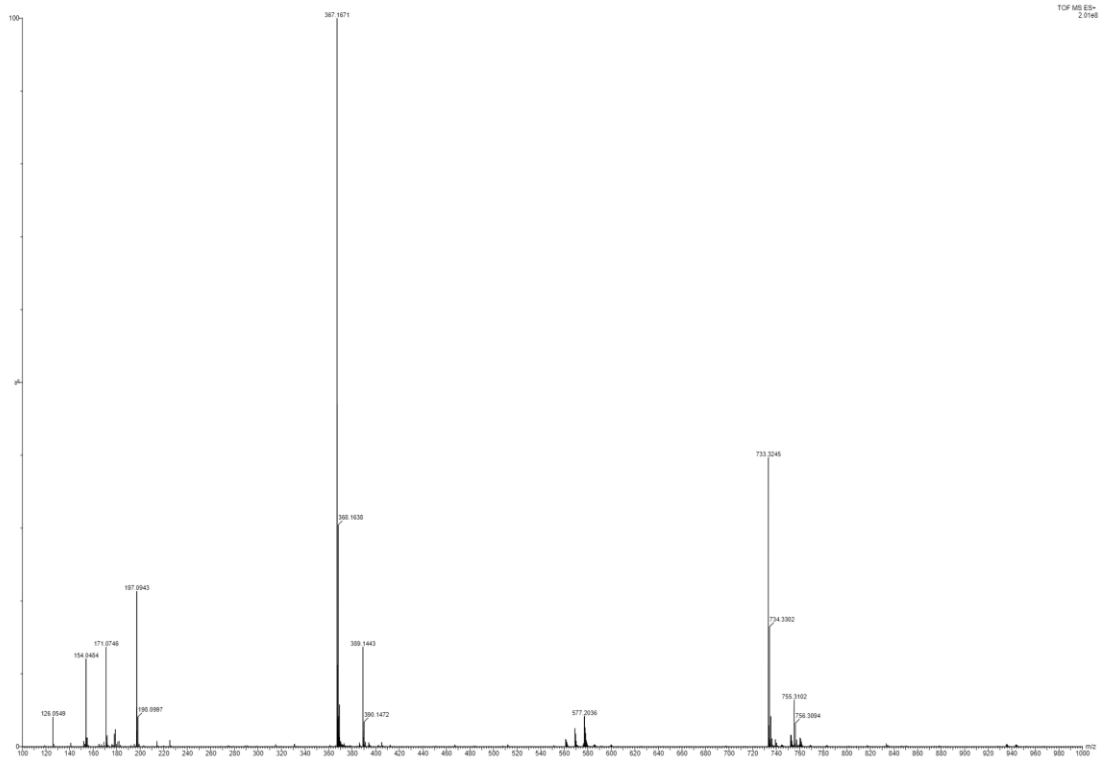

***N*-(2-(benzyloxy)benzyl)-2-(2,5-dioxopyrrolidin-1-yl)propanamide (AS-35) (23)**

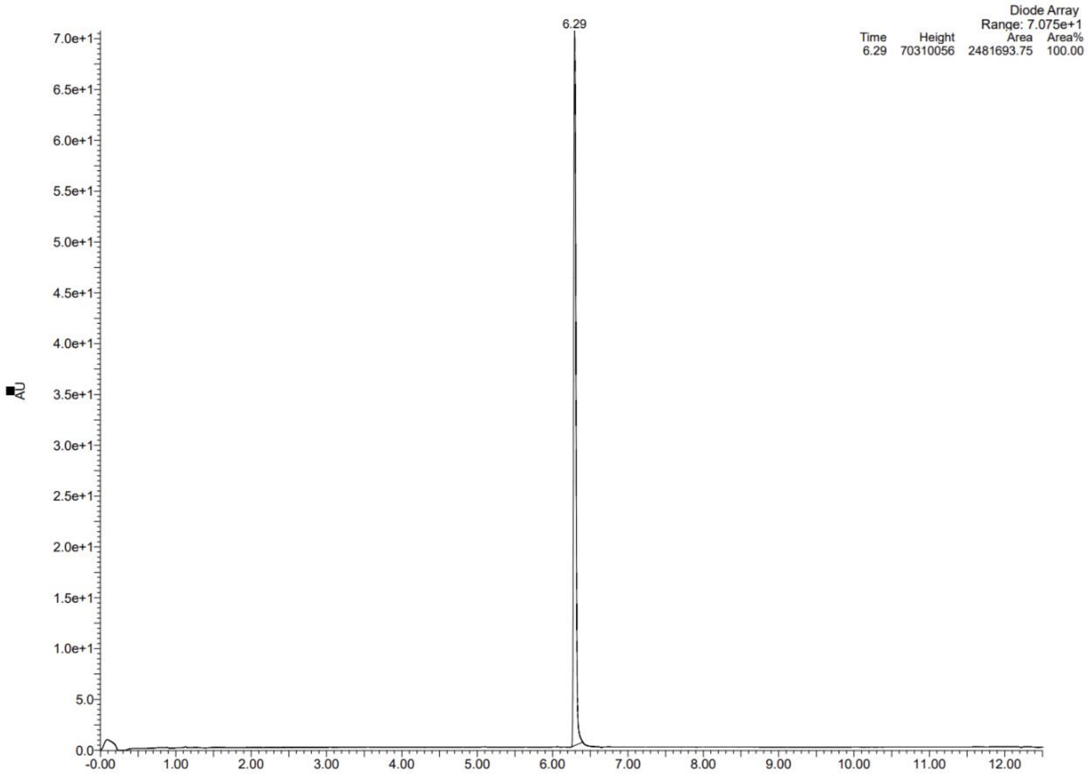

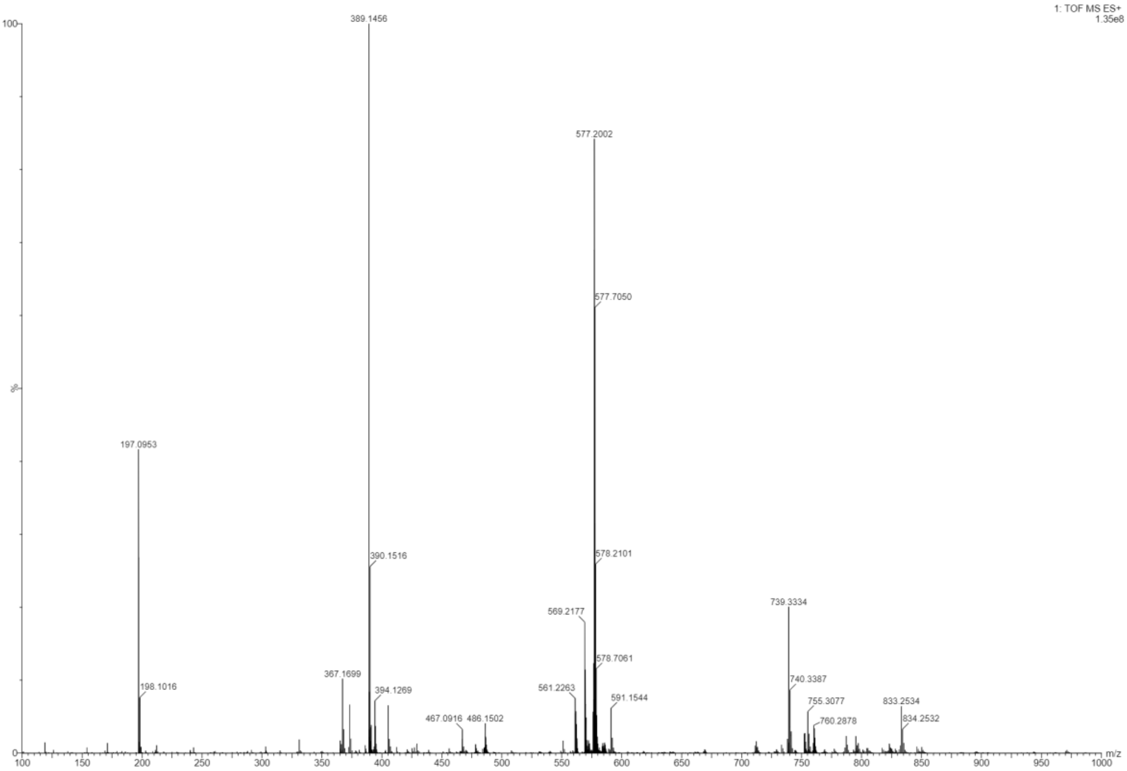

N-(4-(benzyloxy)benzyl)-2-(2-oxopyrrolidin-1-yl)propanamide (36)

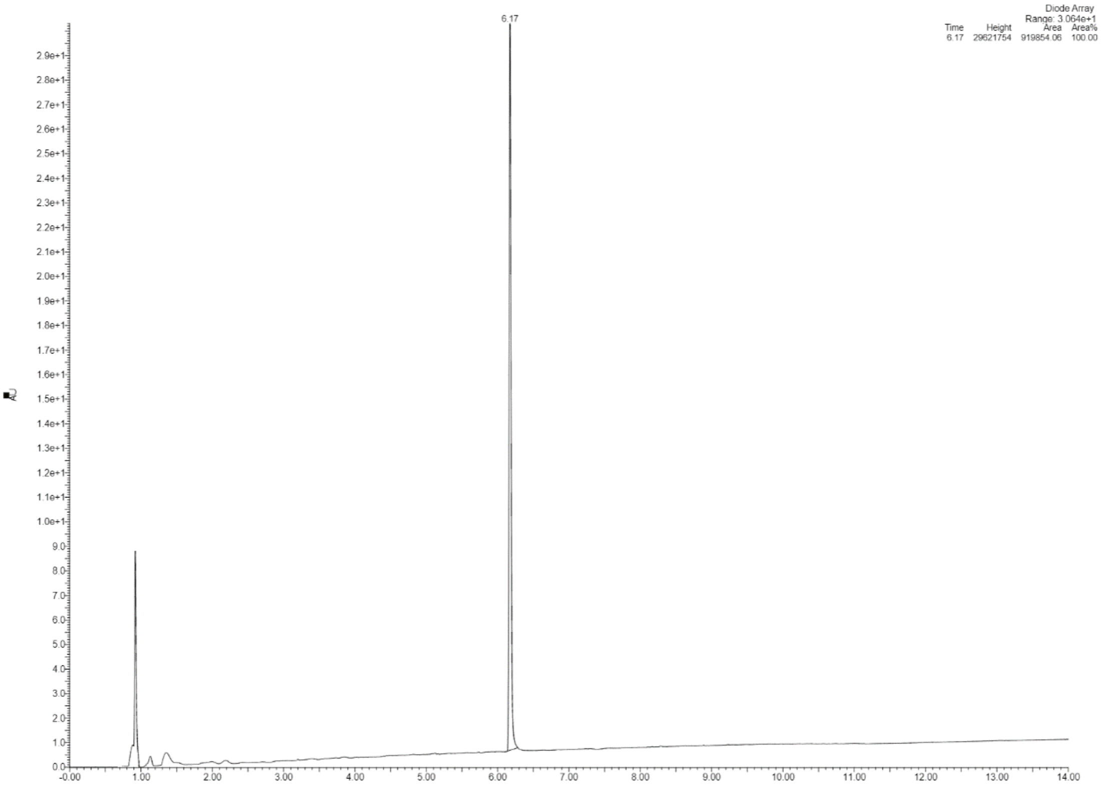

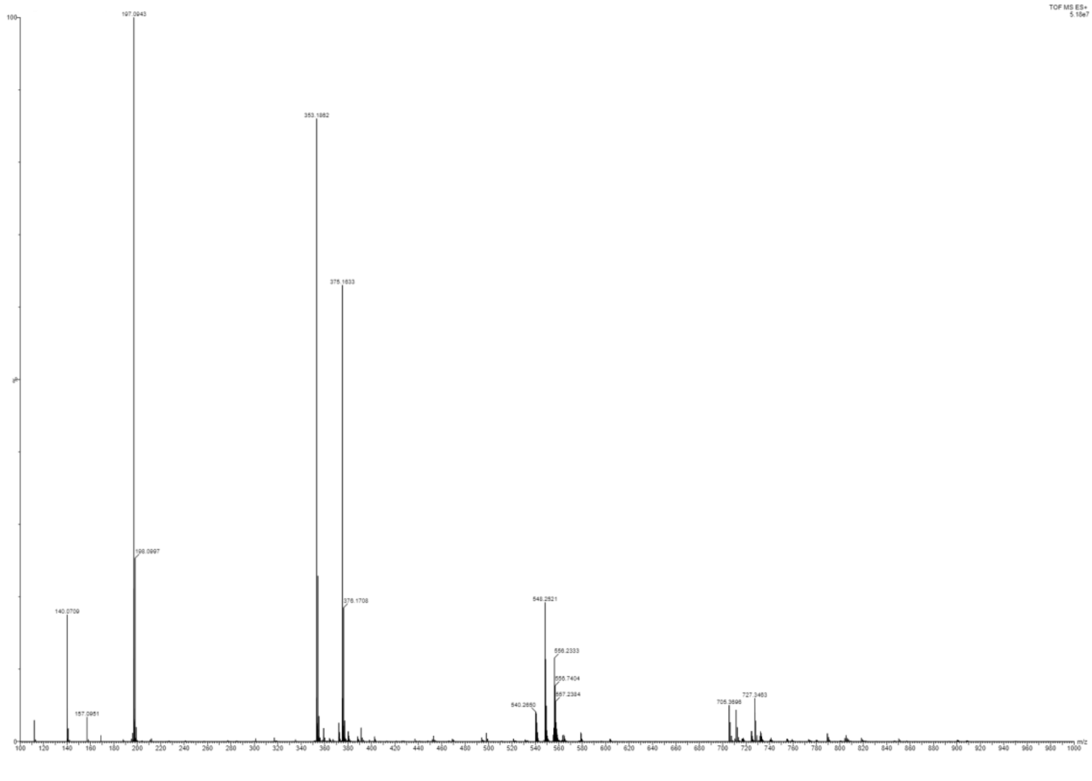

2-(2-oxopyrrolidin-1-yl)-*N*-(4-((2-(trifluoromethyl)benzyl)oxy)benzyl)propanamide (37)

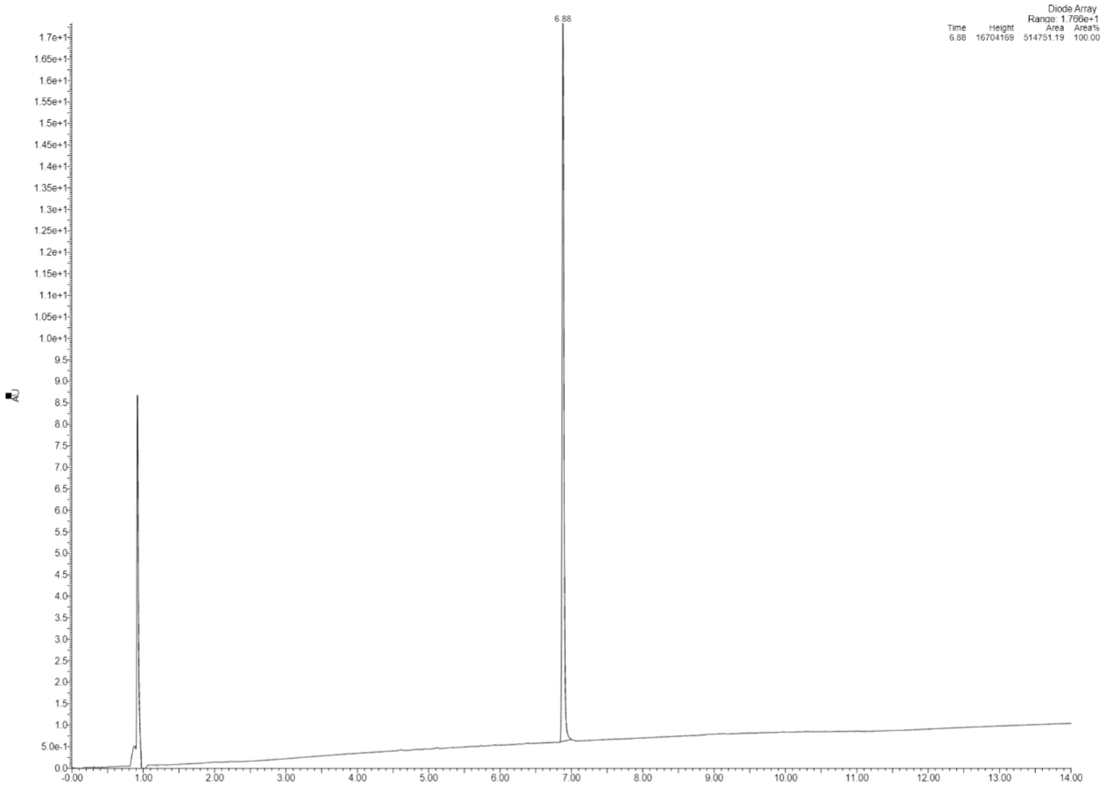

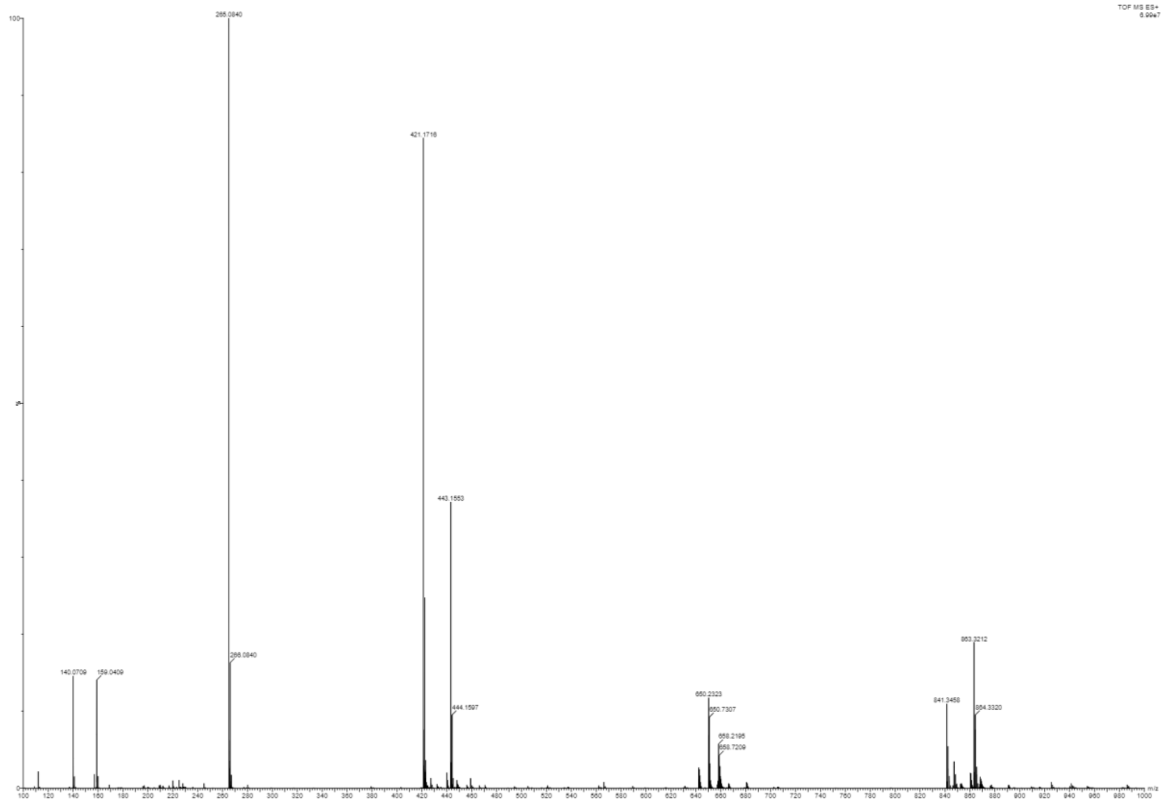

2-(2-oxopyrrolidin-1-yl)-*N*-(4-((4-(trifluoromethyl)benzyl)oxy)benzyl)propanamide (38)

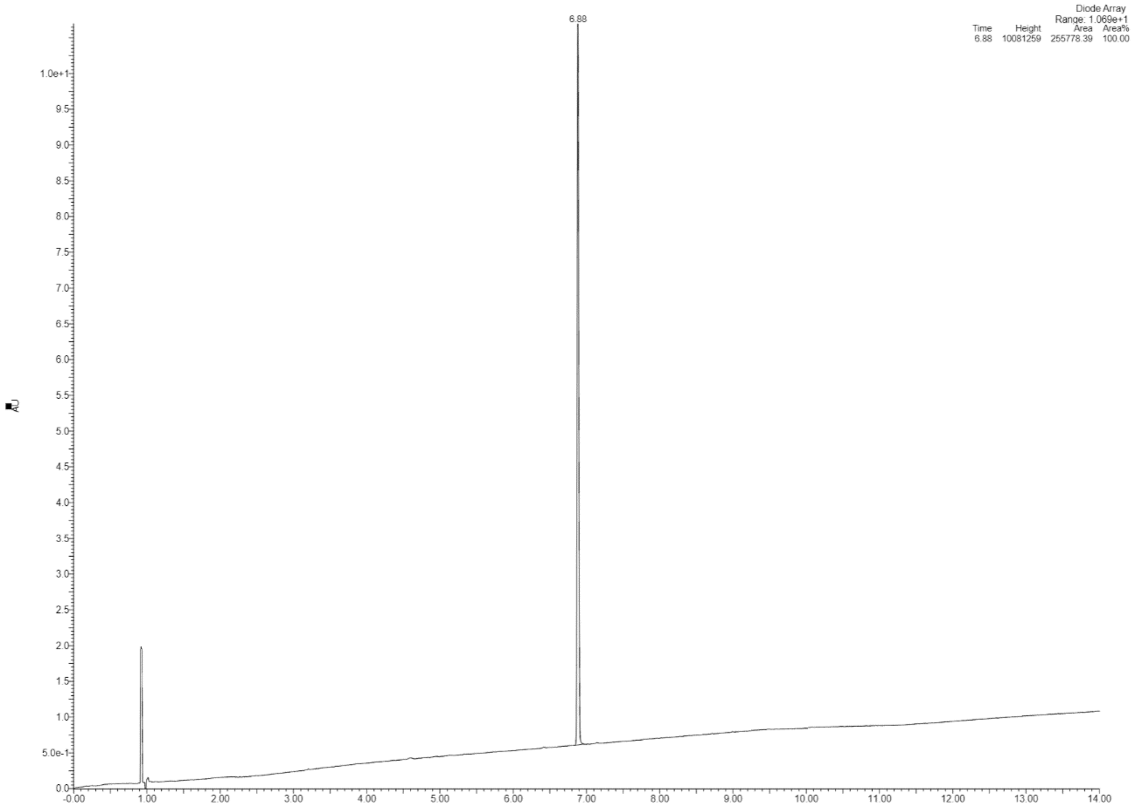

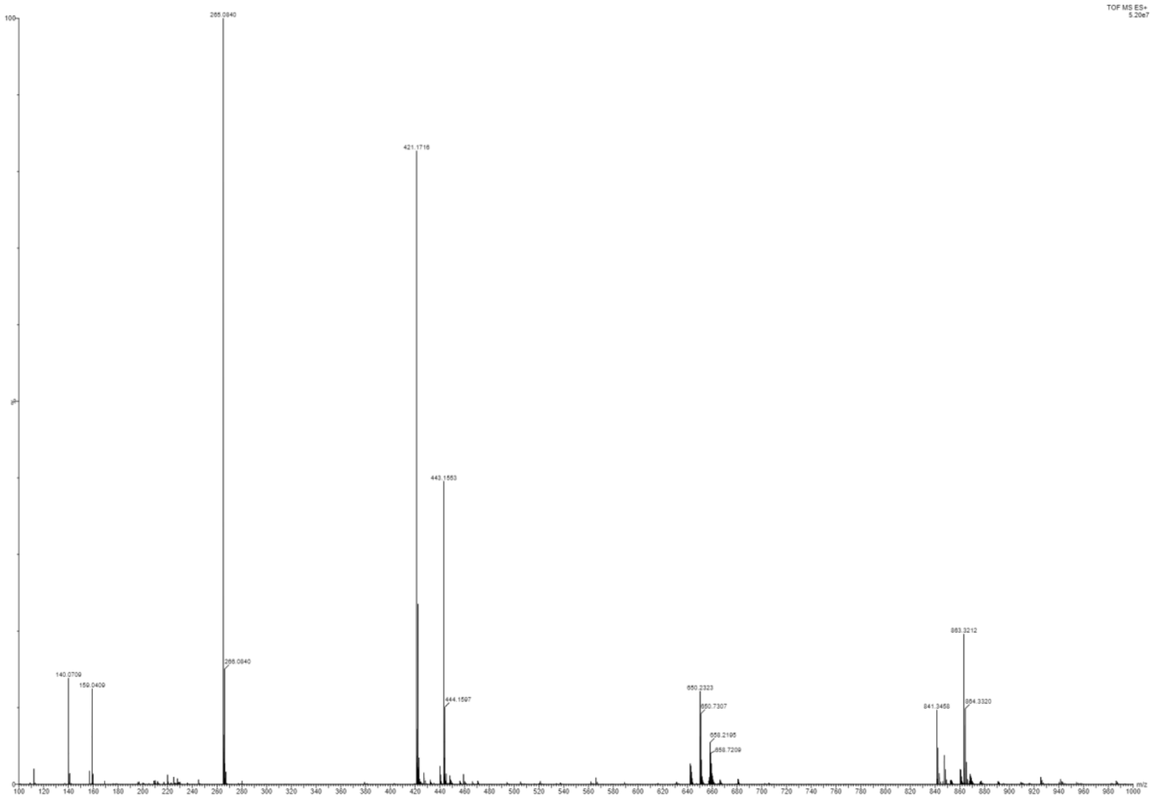

2-(2-oxopyrrolidin-1-yl)-*N*-(4-((4-(trifluoromethoxy)benzyl)oxy)benzyl)propanamide (39)

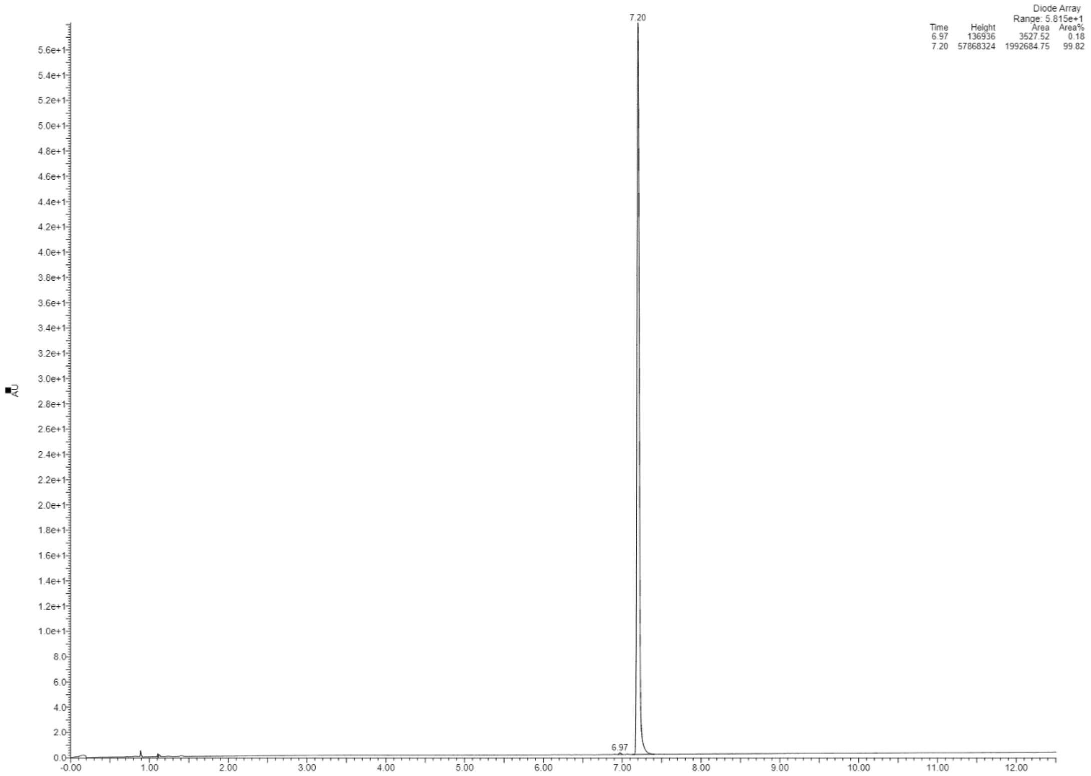

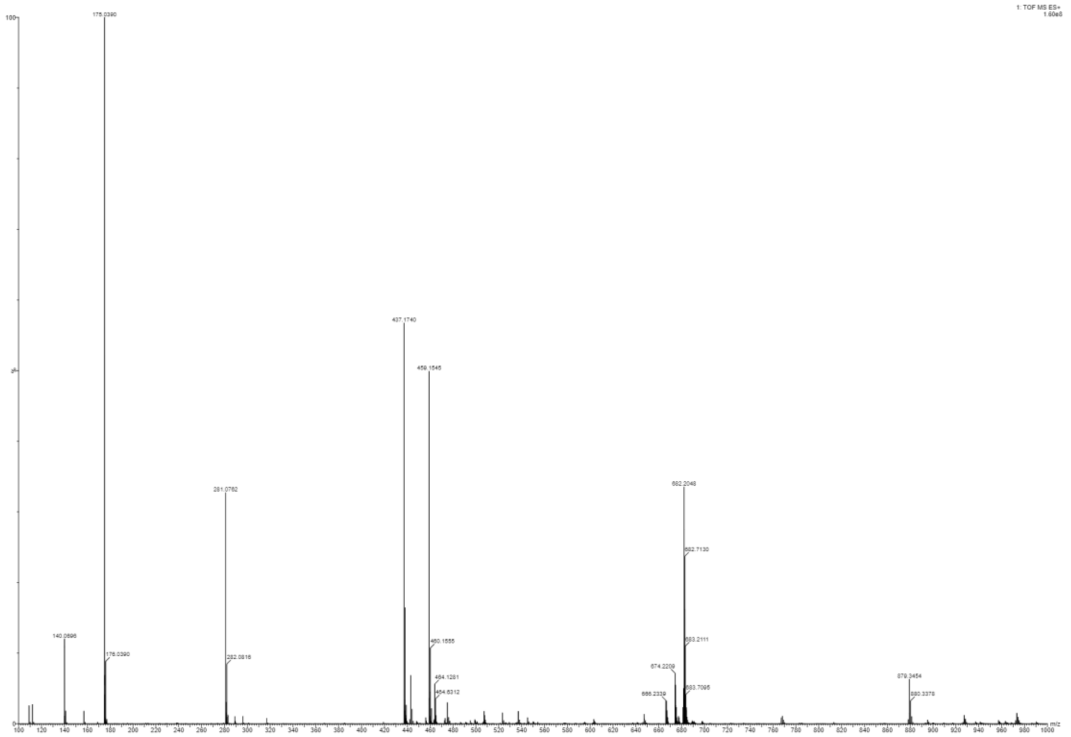

^{1}H NMR, ^{13}C NMR spectra for all final compounds

N-(4-(benzyloxy)benzyl)-2-(2,5-dioxopyrrolidin-1-yl)propanamide (5) – ^{1}H NMR

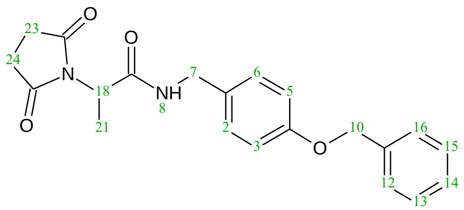

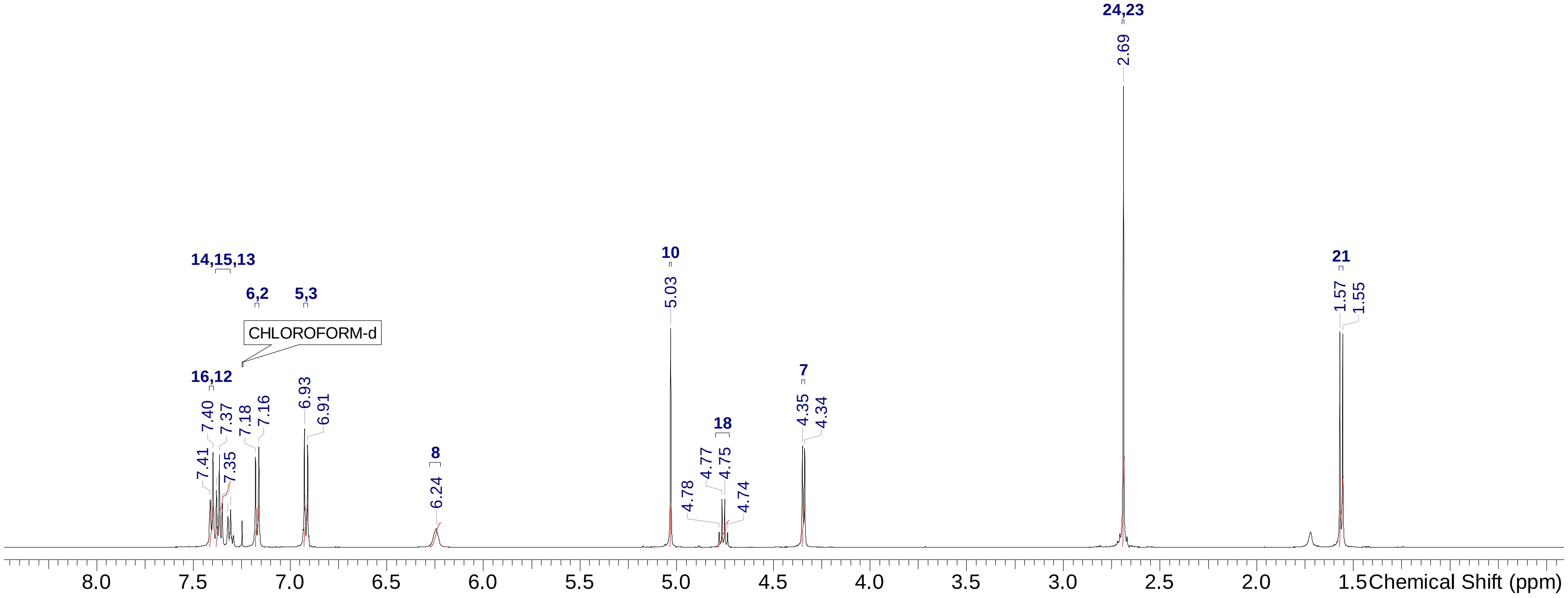

***N*-(4-(benzyloxy)benzyl)-2-(2,5-dioxopyrrolidin-1-yl)propanamide (5)** – ^{13}C NMR

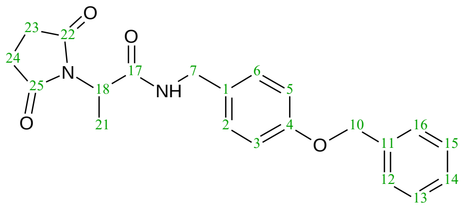

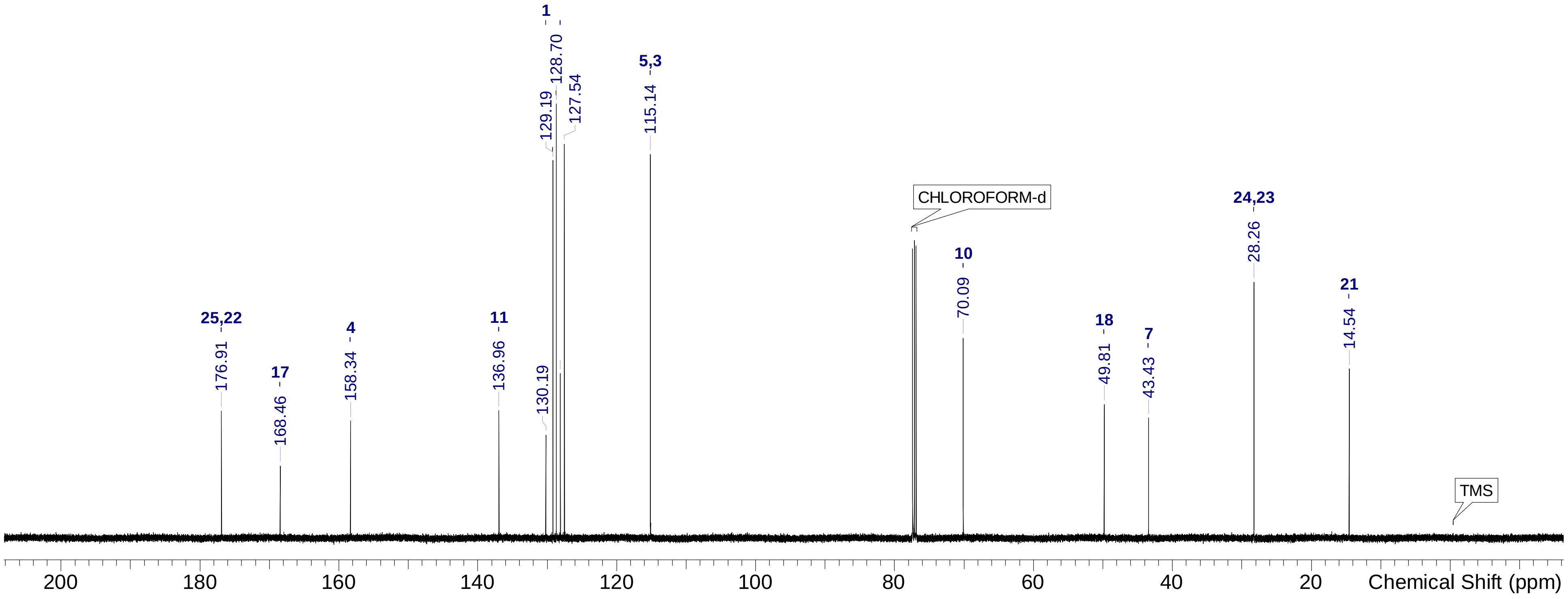

2-(2,5-dioxopyrrolidin-1-yl)-*N*-(4-((2-fluorobenzyl)oxy)benzyl)propanamide (6) – ^{1}H NMR

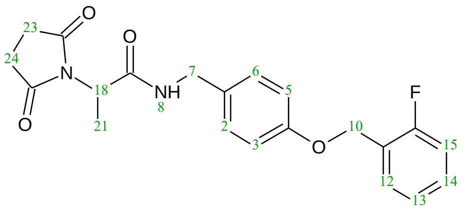

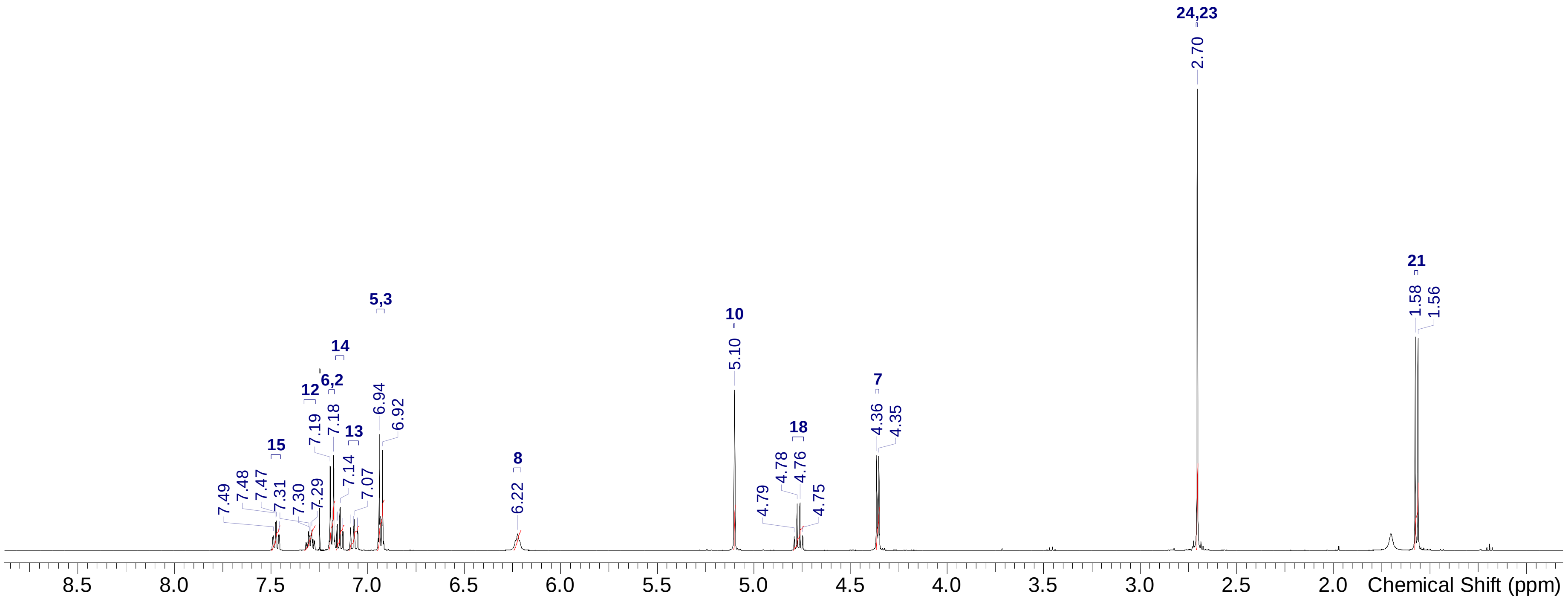

2-(2,5-dioxopyrrolidin-1-yl)-*N*-(4-((2-fluorobenzyl)oxy)benzyl)propanamide (6) – ^{13}C NMR

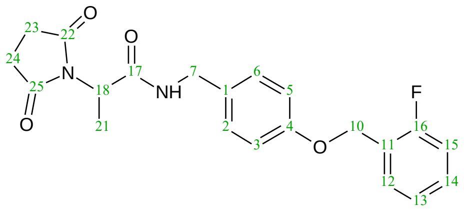

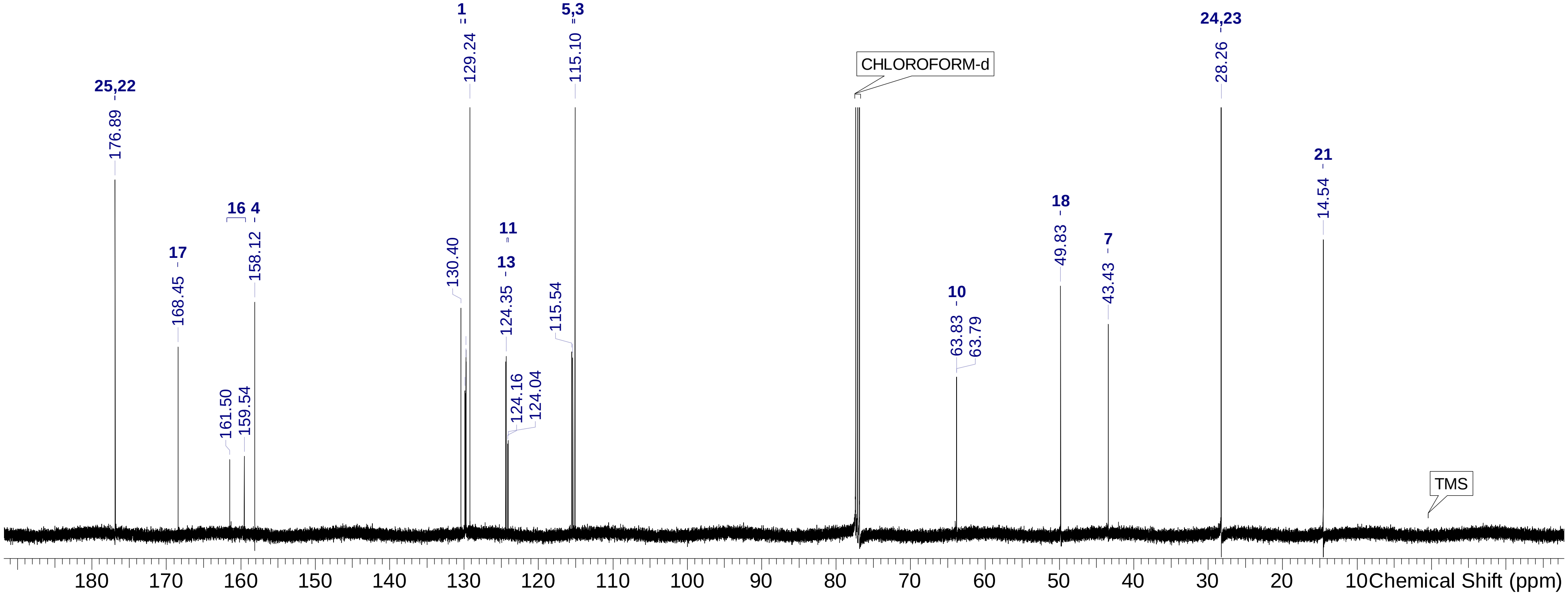

2-(2,5-dioxopyrrolidin-1-yl)-*N*-(4-((3-fluorobenzyl)oxy)benzyl)propanamide (7) – ^{1}H NMR

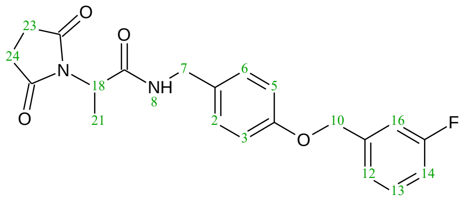

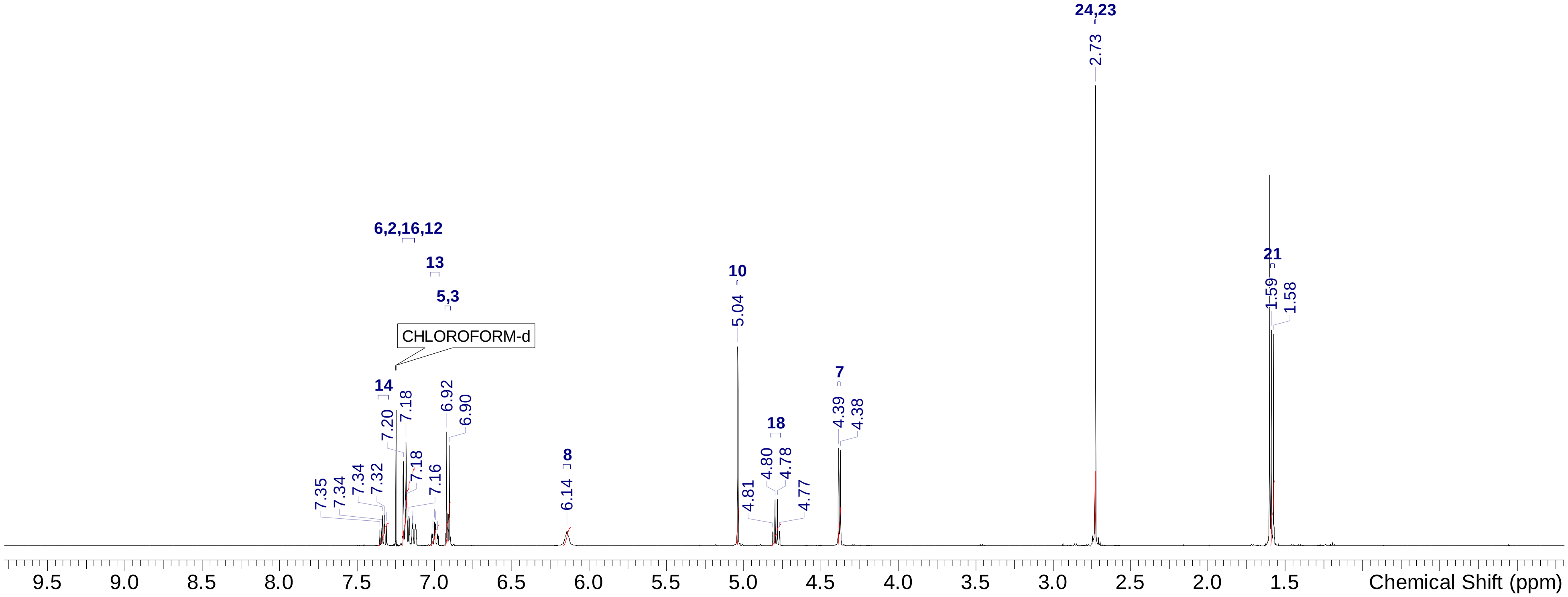

2-(2,5-dioxopyrrolidin-1-yl)-*N*-(4-((3-fluorobenzyl)oxy)benzyl)propanamide (7) – ^{13}C NMR

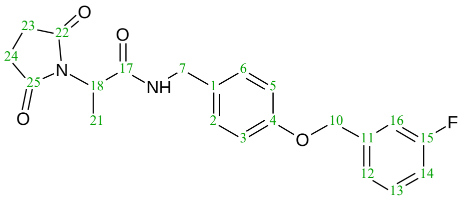

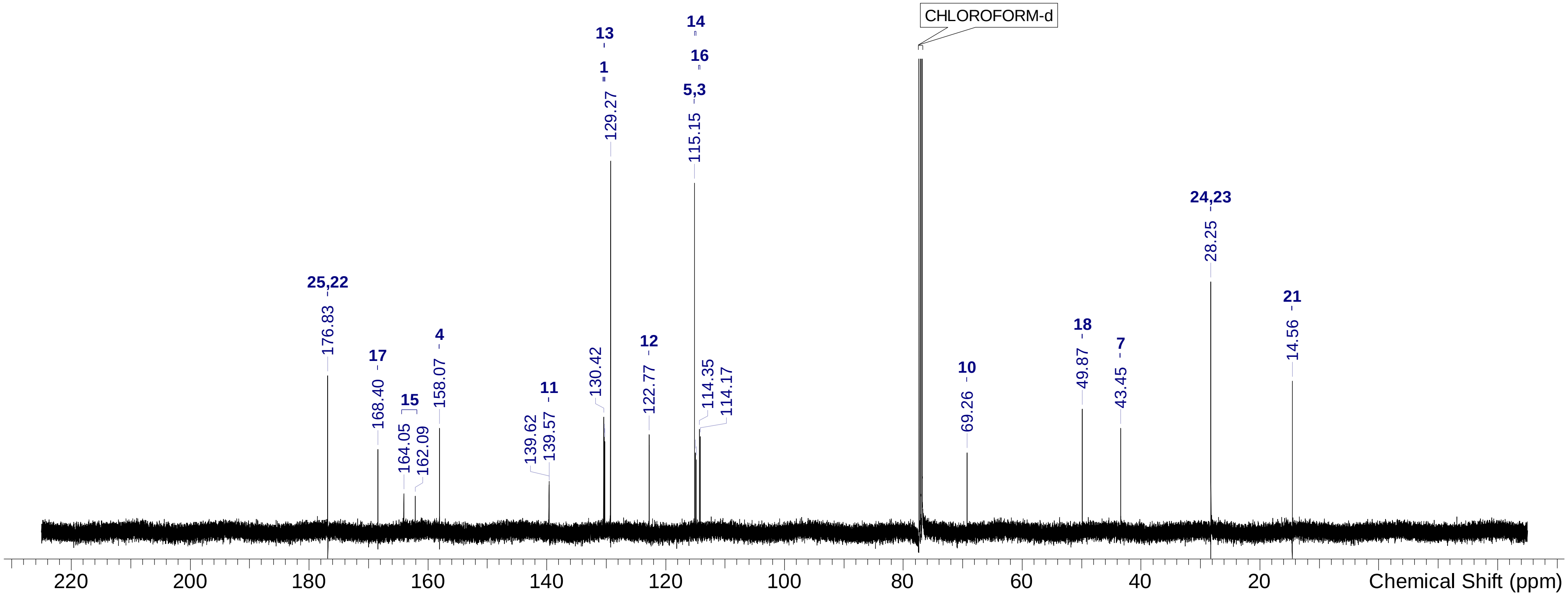

2-(2,5-dioxopyrrolidin-1-yl)-*N*-(4-((4-fluorobenzyl)oxy)benzyl)propanamide (8) – ^{1}H NMR

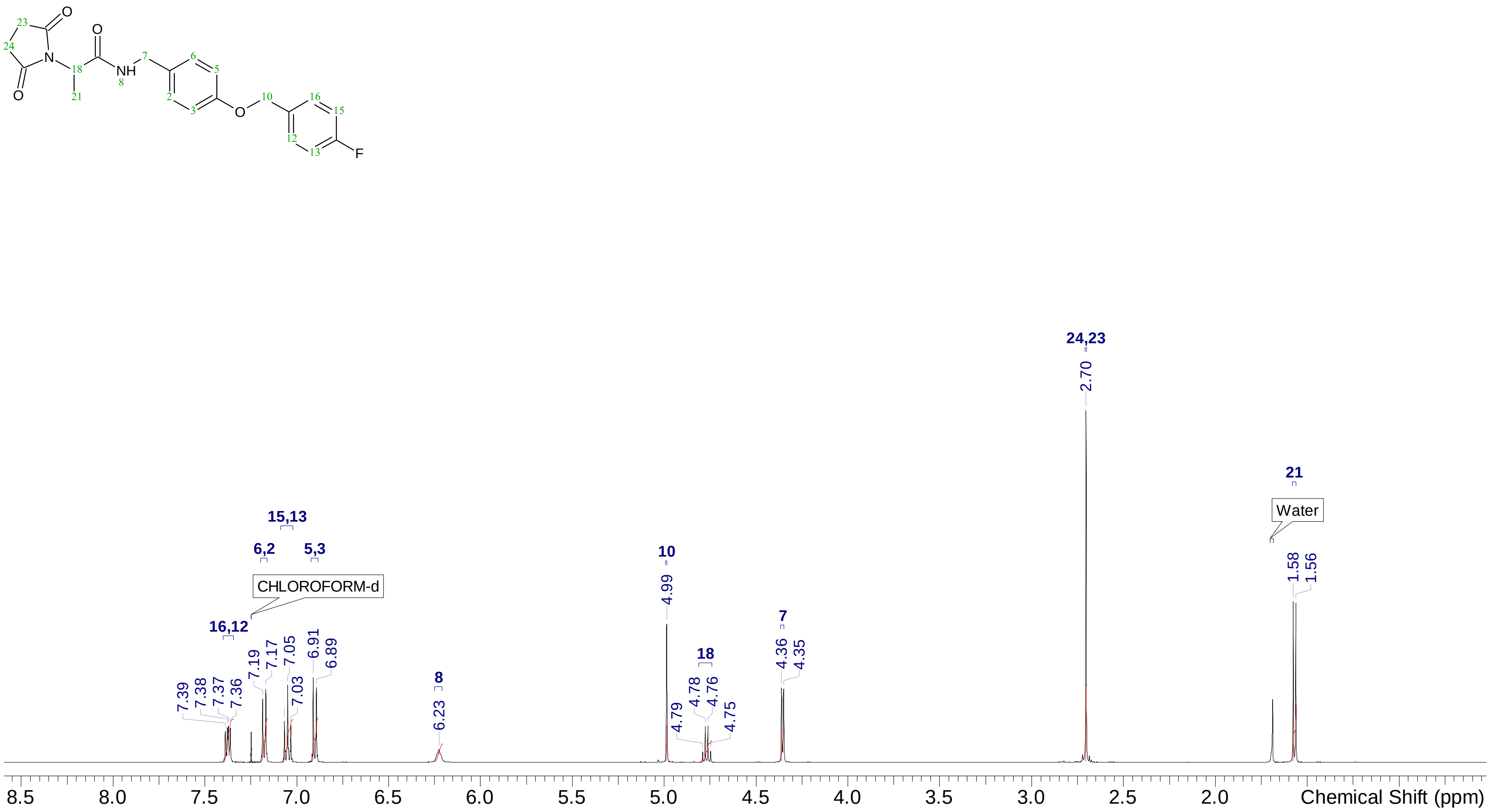

2-(2,5-dioxopyrrolidin-1-yl)-*N*-(4-((4-fluorobenzyl)oxy)benzyl)propanamide (8) – ^{13}C NMR

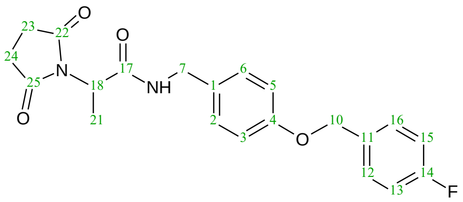

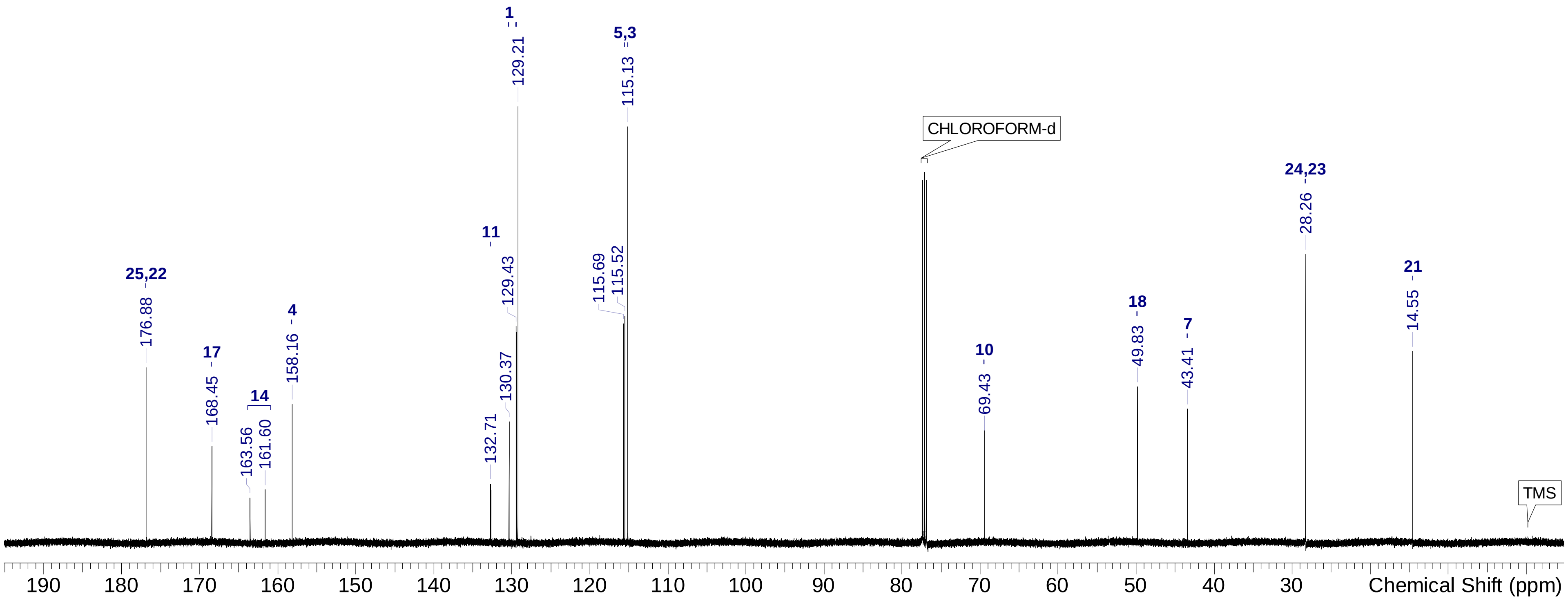

***N*-(4-((2-chlorobenzyl)oxy)benzyl)-2-(2,5-dioxopyrrolidin-1-yl)propanamide (9)** – ^{1}H NMR

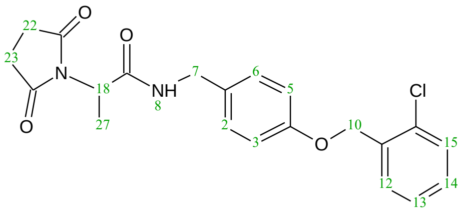

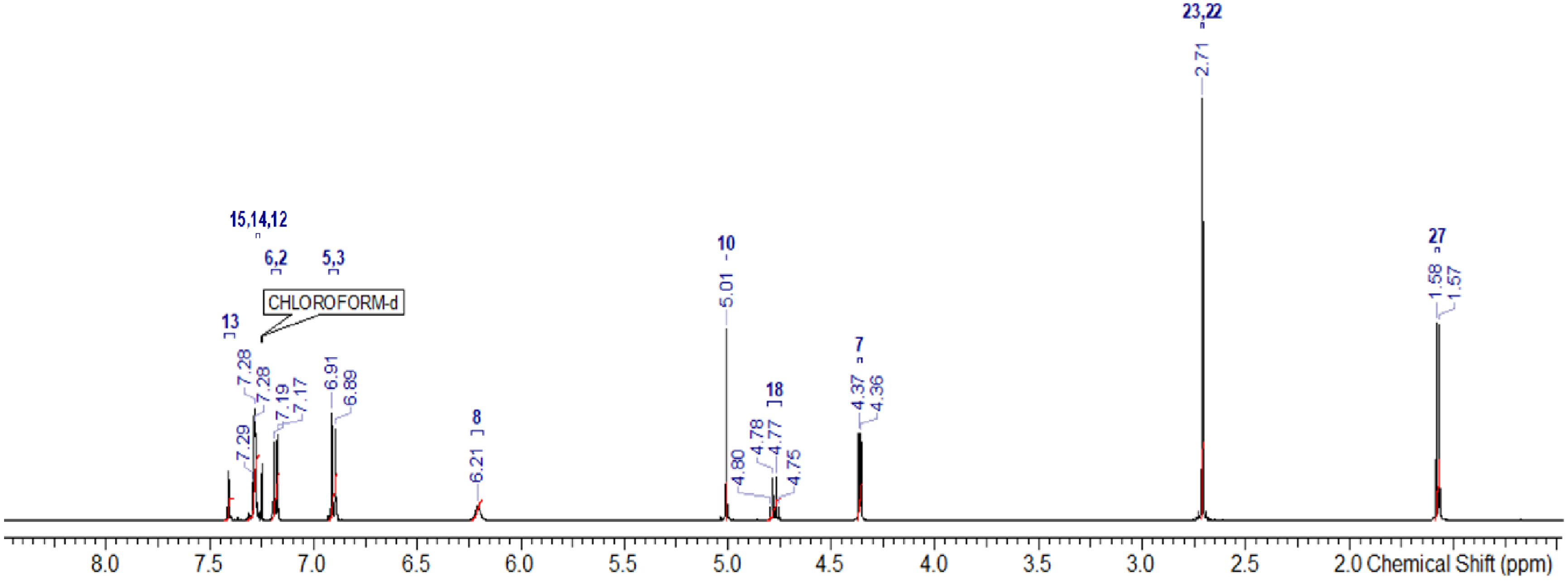

***N*-(4-((2-chlorobenzyl)oxy)benzyl)-2-(2,5-dioxopyrrolidin-1-yl)propanamide (9)** – ^{13}C NMR

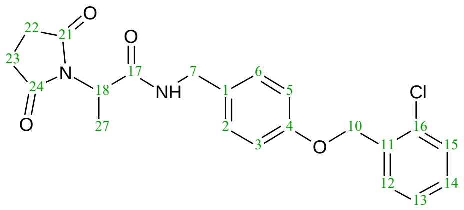

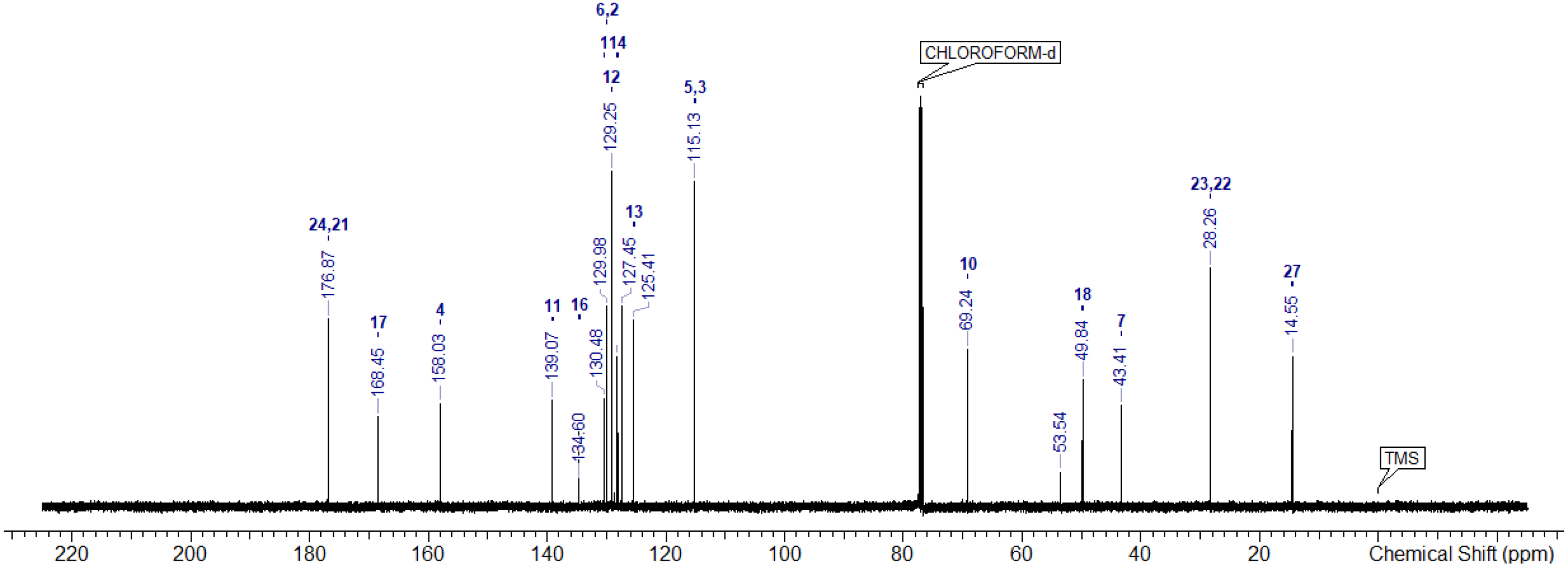

***N*-(4-((3-chlorobenzyl)oxy)benzyl)-2-(2,5-dioxopyrrolidin-1-yl)propanamide (10)** – ^{1}H NMR

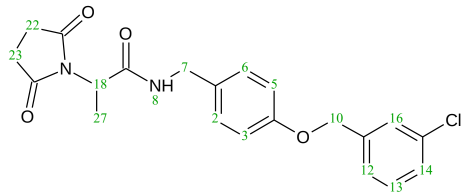

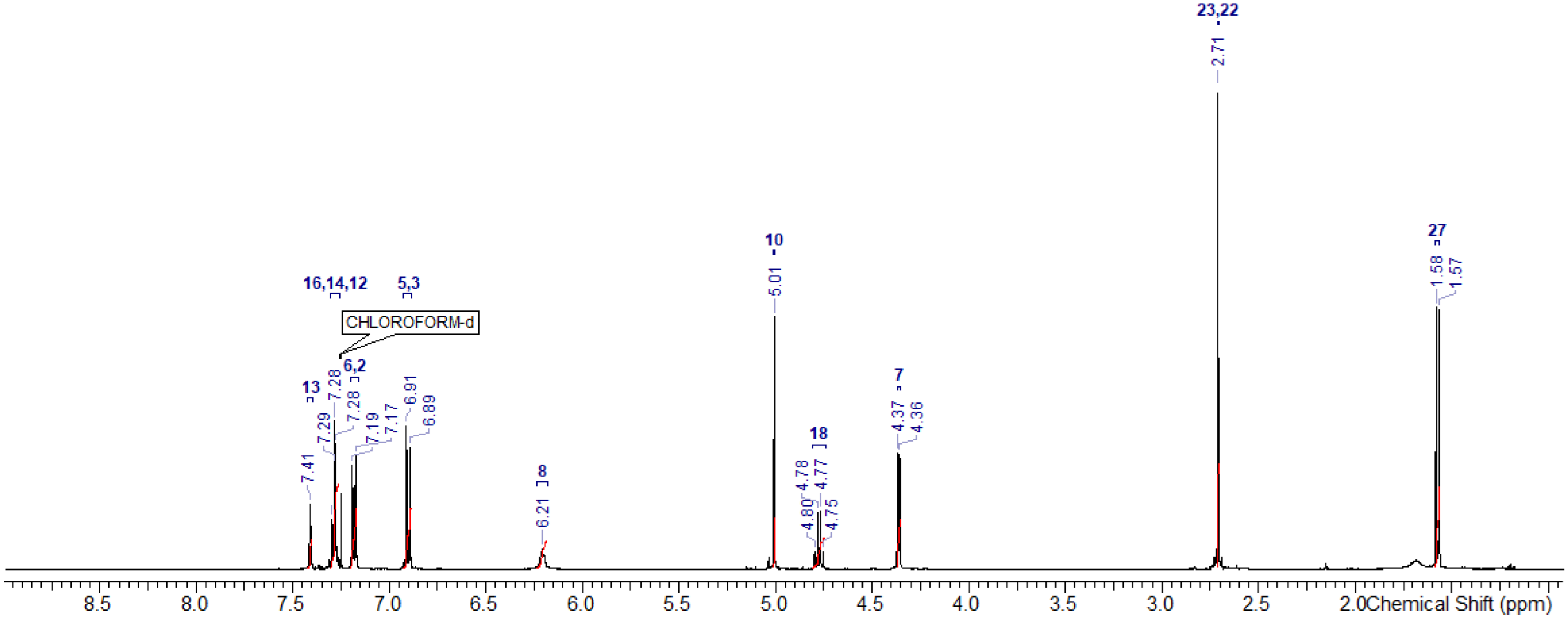

_N_-(4-((3-chlorobenzyl)oxy)benzyl)-2-(2,5-dioxopyrrolidin-1-yl)propanamide (10) – ^{13}C NMR

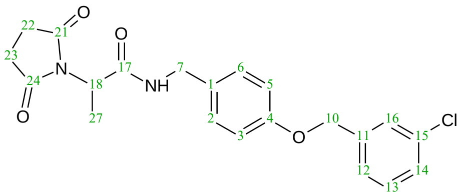

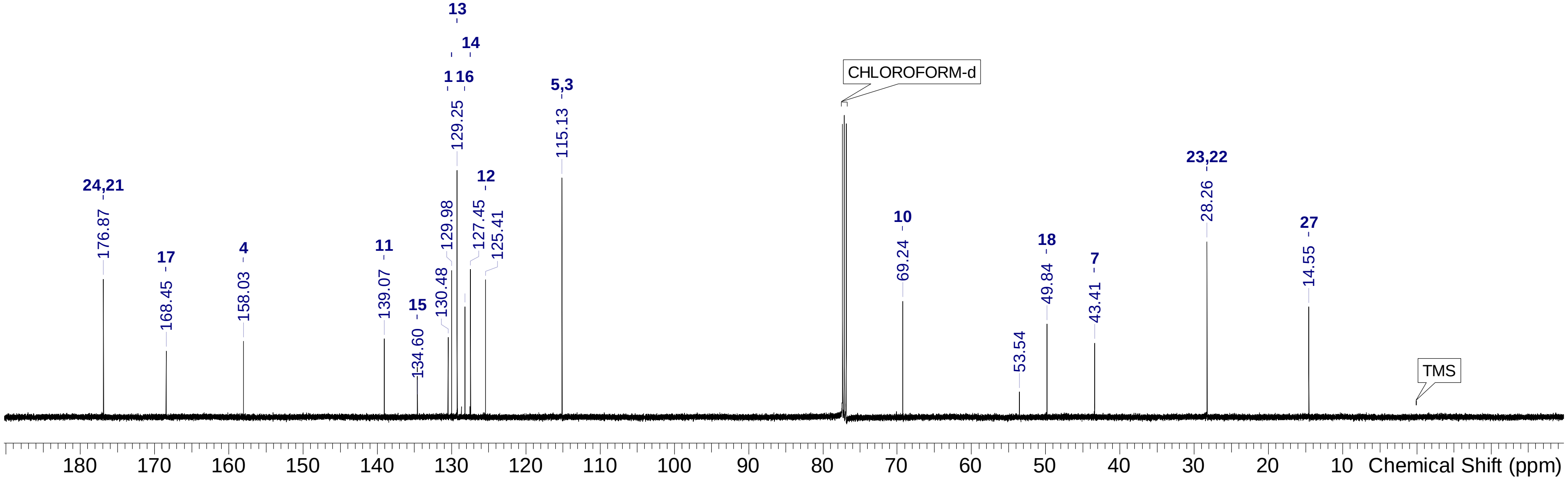

***N*-(4-((4-chlorobenzyl)oxy)benzyl)-2-(2,5-dioxopyrrolidin-1-yl)propanamide (11)** – ^{1}H NMR

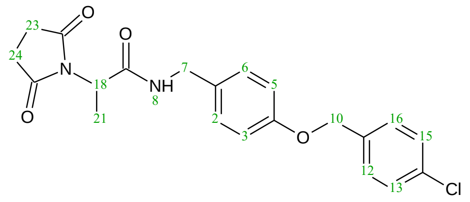

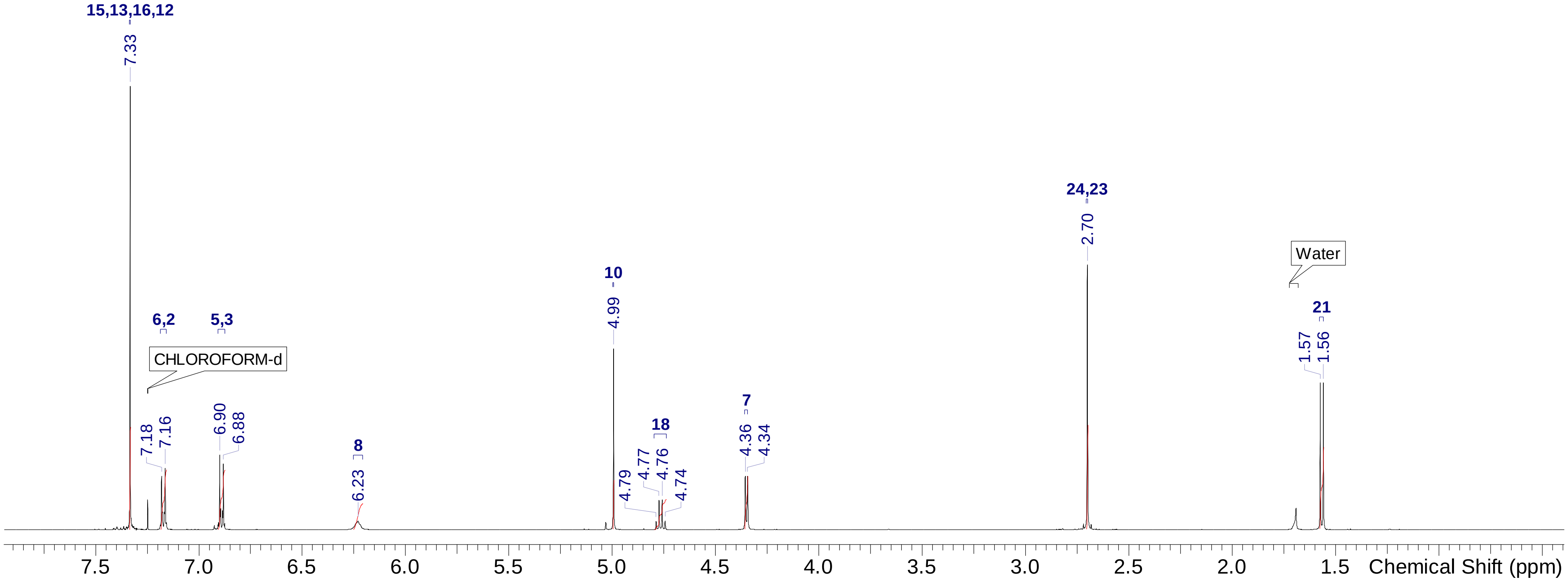

***N*-(4-((4-chlorobenzyl)oxy)benzyl)-2-(2,5-dioxopyrrolidin-1-yl)propanamide (11)** – ^{13}C NMR

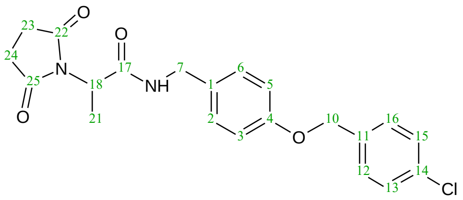

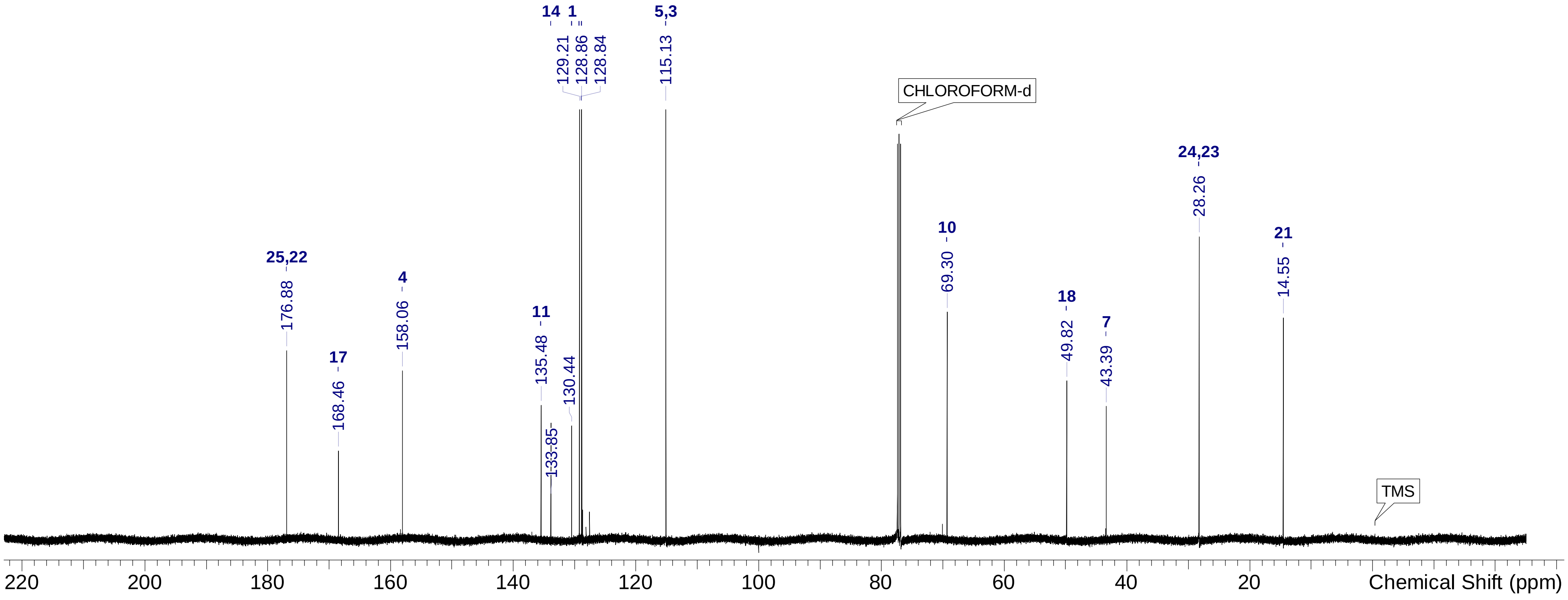

2-(2,5-dioxopyrrolidin-1-yl)-*N*-(4-((2-(trifluoromethyl)benzyl)oxy)benzyl)propanamide (12) – ^{1}H NMR

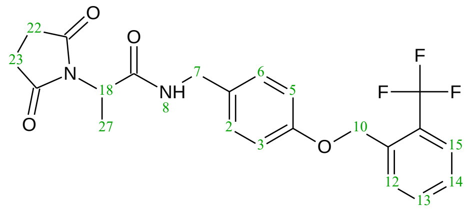

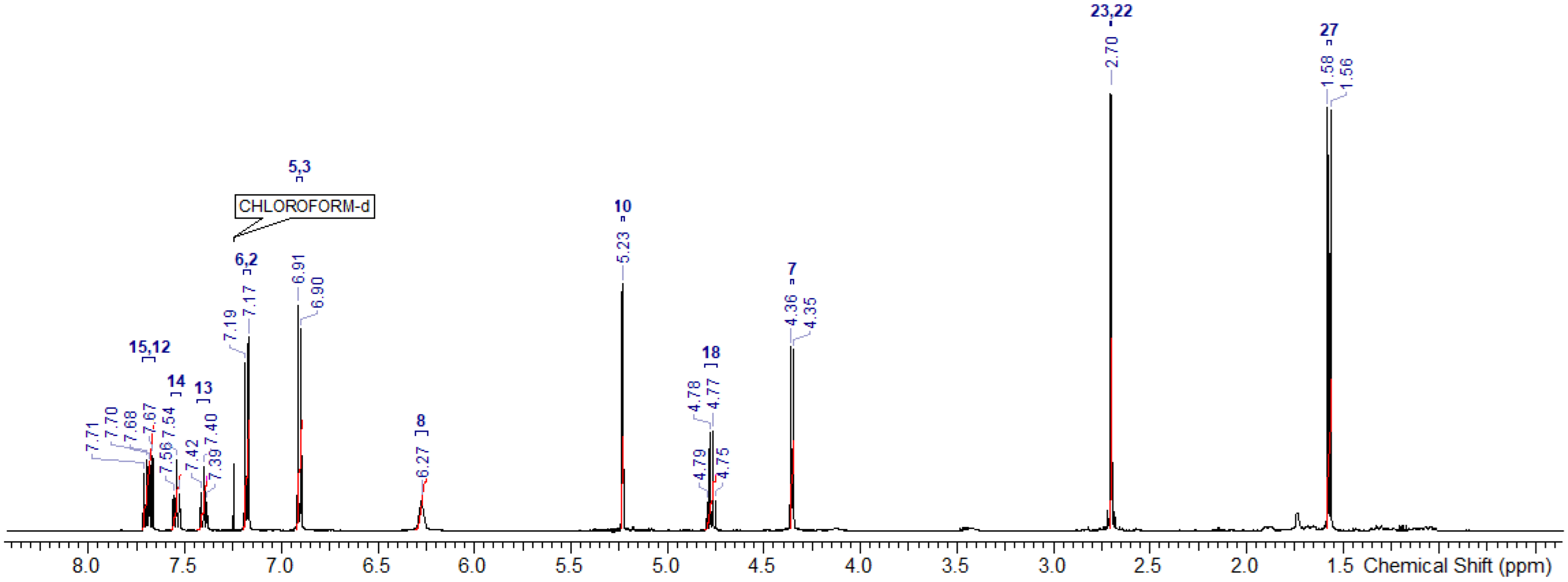

2-(2,5-dioxopyrrolidin-1-yl)-*N*-(4-((2-(trifluoromethyl)benzyl)oxy)benzyl)propanamide (12) – ^{13}C NMR

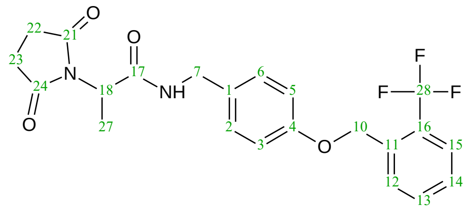

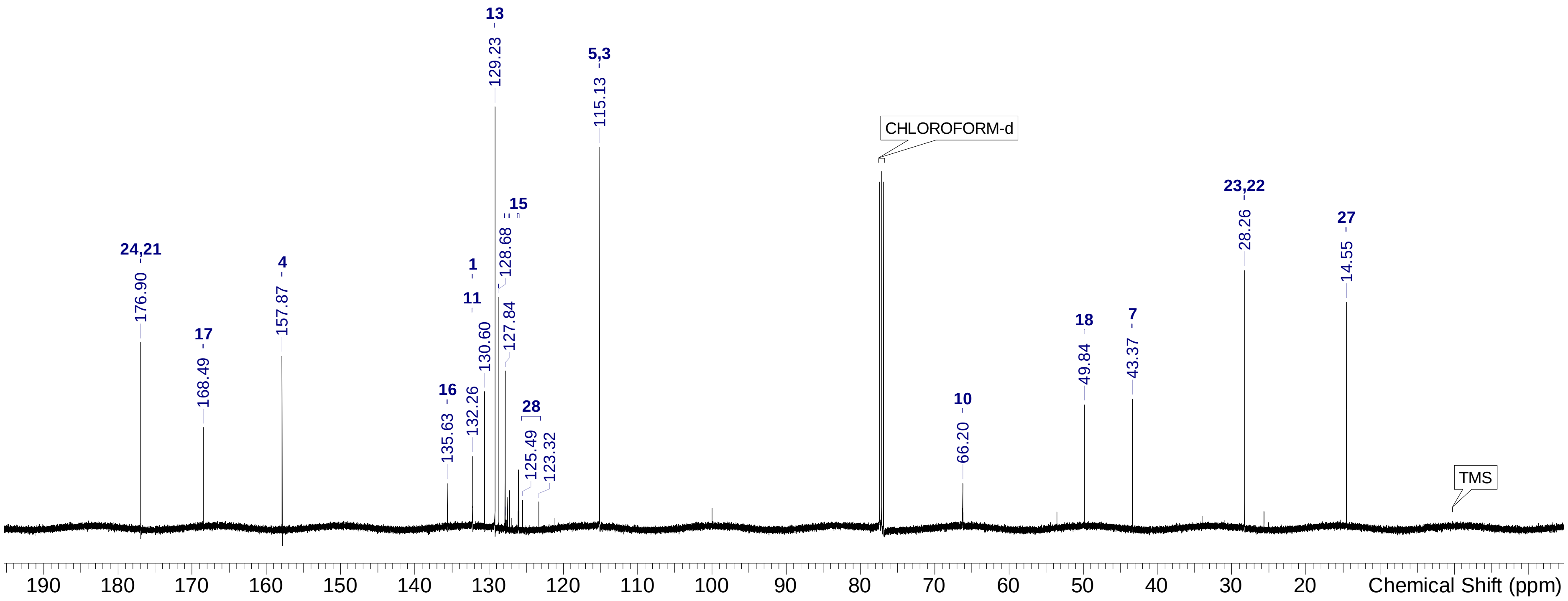

2-(2,5-dioxopyrrolidin-1-yl)-*N*-(4-((3-(trifluoromethyl)benzyl)oxy)benzyl)propanamide (13) – ^{1}H NMR

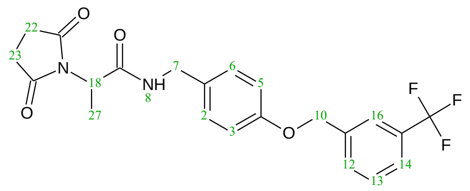

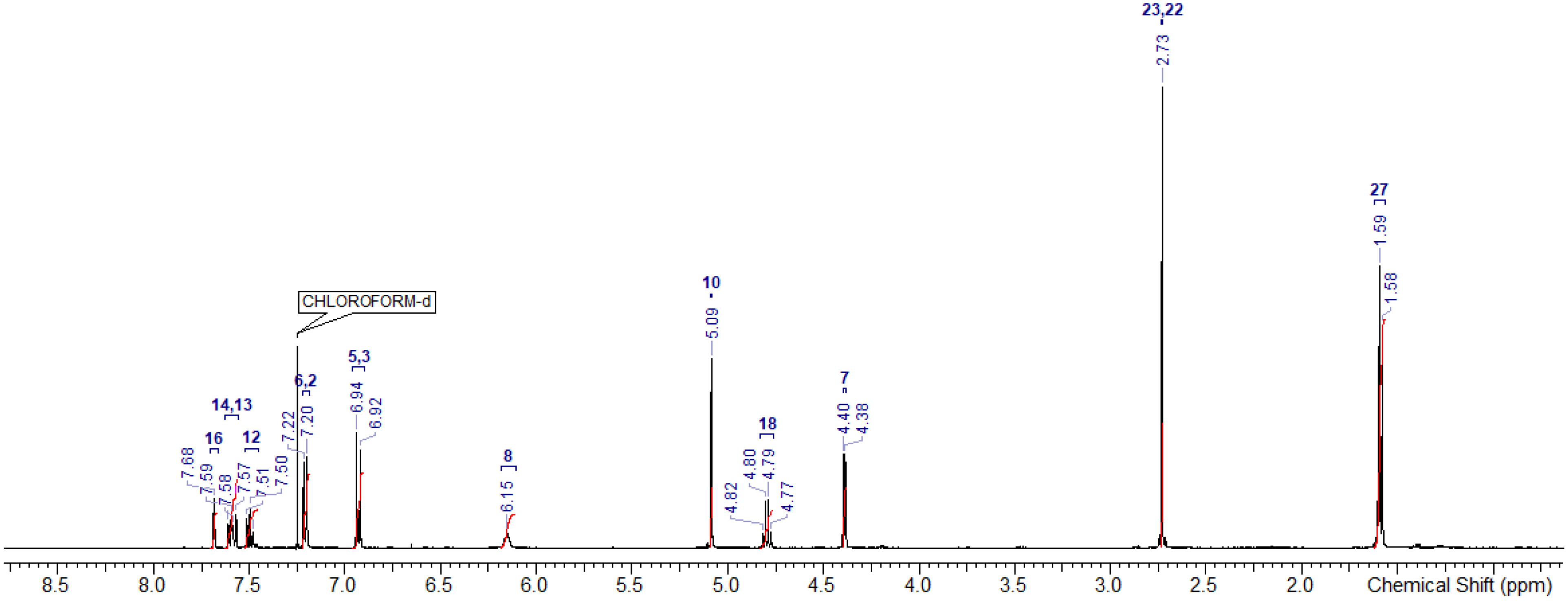

2-(2,5-dioxopyrrolidin-1-yl)-*N*-(4-((3-(trifluoromethyl)benzyl)oxy)benzyl)propanamide (13) – ^{13}C NMR

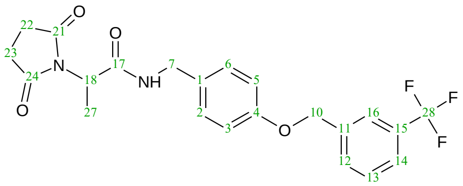

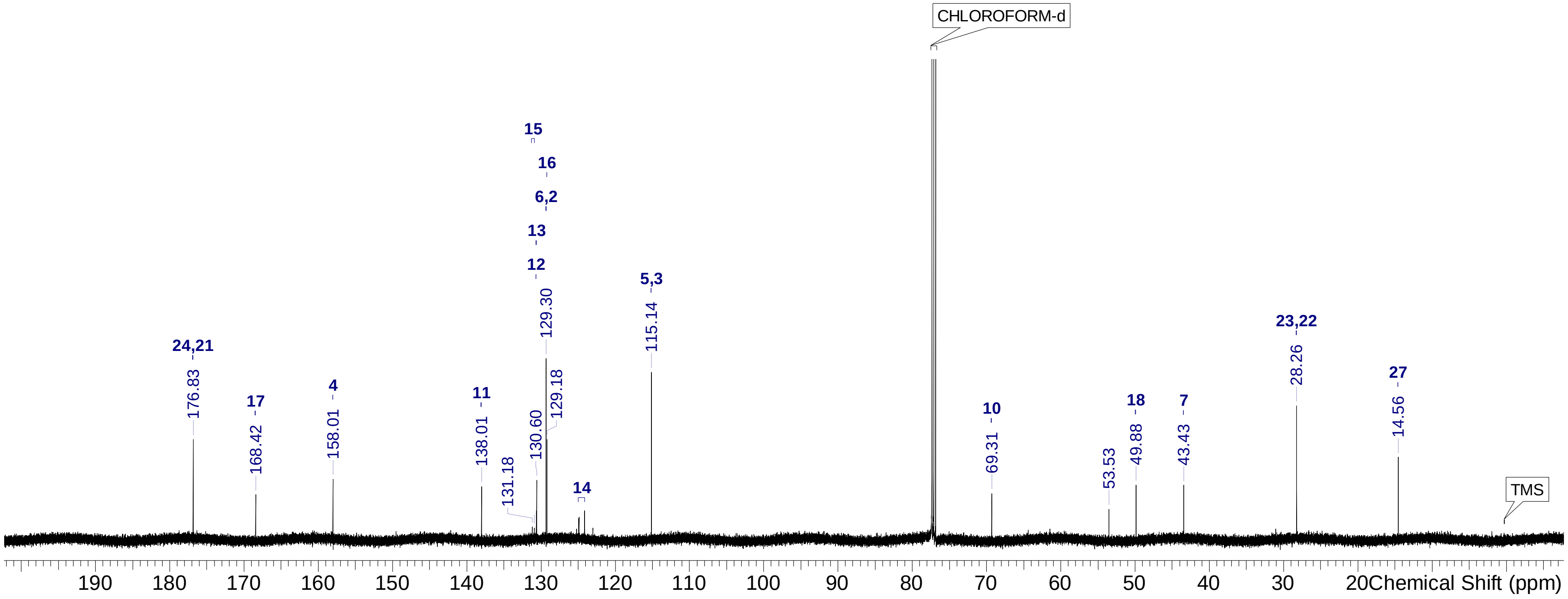

2-(2,5-dioxopyrrolidin-1-yl)-*N*-(4-((4-(trifluoromethyl)benzyl)oxy)benzyl)propanamide (14) – ^{1}H NMR

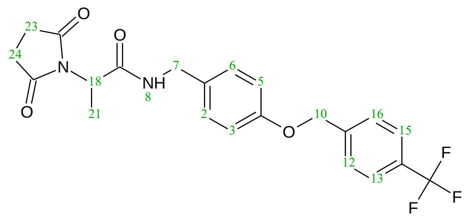

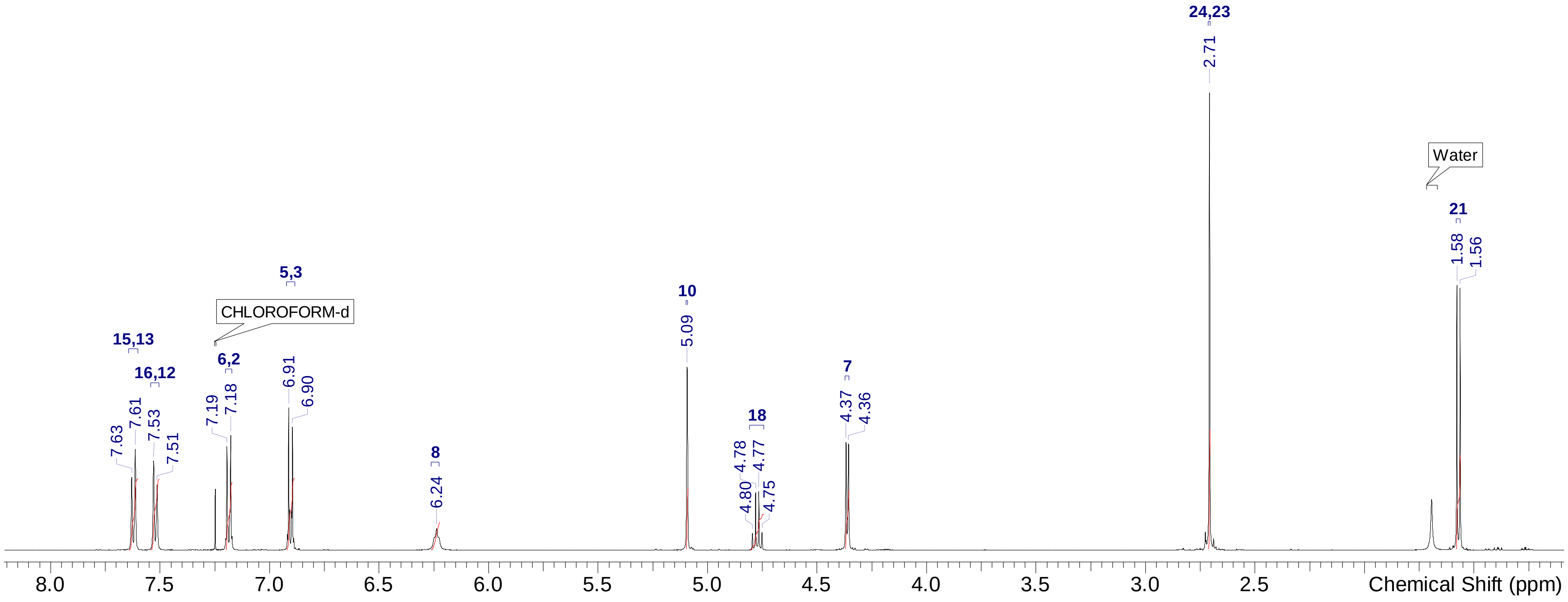

2-(2,5-dioxopyrrolidin-1-yl)-*N*-(4-((4-(trifluoromethyl)benzyl)oxy)benzyl)propanamide (14) – ^{13}C NMR

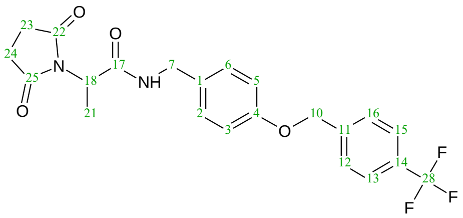

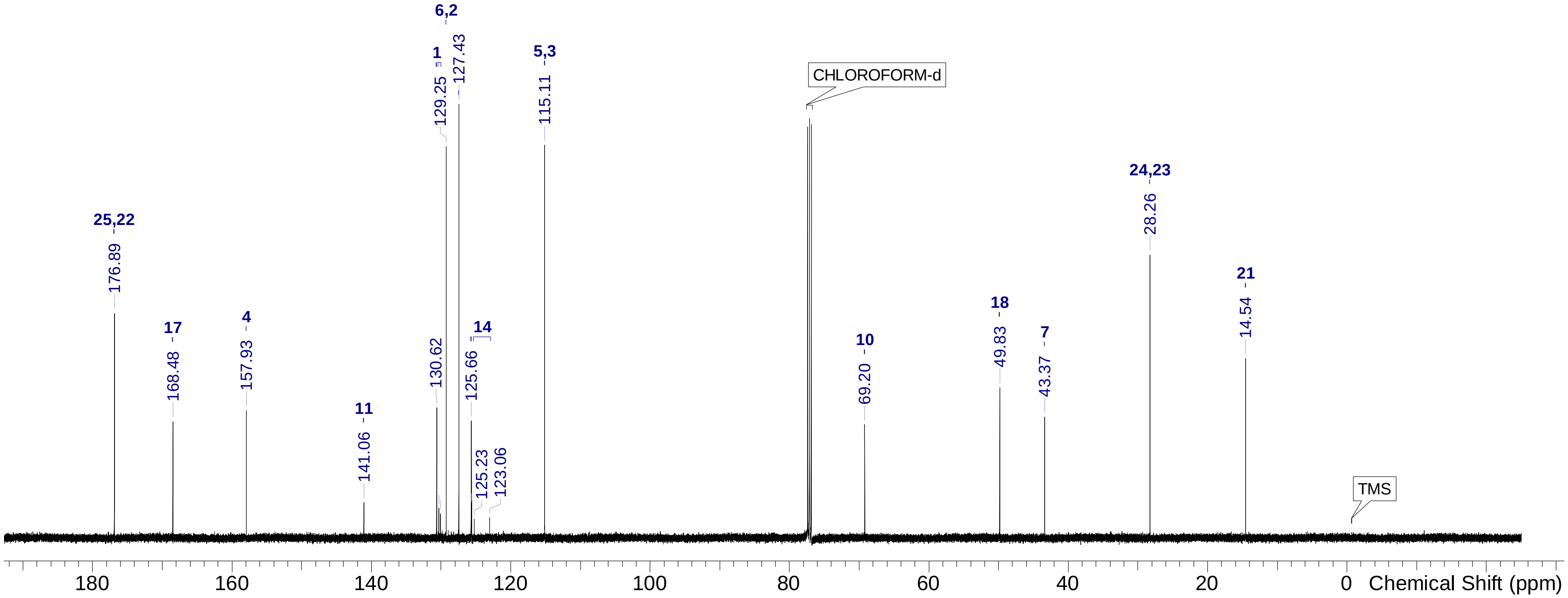

2-(2,5-dioxopyrrolidin-1-yl)-*N*-(4-((2-(trifluoromethoxy)benzyl)oxy)benzyl)propanamide (15) – ^{1}H NMR

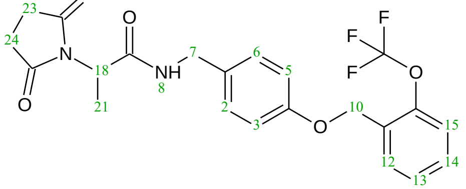

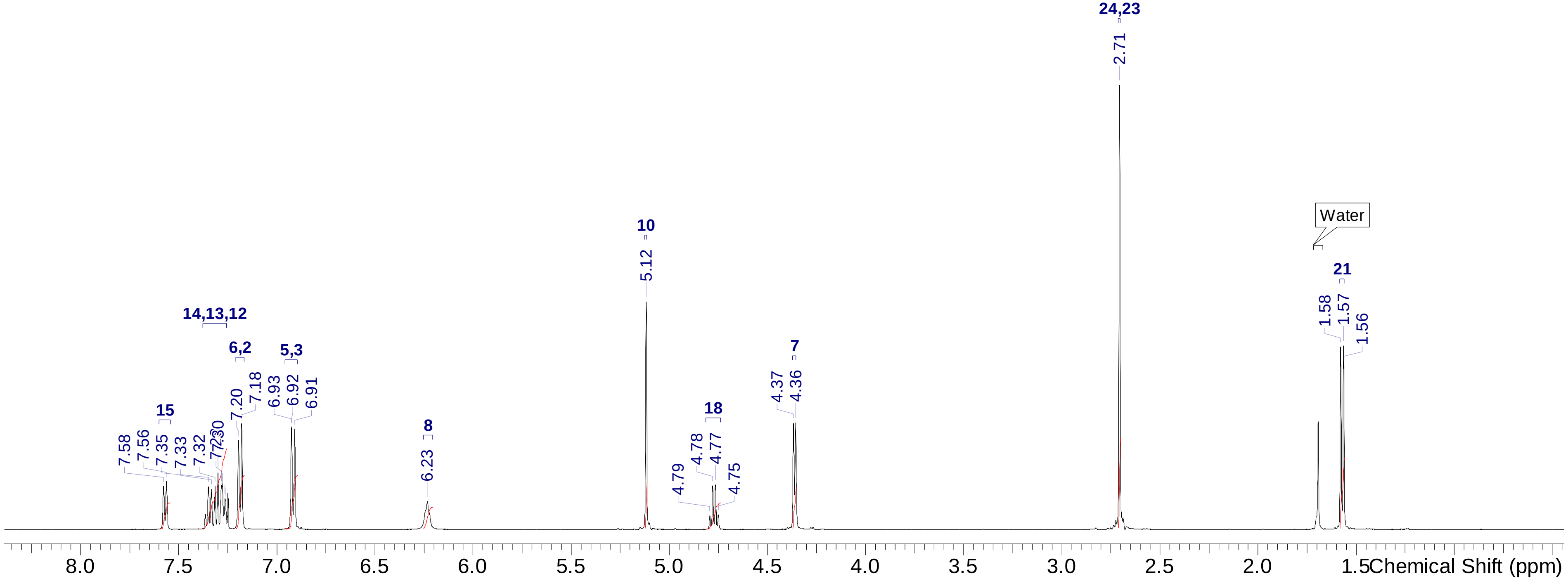

2-(2,5-dioxopyrrolidin-1-yl)-*N*-(4-((2-(trifluoromethoxy)benzyl)oxy)benzyl)propanamide (15) – ^{13}C NMR

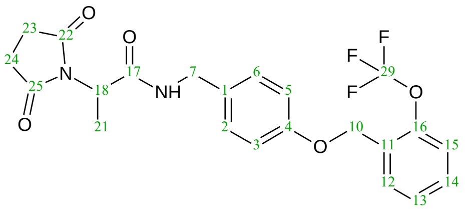

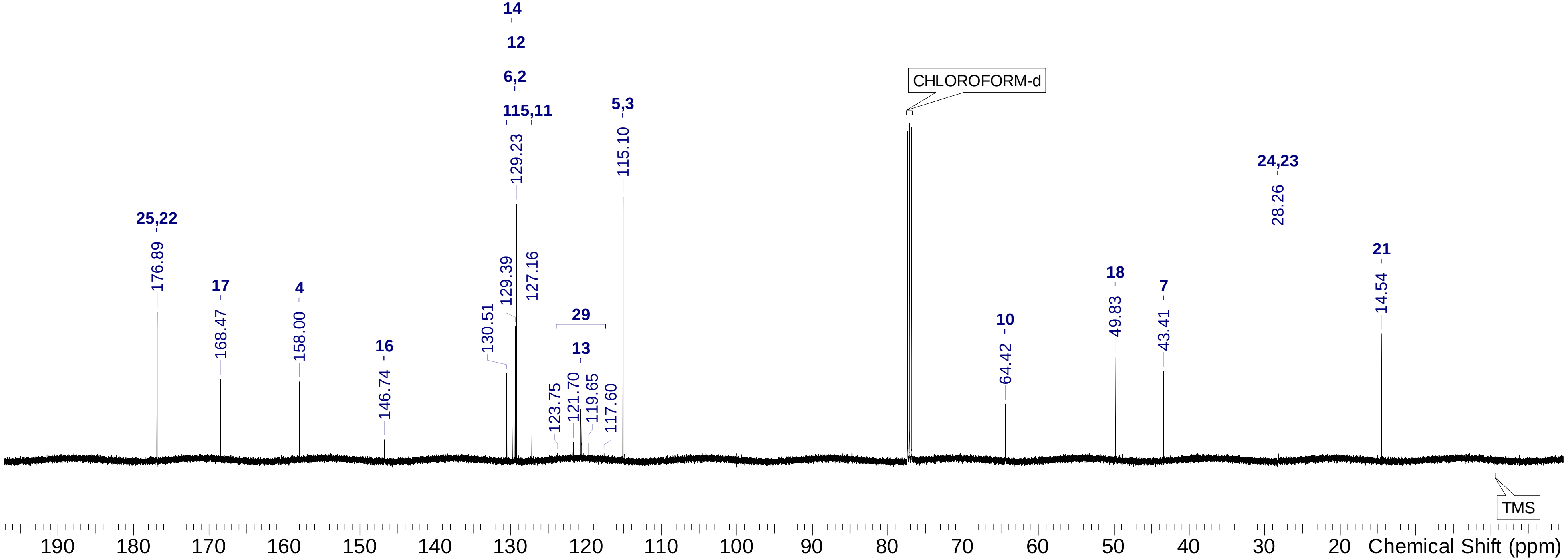

2-(2,5-dioxopyrrolidin-1-yl)-*N*-(4-((3-(trifluoromethoxy)benzyl)oxy)benzyl)propanamide (16) – ^{1}H NMR

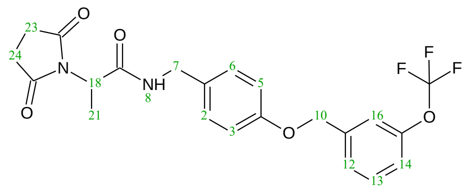

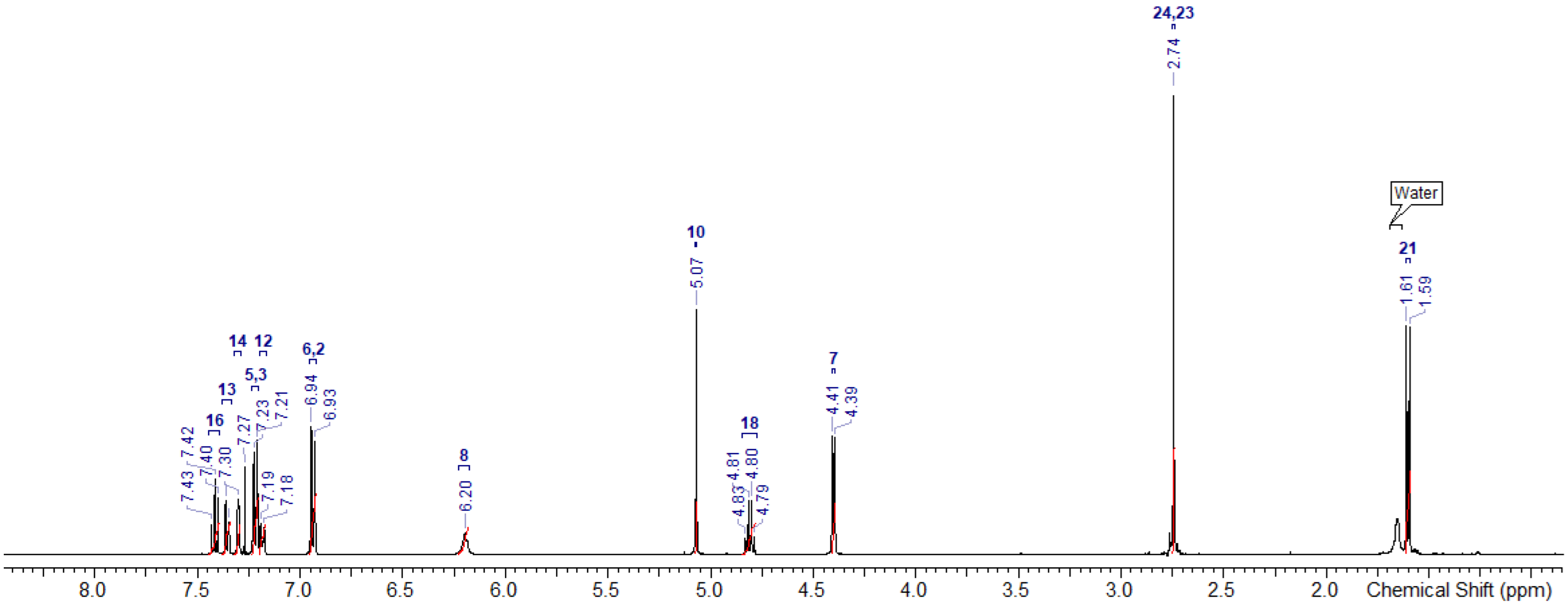

2-(2,5-dioxopyrrolidin-1-yl)-*N*-(4-((3-(trifluoromethoxy)benzyl)oxy)benzyl)propanamide (16) – ^{13}C NMR

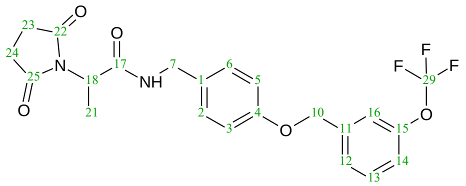

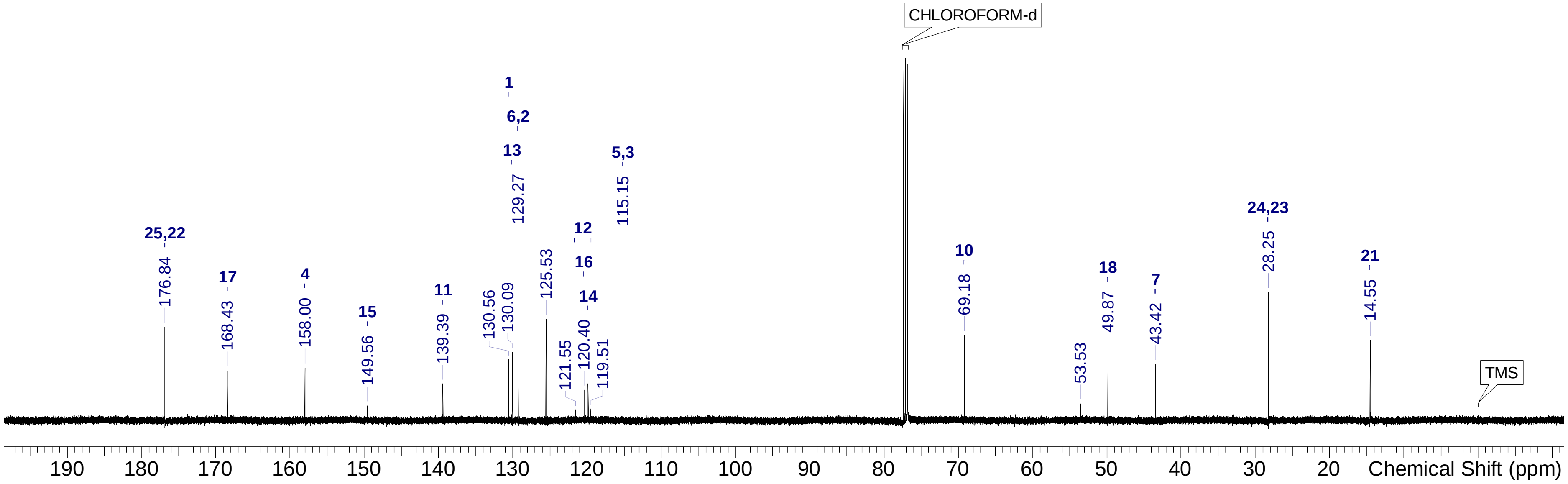

2-(2,5-dioxopyrrolidin-1-yl)-*N*-(4-((4-(trifluoromethoxy)benzyl)oxy)benzyl)propanamide (17) – ^{1}H NMR

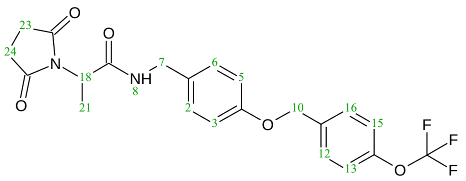

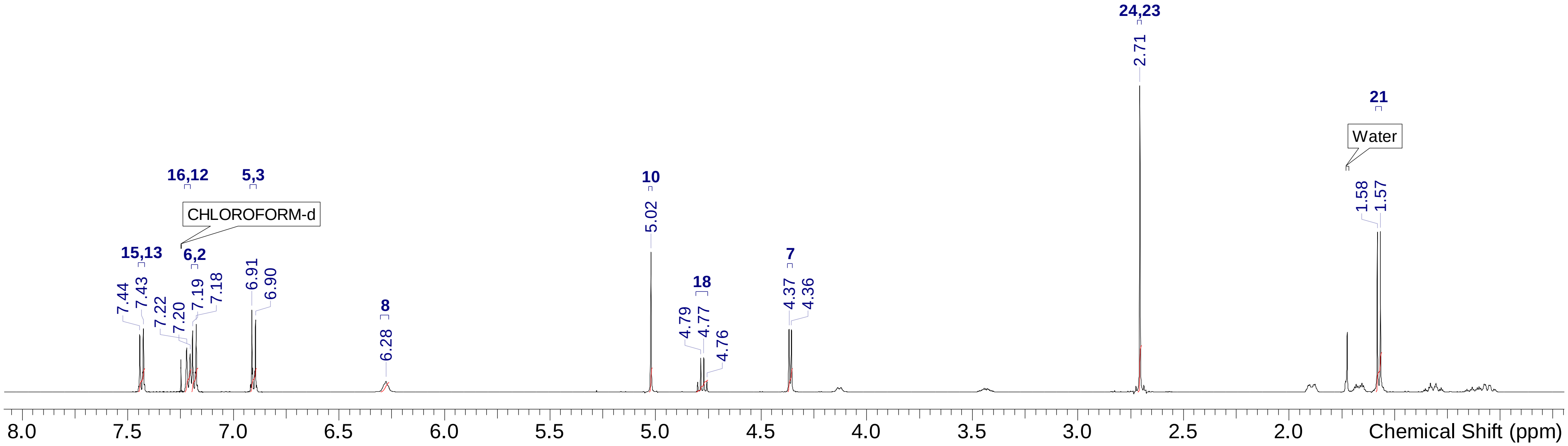

2-(2,5-dioxopyrrolidin-1-yl)-*N*-(4-((4-(trifluoromethoxy)benzyl)oxy)benzyl)propanamide (17) – ^{13}C NMR

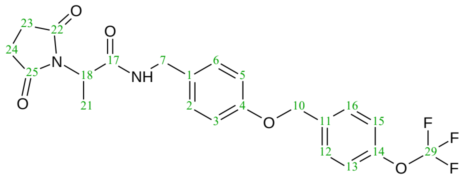

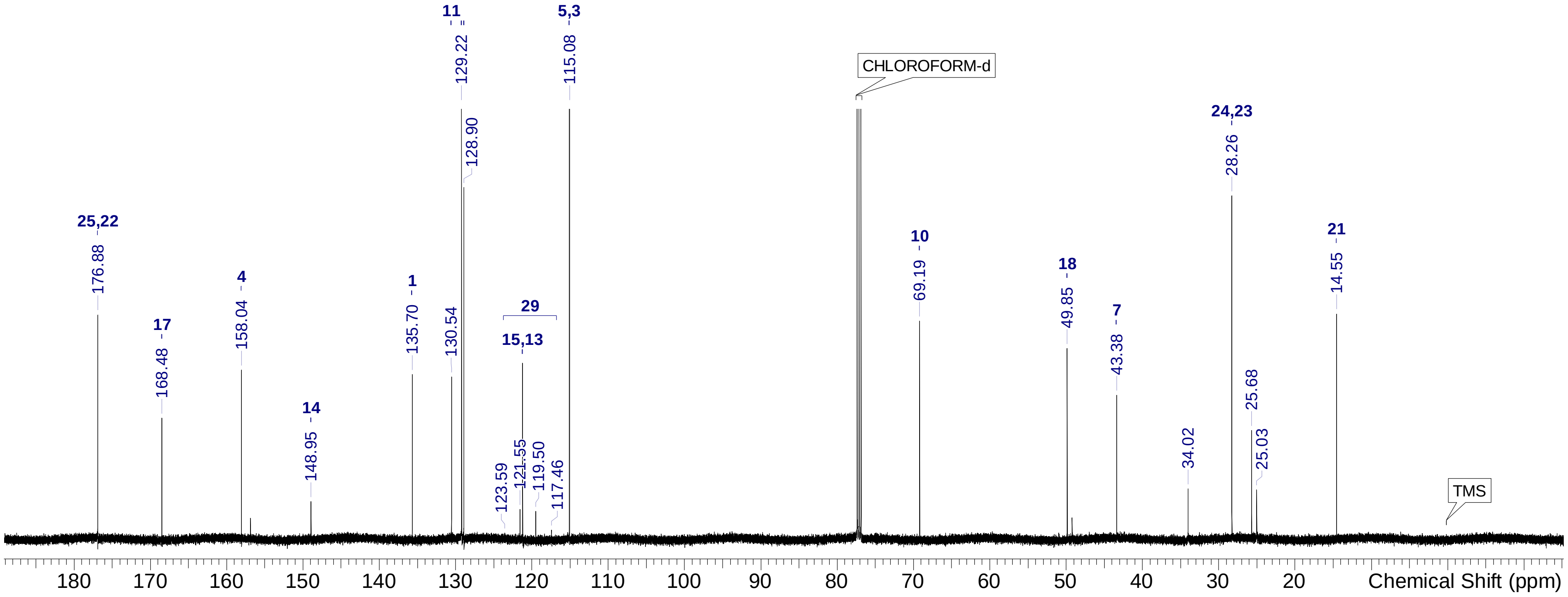

2-(2,5-dioxopyrrolidin-1-yl)-*N*-(4-((2-methylbenzyl)oxy)benzyl)propanamide (18) – ^{1}H NMR

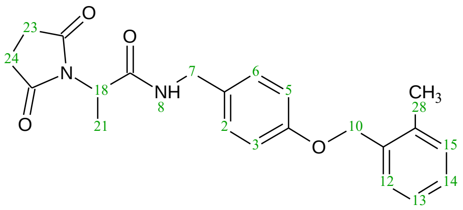

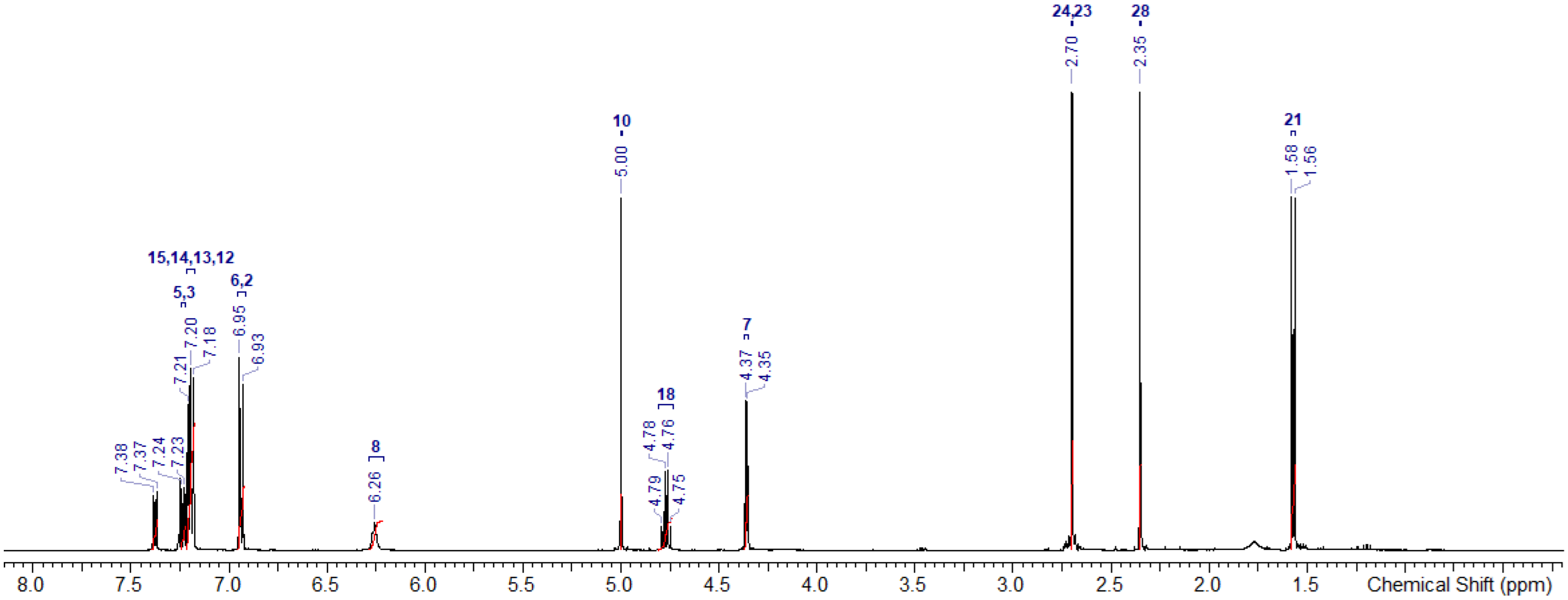

2-(2,5-dioxopyrrolidin-1-yl)-*N*-(4-((2-methylbenzyl)oxy)benzyl)propanamide (18) – ^{13}C NMR

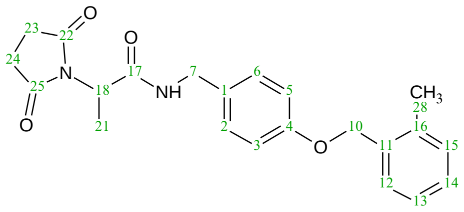

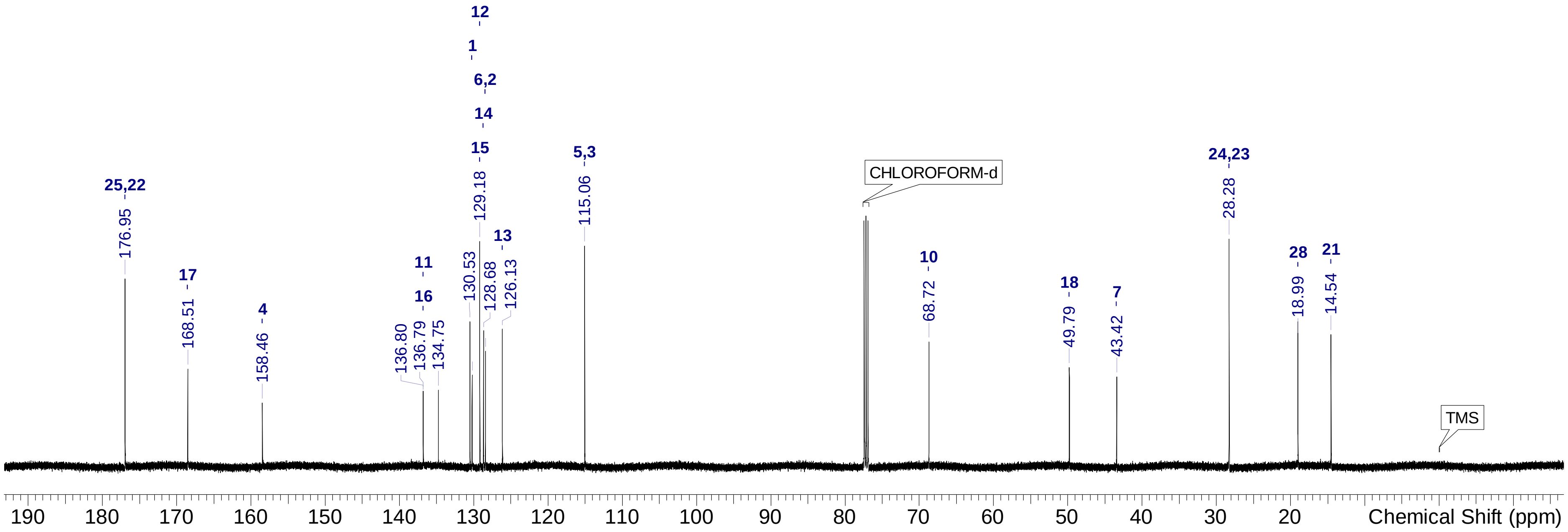

2-(2,5-dioxopyrrolidin-1-yl)-*N*-(4-((3-methylbenzyl)oxy)benzyl)propanamide (19) – ^{1}H NMR

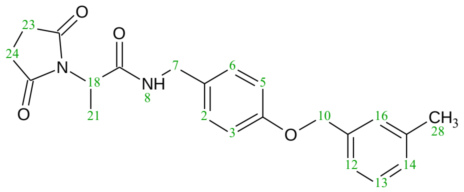

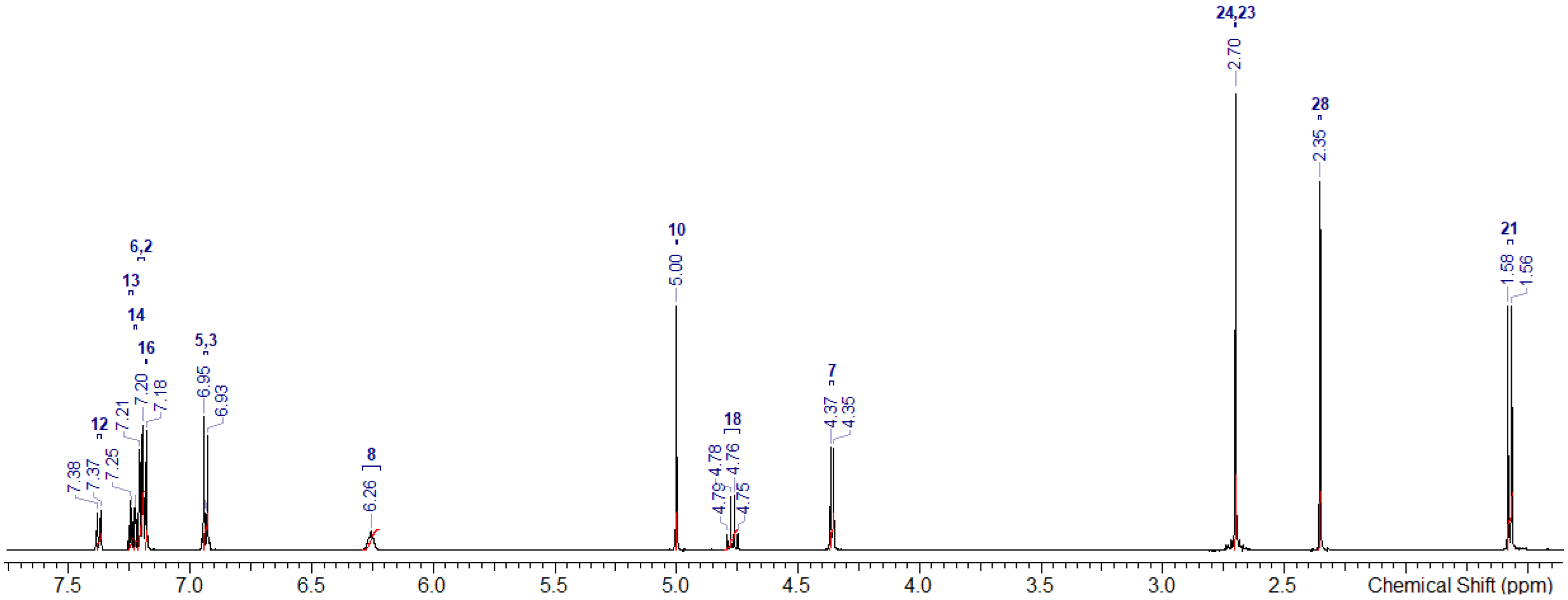

2-(2,5-dioxopyrrolidin-1-yl)-*N*-(4-((3-methylbenzyl)oxy)benzyl)propanamide (19) – ^{13}C NMR

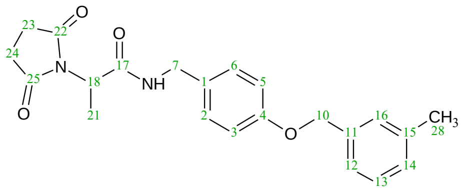

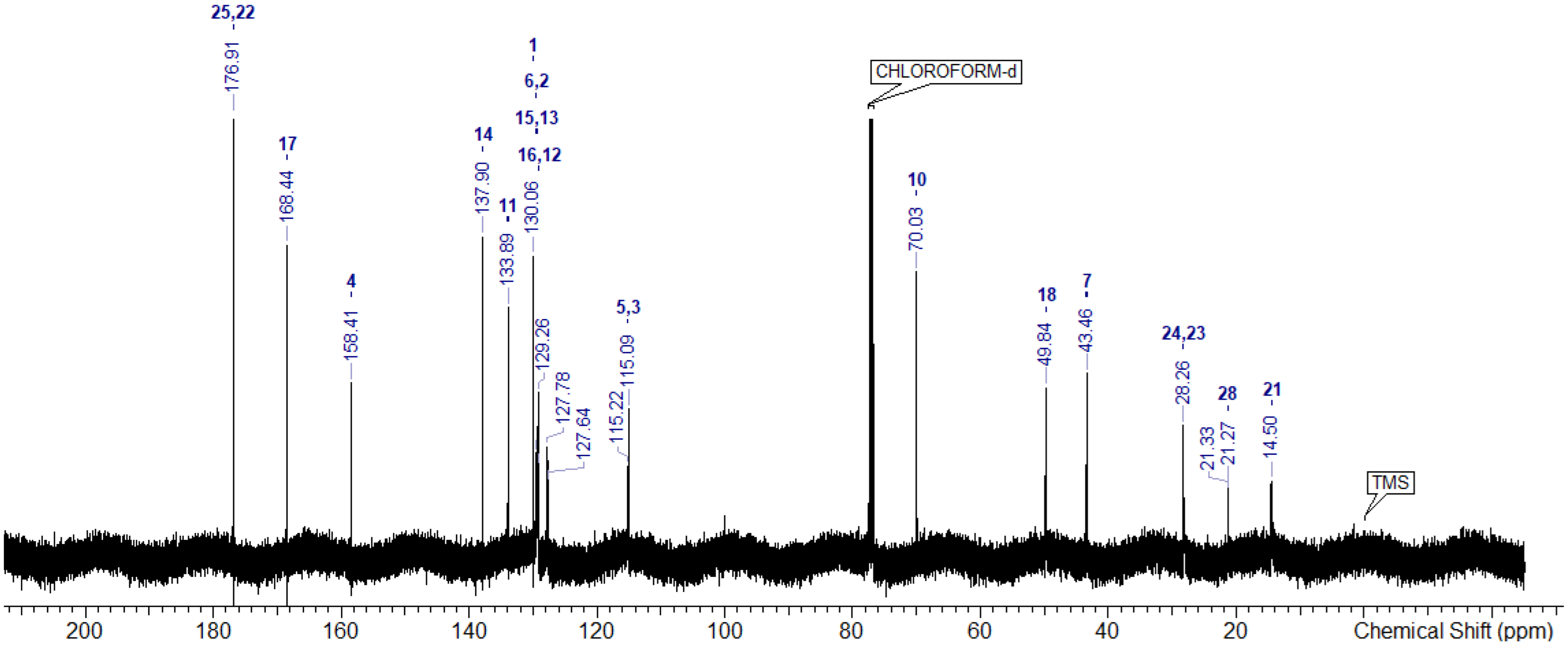

2-(2,5-dioxopyrrolidin-1-yl)-*N*-(4-((4-methylbenzyl)oxy)benzyl)propanamide (20) – ^{1}H NMR

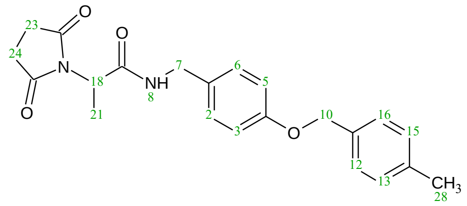

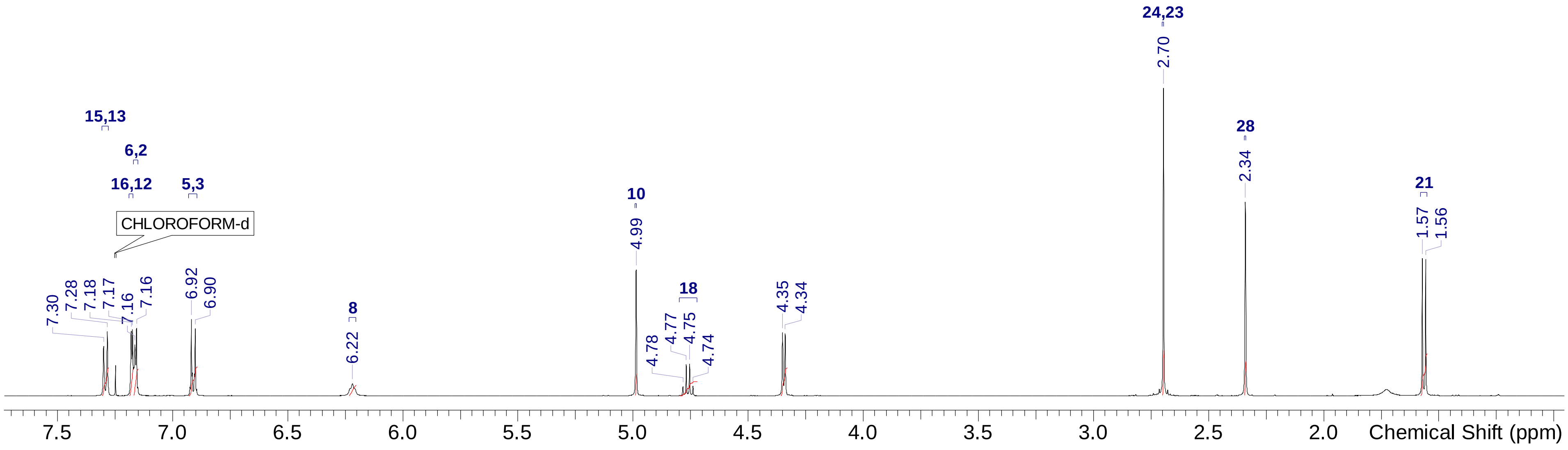

2-(2,5-dioxopyrrolidin-1-yl)-*N*-(4-((4-methylbenzyl)oxy)benzyl)propanamide (20) – ^{13}C NMR

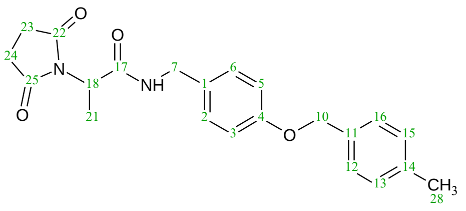

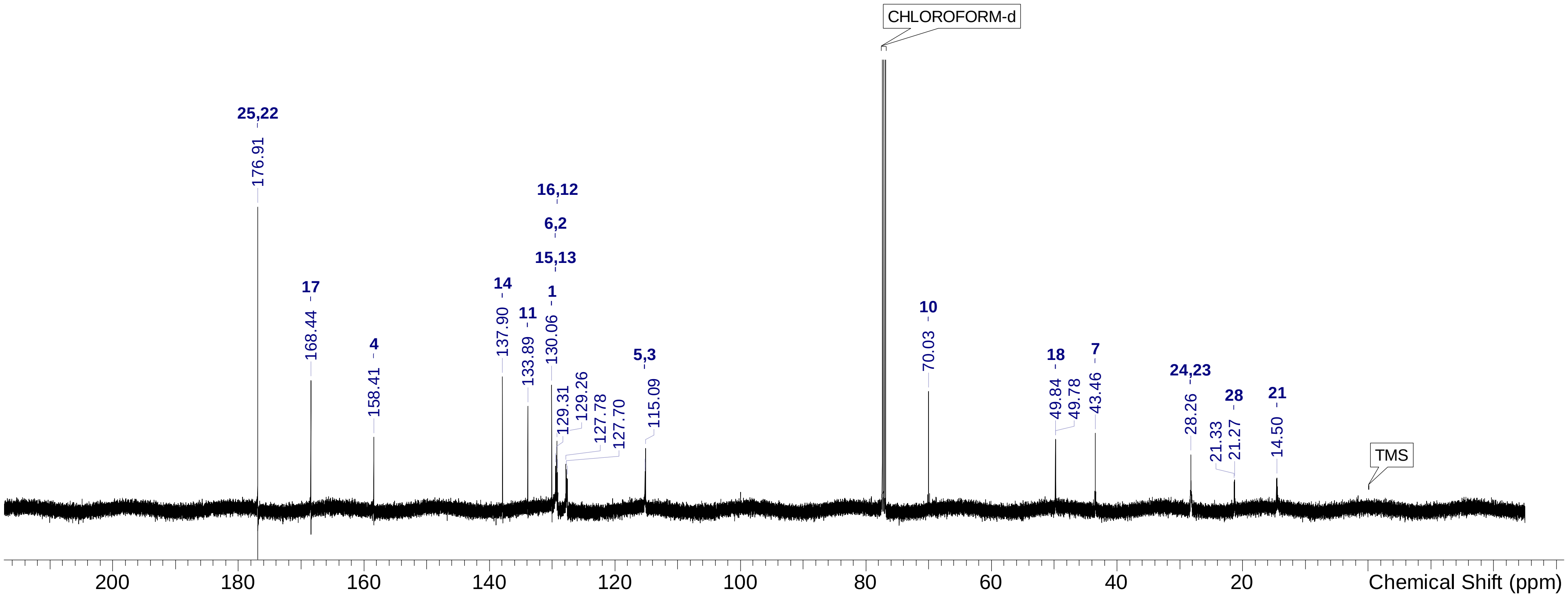

***N*-(4-(benzyloxy)benzyl)-2-(2,5-dioxopyrrolidin-1-yl)-2-methylpropanamide (21)** – ^{1}H NMR

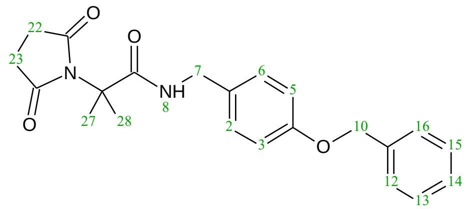

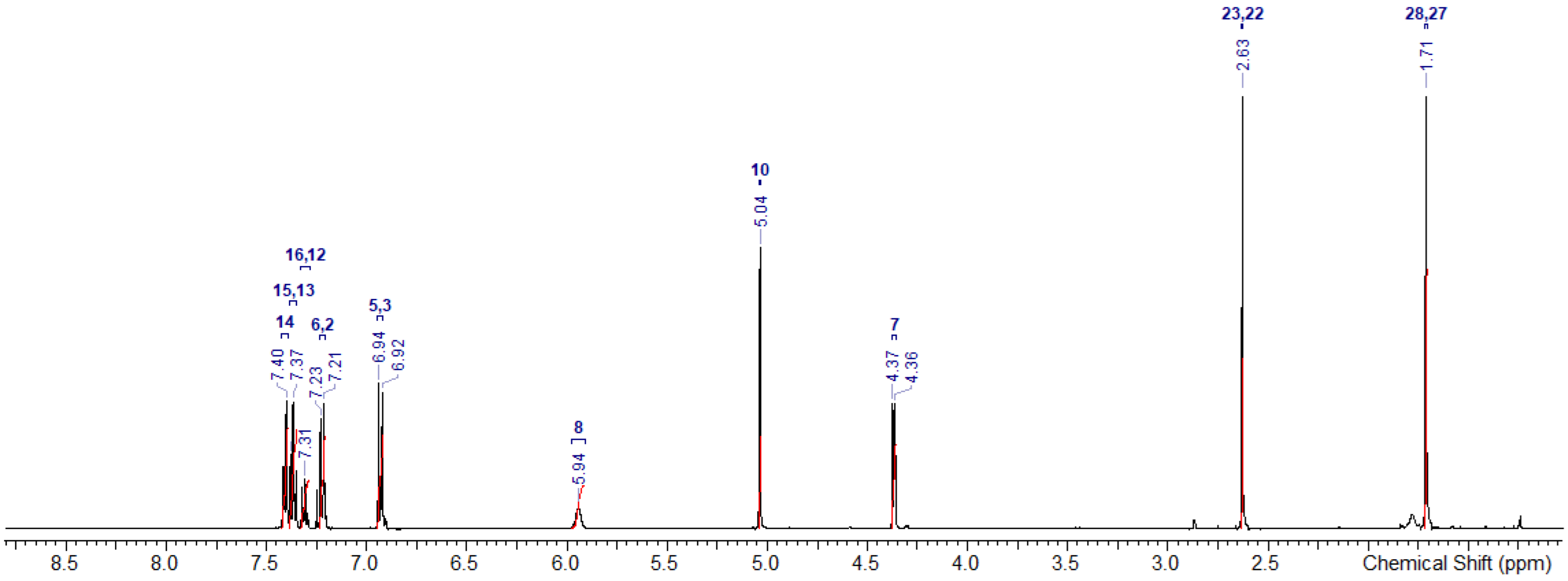

***N*-(4-(benzyloxy)benzyl)-2-(2,5-dioxopyrrolidin-1-yl)-2-methylpropanamide (21)** – ^{13}C NMR

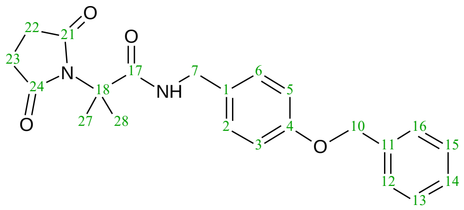

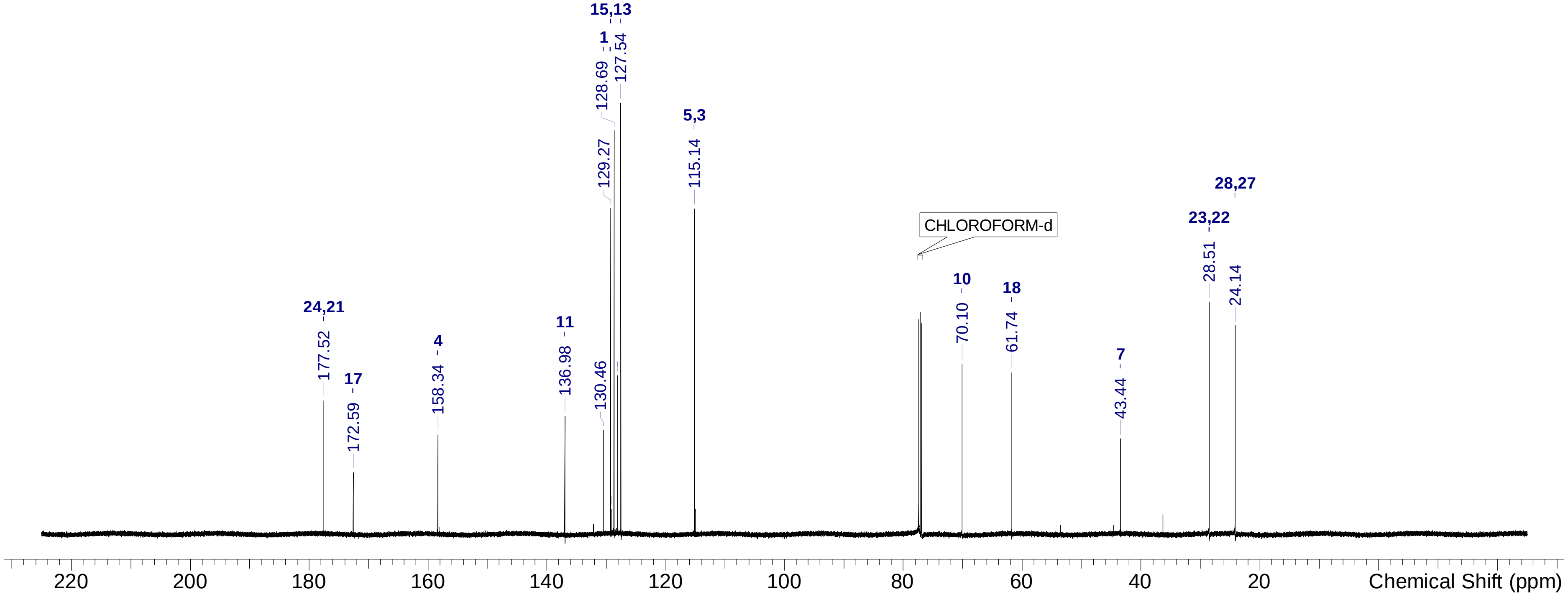

***N*-(3-(benzyloxy)benzyl)-2-(2,5-dioxopyrrolidin-1-yl)propanamide (22)** – ^{1}H NMR

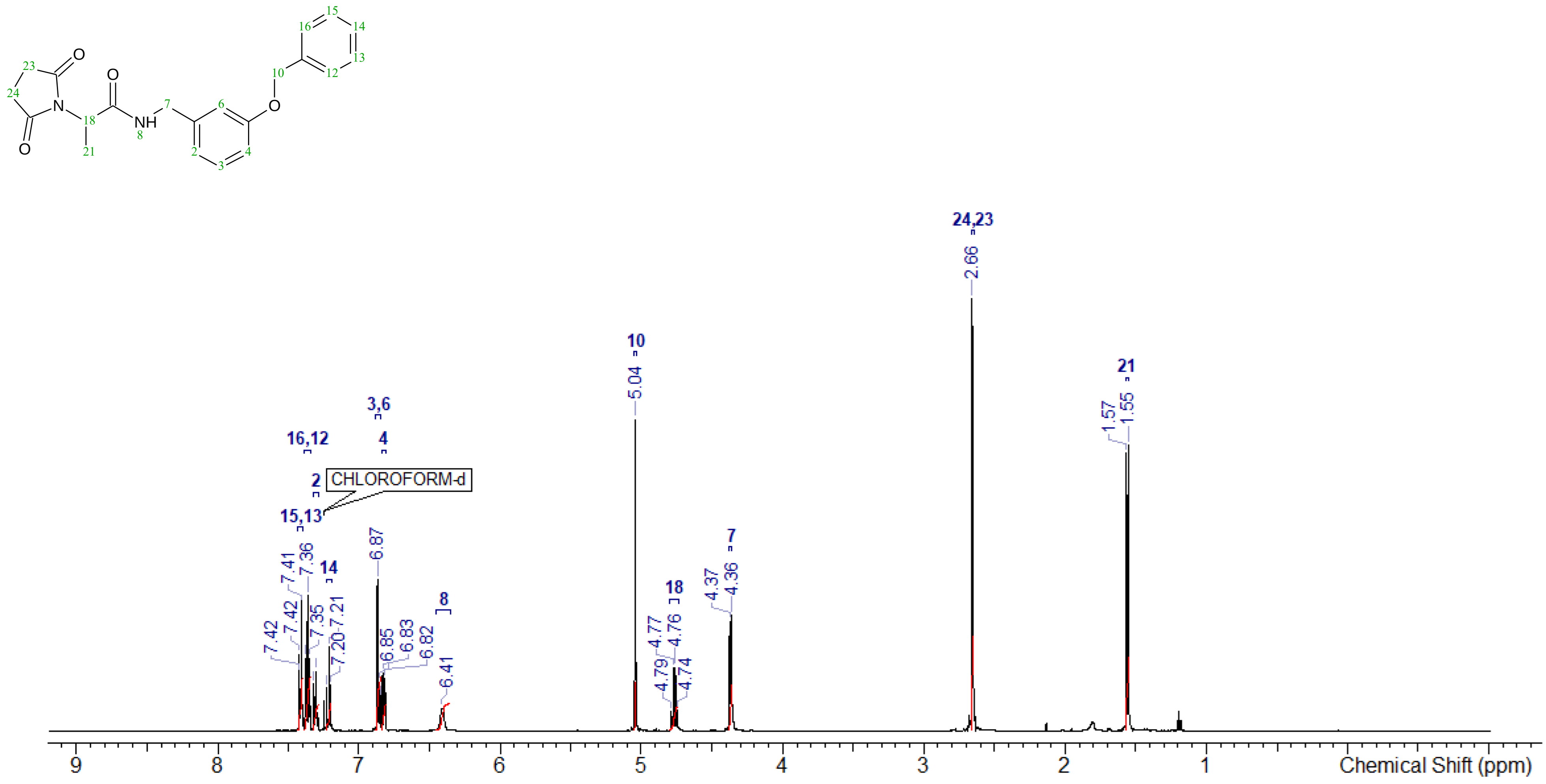

***N*-(3-(benzyloxy)benzyl)-2-(2,5-dioxopyrrolidin-1-yl)propanamide (22)** – ^{13}C NMR

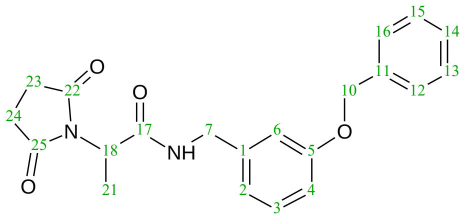

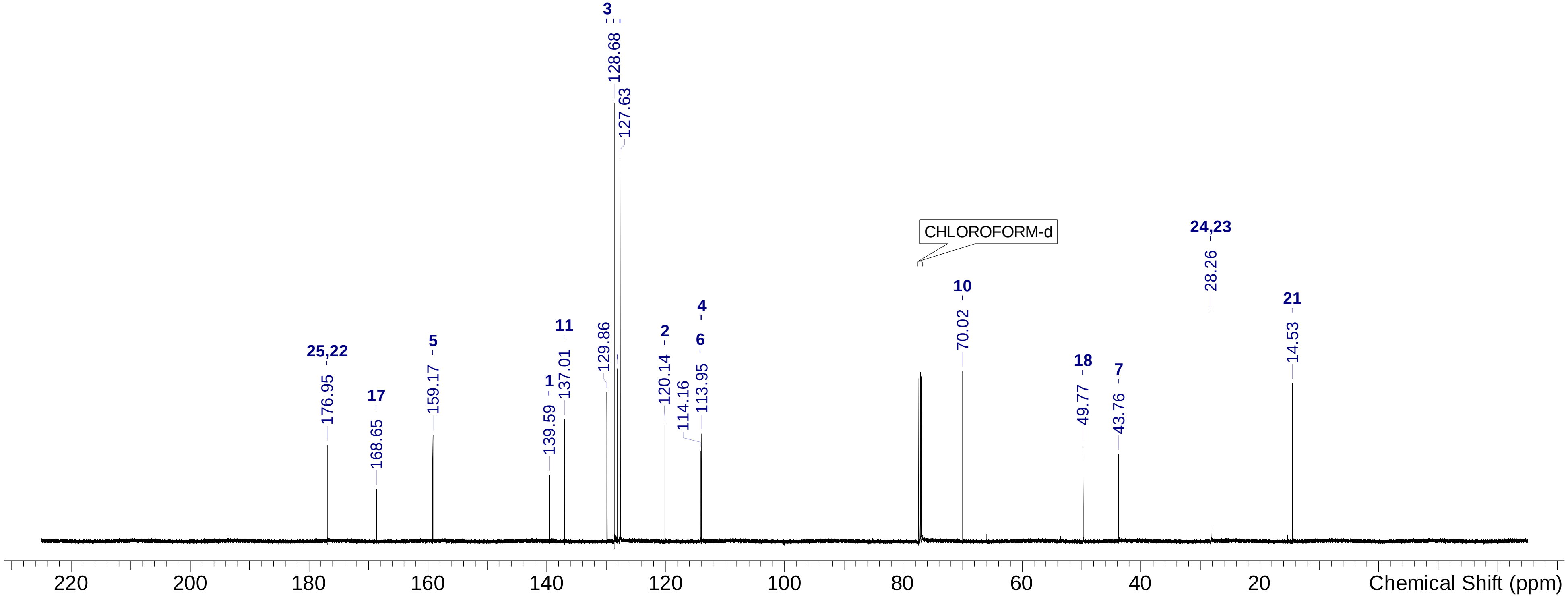

***N*-(2-(benzyloxy)benzyl)-2-(2,5-dioxopyrrolidin-1-yl)propanamide (23)** – ^{1}H NMR

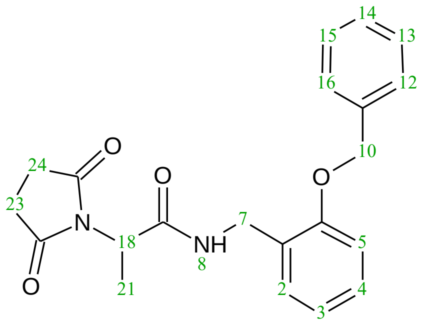

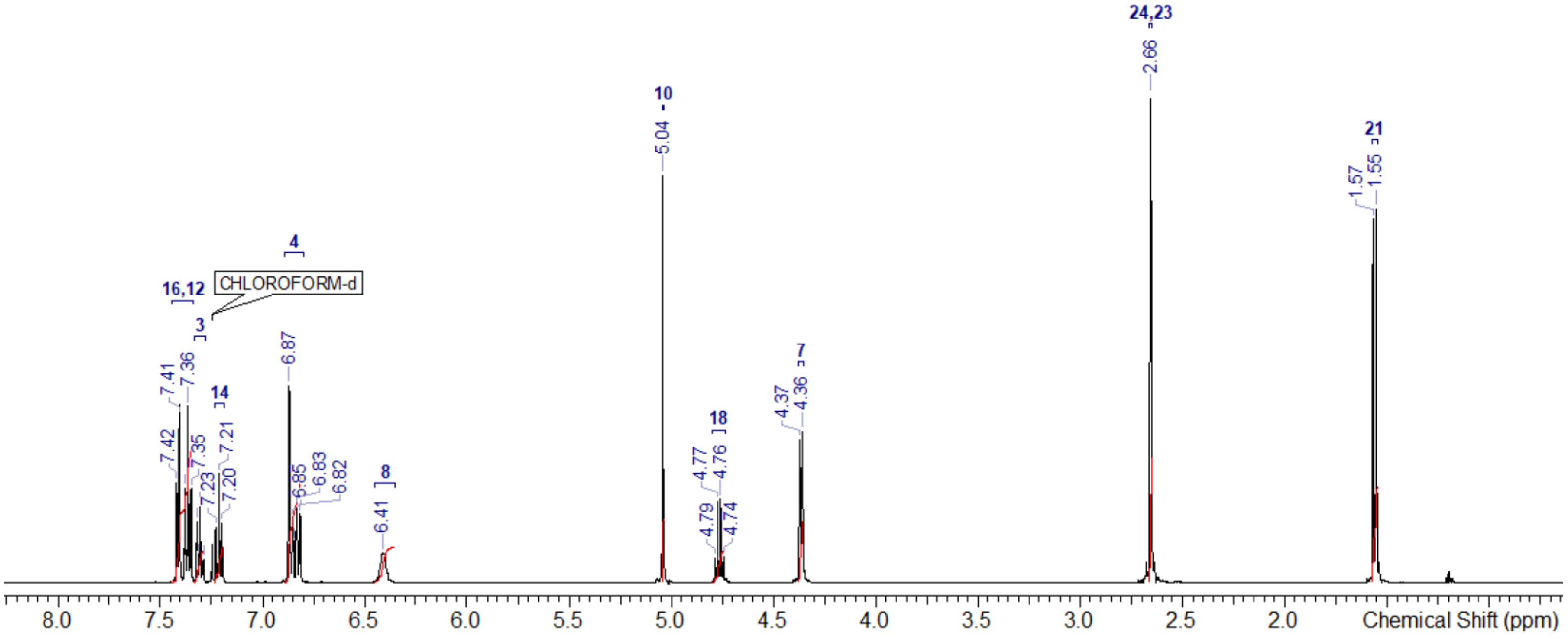

***N*-(2-(benzyloxy)benzyl)-2-(2,5-dioxopyrrolidin-1-yl)propanamide (23)** – ^{13}C NMR

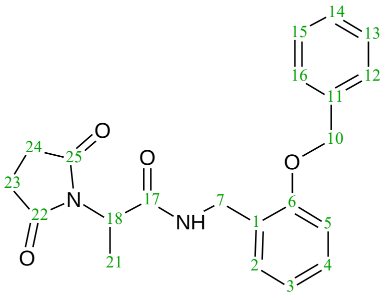

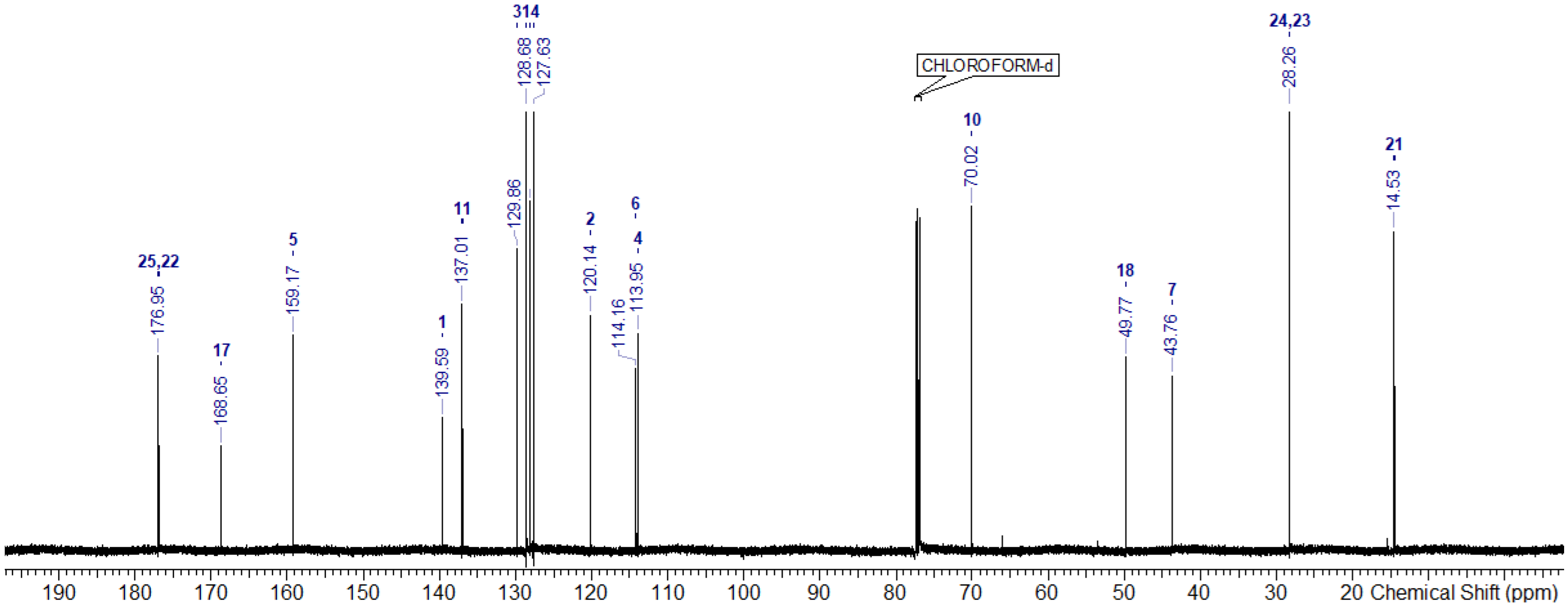

***N*-(4-(benzyloxy)benzyl)-2-(2-oxopyrrolidin-1-yl)propanamide (36)** – ^{1}H NMR

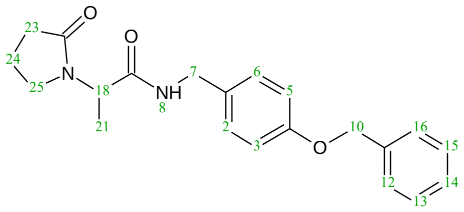

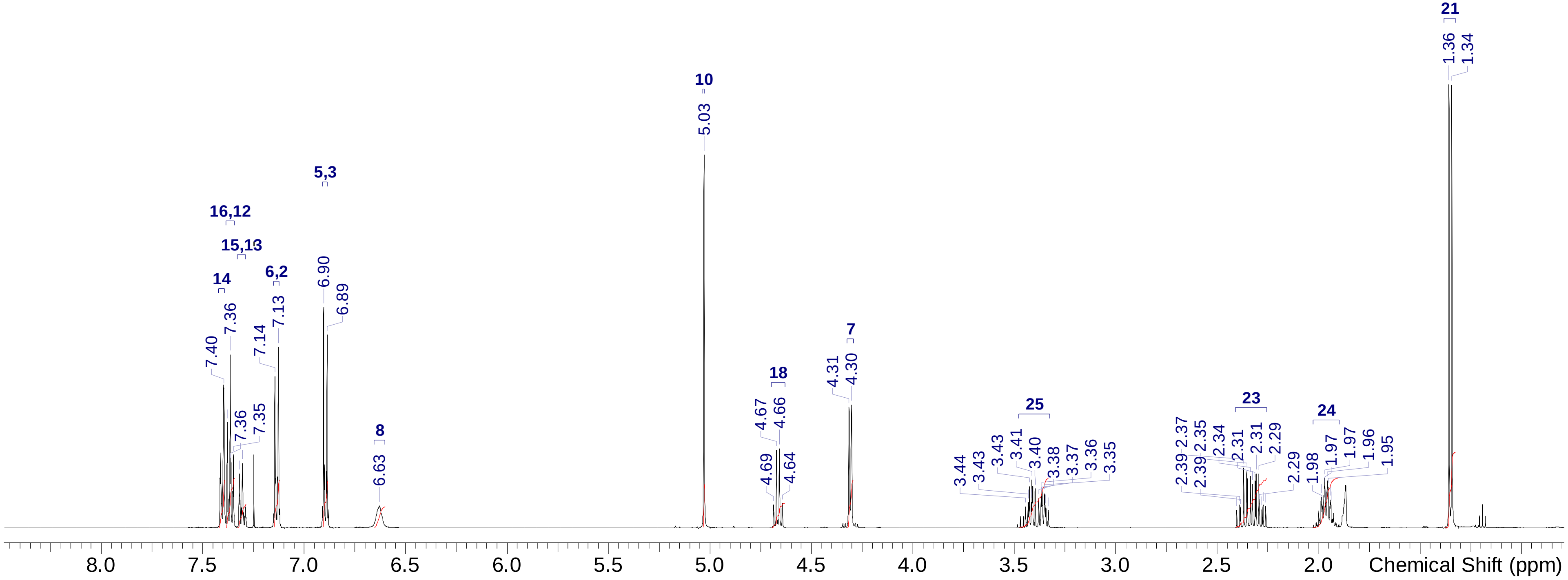

N-(4-(benzyloxy)benzyl)-2-(2-oxopyrrolidin-1-yl)propanamide (36) – ^{13}C NMR

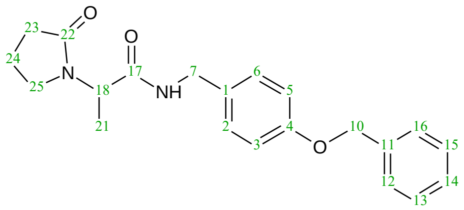

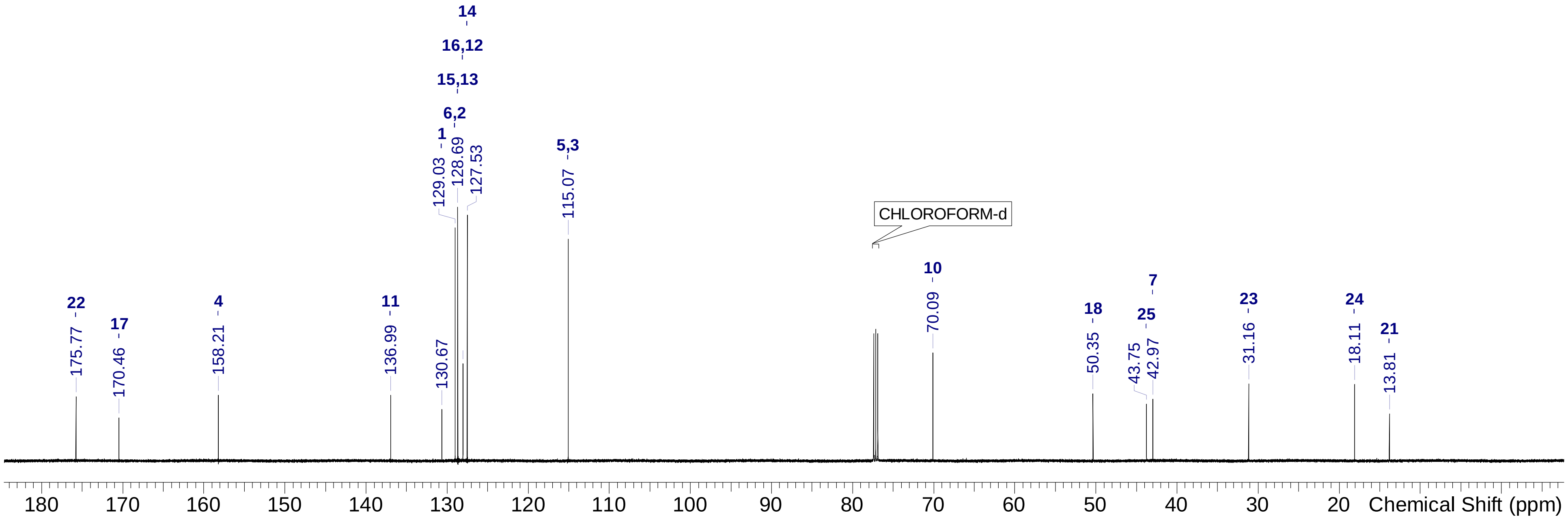

2-(2-oxopyrrolidin-1-yl)-*N*-(4-((2-(trifluoromethyl)benzyl)oxy)benzyl)propanamide (37) – ^{1}H NMR

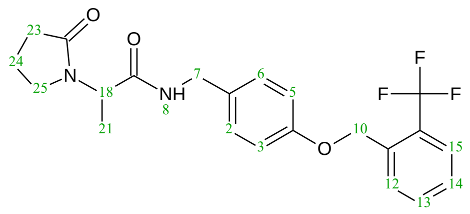

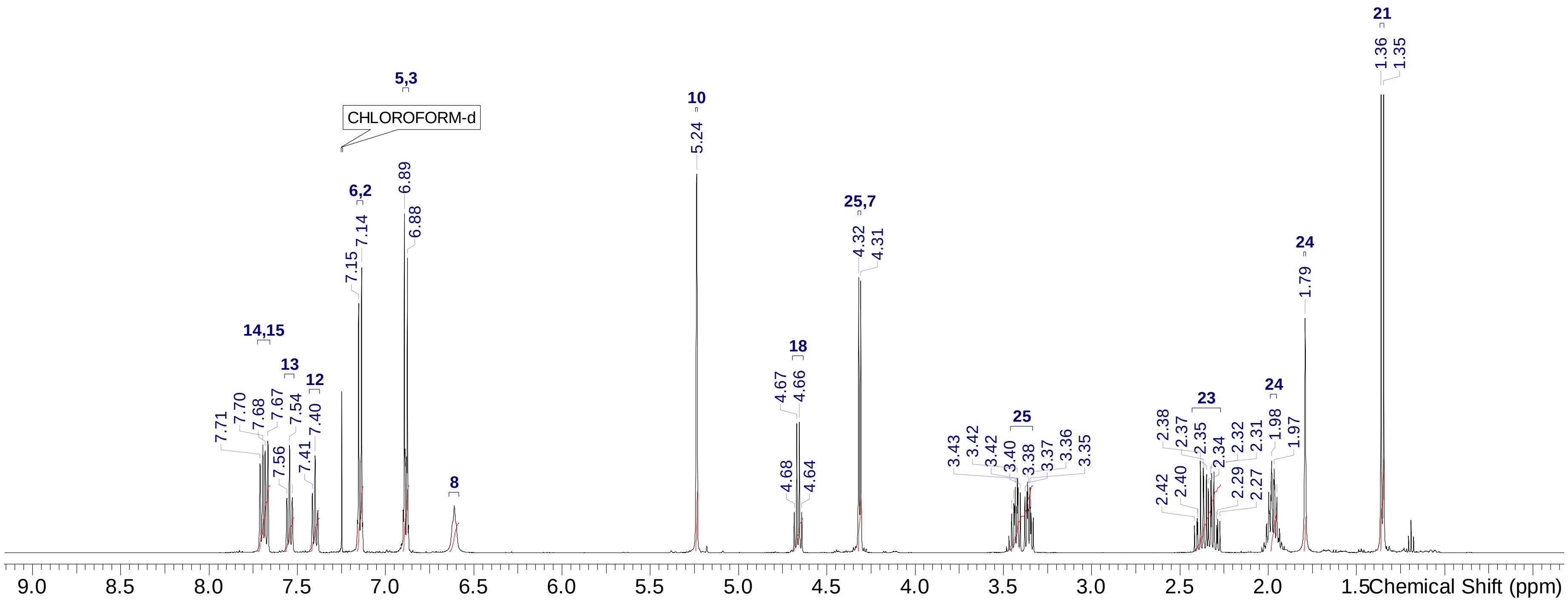

2-(2-oxopyrrolidin-1-yl)-*N*-(4-((2-(trifluoromethyl)benzyl)oxy)benzyl)propanamide (37) – ^{13}C NMR

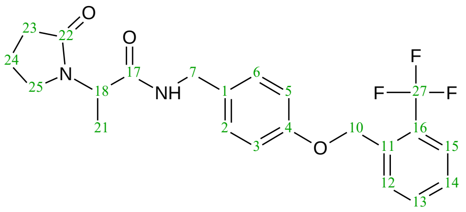

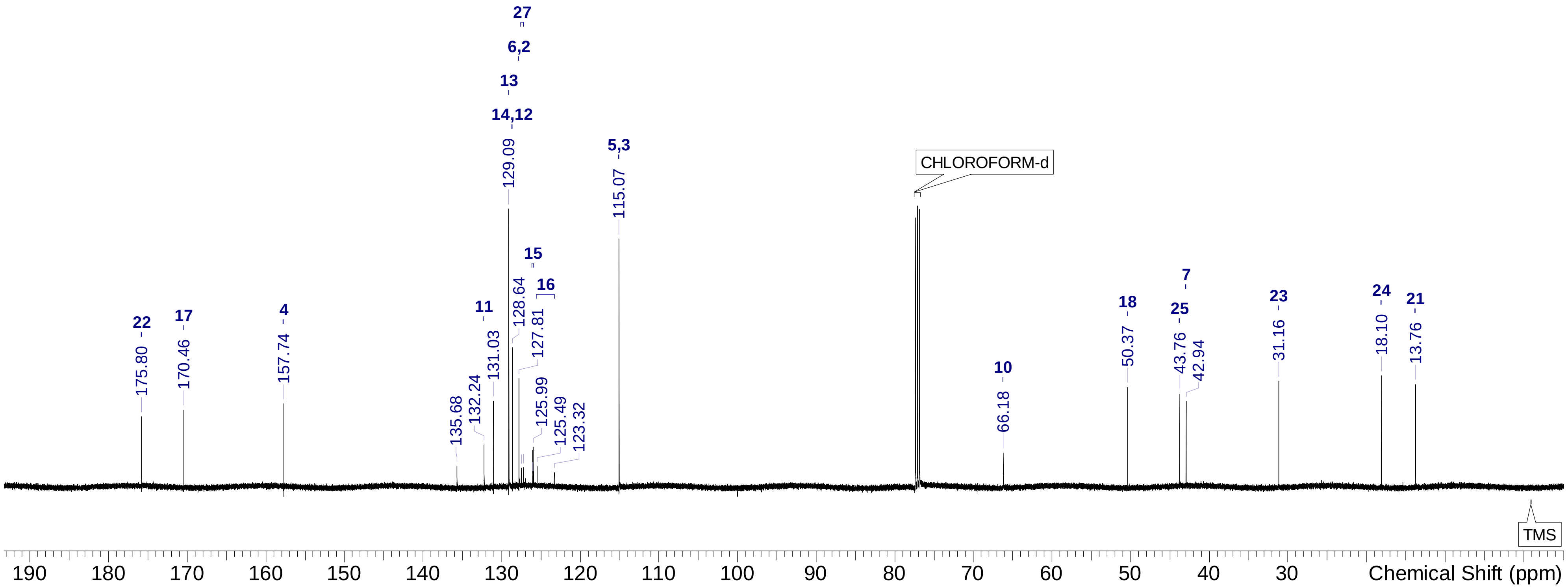

2-(2-oxopyrrolidin-1-yl)-*N*-(4-((3-(trifluoromethyl)benzyl)oxy)benzyl)propanamide (38) – ^{1}H NMR

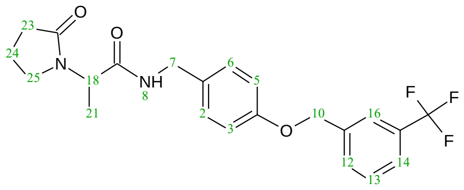

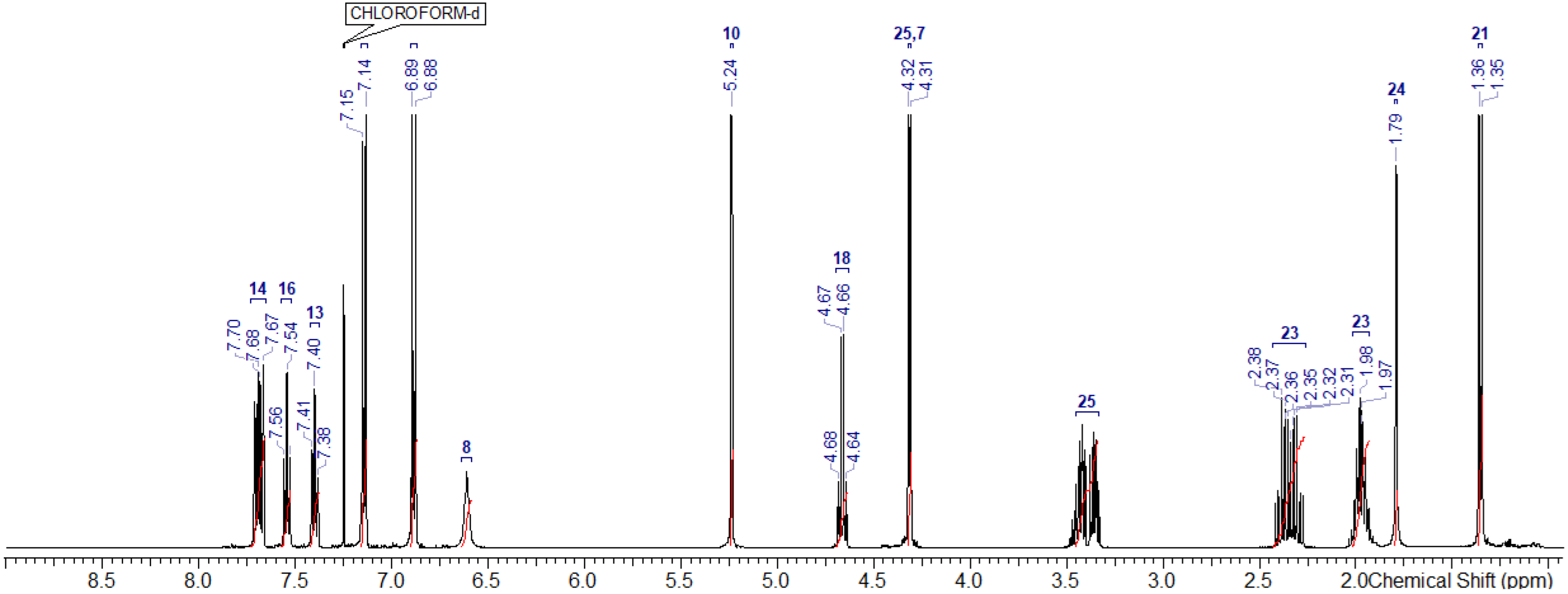

2-(2-oxopyrrolidin-1-yl)-*N*-(4-((4-(trifluoromethyl)benzyl)oxy)benzyl)propanamide (38) – ^{13}C NMR

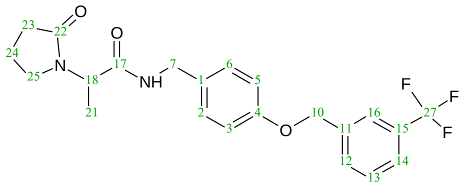

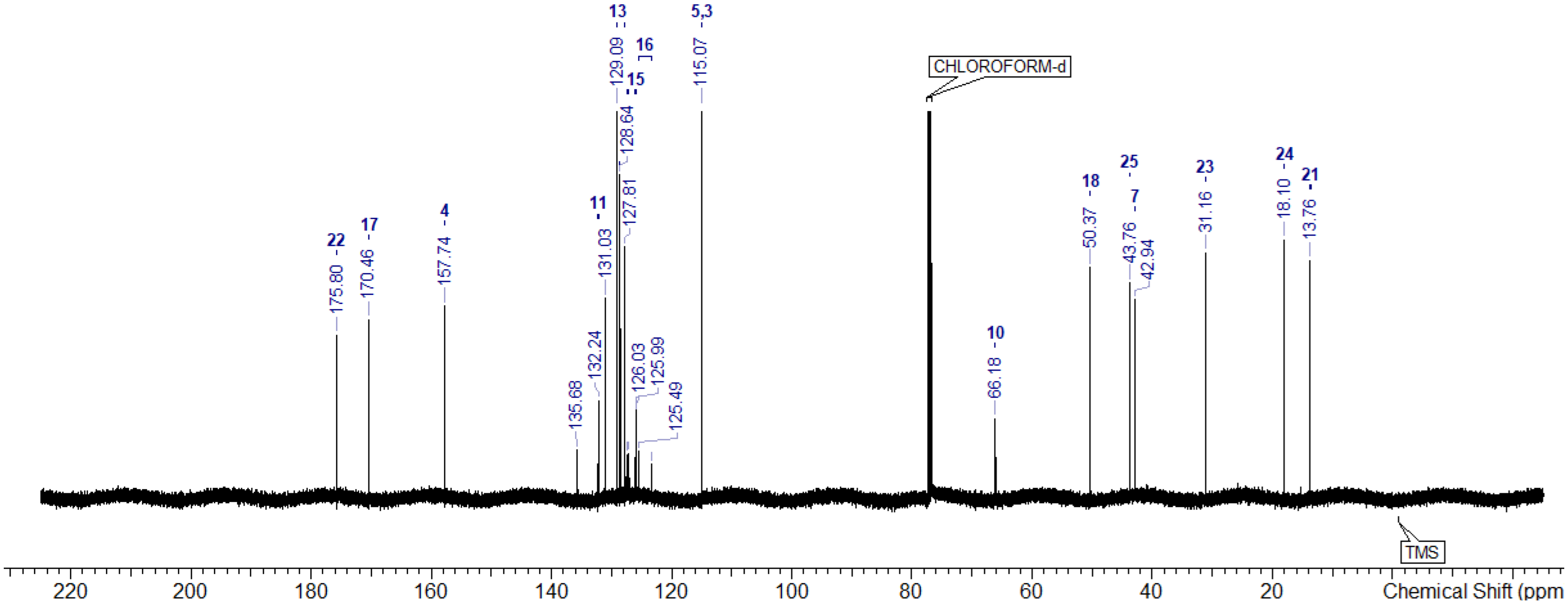

2-(2-oxopyrrolidin-1-yl)-*N*-(4-((4-(trifluoromethoxy)benzyl)oxy)benzyl)propanamide (39) – ^{1}H NMR

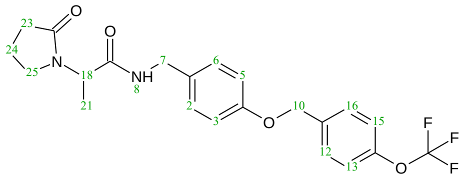

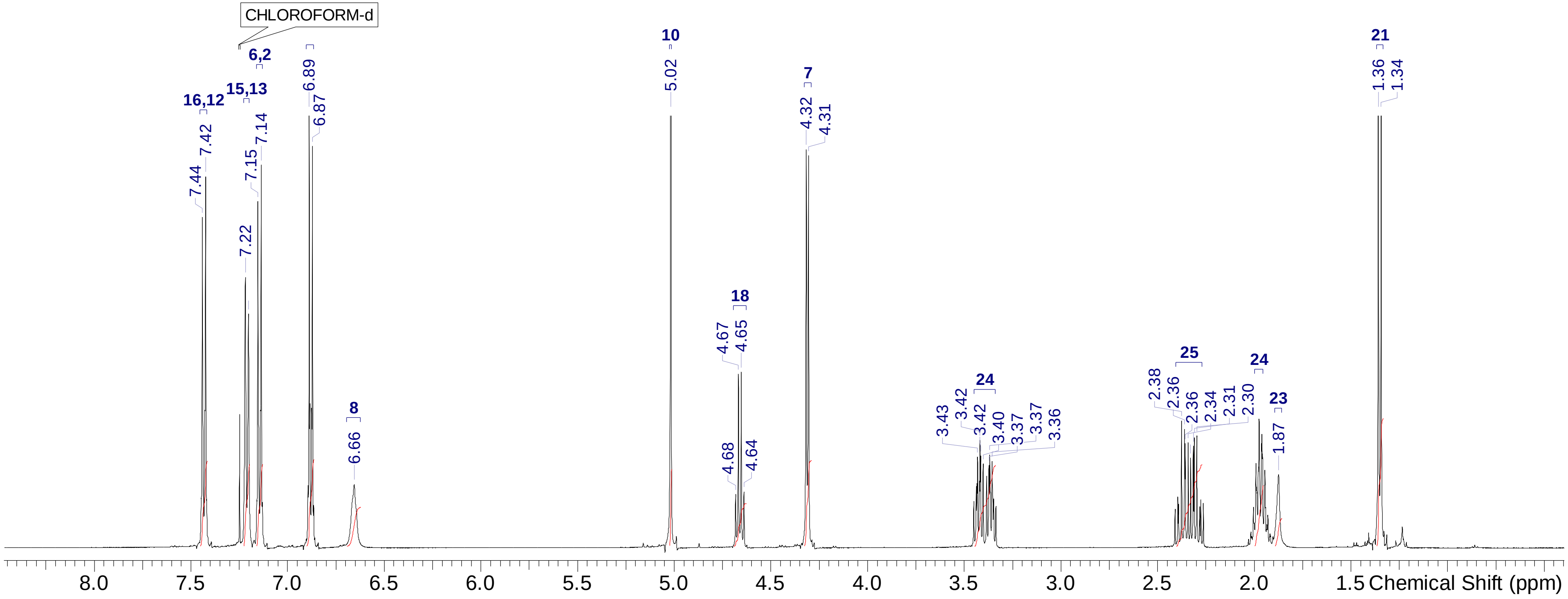

2-(2-oxopyrrolidin-1-yl)-*N*-(4-((4-(trifluoromethoxy)benzyl)oxy)benzyl)propanamide (39) – ^{13}C NMR

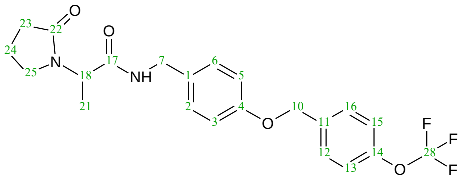

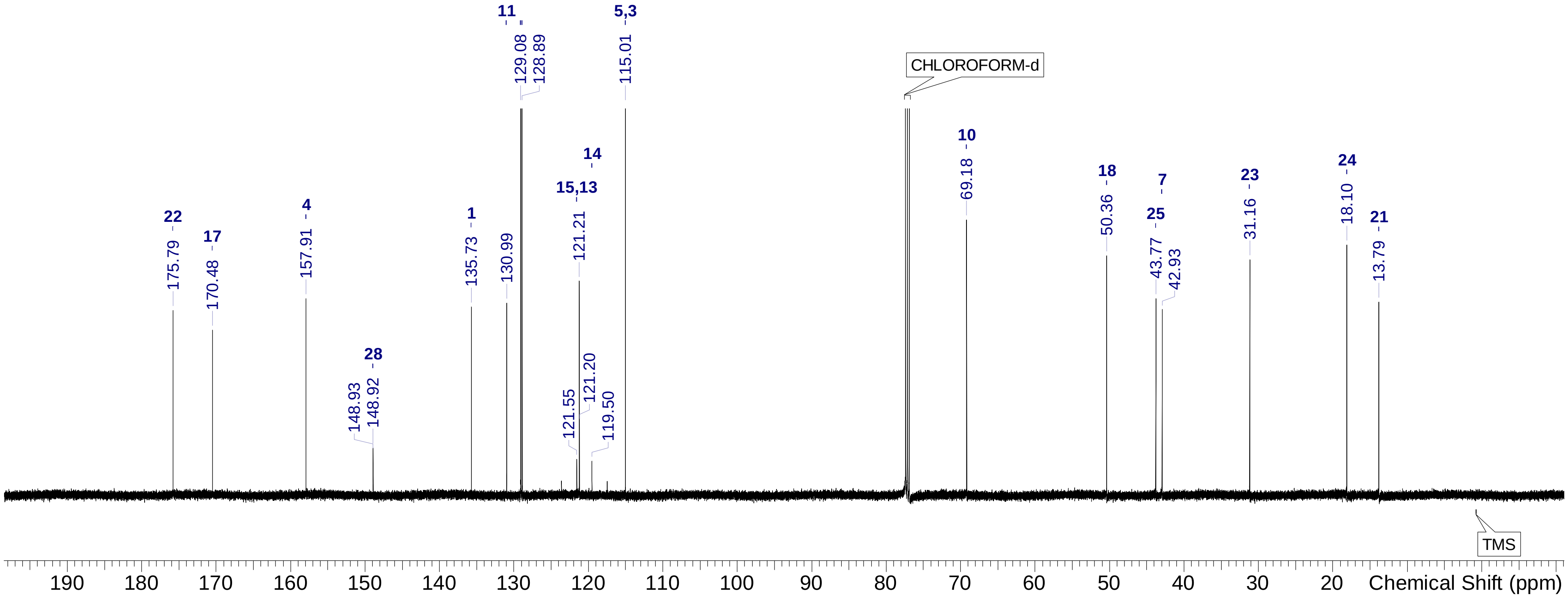

Supplement: Supplementary file 1 [file ijms-25-09861-s001.zip › ijms-3188005-supplementary.pdf]
